# 14-Residue peptaibol velutibol A from *Trichoderma velutinum*: its structural and cytotoxic evaluation†

**DOI:** 10.1039/d0ra05780k

**Published:** 2020-08-24

**Authors:** Varun Pratap Singh, Anup Singh Pathania, Manoj Kushwaha, Samsher Singh, Vandana Sharma, Fayaz A. Malik, Inshad A. Khan, Anil Kumar, Deepika Singh, Ram A. Vishwakarma

**Affiliations:** Medicinal Chemistry Division, CSIR-Indian Institute of Integrative Medicine Canal Road Jammu 180 001 India dsingh@iiim.res.in deeps.csir@gmail.com ram@iiim.res.in; Department of Biotechnology, Faculty of Sciences, Shri Mata Vaishno Devi University Katra Jammu and Kashmir 182320 India; Pharmacology Division, CSIR-Indian Institute of Integrative Medicine Canal Road Jammu 180 001 India; Quality Control & Quality Assurance Division, CSIR-Indian Institute of Integrative Medicine Canal Road Jammu 180 001 India; Clinical Microbiology Division, CSIR-Indian Institute of Integrative Medicine Canal Road Jammu 180 001 India; Department of Microbiology, Central University Rajasthan-305 817 India; Academy of Scientific and Innovative Research Jammu 180001 India

## Abstract

Velutibol A (1), a new 14-residue peptaibol was isolated from the Himalayan cold habitat fungus *Trichoderma velutinum*. The structural characterization was carried out by 1D and 2D NMR studies, and tandem mass studies, and Marfey's method aided in determining the stereochemistry of the amino acids. The CD analysis revealed folding of the peptide in a 3_10_-helical conformation. The intramolecular H-bonding was determined by an NMR-VT experiment. Cytotoxic evaluation was carried out against a panel of cancer cell lines. The cell cycle assay was carried out on human myeloid leukaemia (HL-60) cells and revealed the formation of apoptotic bodies and DNA damage in a dose-dependent manner. Three other peptaibols namely velutibol B (2), velutibol C (3), and velutibol D (4) were also isolated in trace amounts from the psychotropic fungus and characterized through tandem mass spectroscopy and Marfey's analysis.

## Abstract 2

Velutibol A (1), a new 14-residue peptaibol isolated from the Himalayan cold habitat fungus *Trichoderma velutinum*.

## Graphical abstract

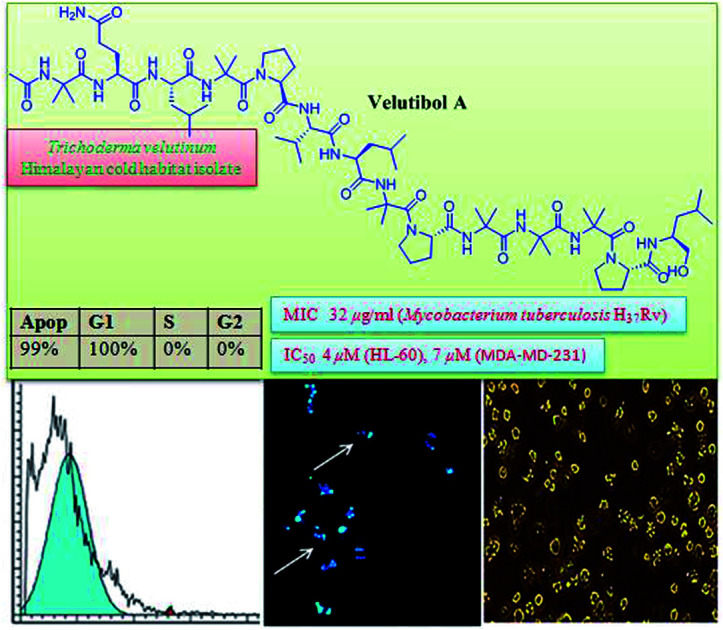

## Introduction

Peptaibols belong to the class of non-ribosomal peptides comprising non-proteinogenic amino acid residues including α-aminoisobutyric acid (Aib), ethyl norvaline, isovaline, hydroxylproline *etc.* The N-terminus in these peptides is acetylated and the C-terminus residue is an amino alcohol.^1–7^ They commonly exhibit various biological activities owing to their ion-channel formation,^8–10^ and membranolytic^11^ properties in modifying biological membranes. This includes activities like cytotoxic,^2,12^ neuroleptic,^13–15^ anthelmintic,^16^ hemolytic,^17^ anti-tubercular,^18^ antifungal,^19–21,56^ and antibacterial^20–22^ activity and has also shown significant effects against various phytopathogens.^23,24^ Their amino acid length varies from 5–20 amino acids which are classified into 9 subfamilies (SF1–SF9) depending upon their sequence, substitution pattern, and structure.^8^ Among these subfamilies, the SF1 (18–20 residues), SF4 (11 or 14 residues), SF5 (lipopeptaibols) and SF9 (7 residues) group of peptaibols are produced mainly from fungal genus *Trichoderma*.^25^ With more than 250 species of fungal genus *Trichoderma*,^26,27^ the majority of them are peptaibol producers,^28^ which include strains obtained from soil,^4,29^ and endophytic,^30^ and marine sources.^31,32^ Recently, we have reported lipopeptaibols from fungus *T. velutinum* from the soils of Himalayan cold habitat.^33^ There are almost 1000 peptaibols reported to date.^28,34,35^ They display microheterogeneity in character *i.e.* containing closely related peptides with limited sequence variation in the natural mixture.^36,37^ Herein, we found few 14-residue peptaibols of SF4 group with very little sequence variation. To the best of our knowledge, there are more than 70 sequences of peptaibols with 14 residues are reported belonging mainly to SF4 group except bergofungin D^8,38^ a peptaibol of SF2 group. All the 14 residue peptaibols of SF4 group contains three Aib-^L^Pro dipeptide motifs at 4th, 8th and 12th positions with no aromatic amino acid in the sequence^39–44^ whereas the SF2 group does not have the Aib-^L^Pro dipeptide motifs but contains phenylalaninol at C-terminus.^8^ These peptaibols with rich Aib motifs have been reported to fold into α-helix,^45^ 3_10_-helix^46,47^ and β-bend ribbon spiral conformation.^48^ These 14-residue peptaibols are reported to have antibacterial,^38,39^ antifungal,^39^ and amyloid β-peptide formation inhibitor^41^ activities.

**Fig. 1:**
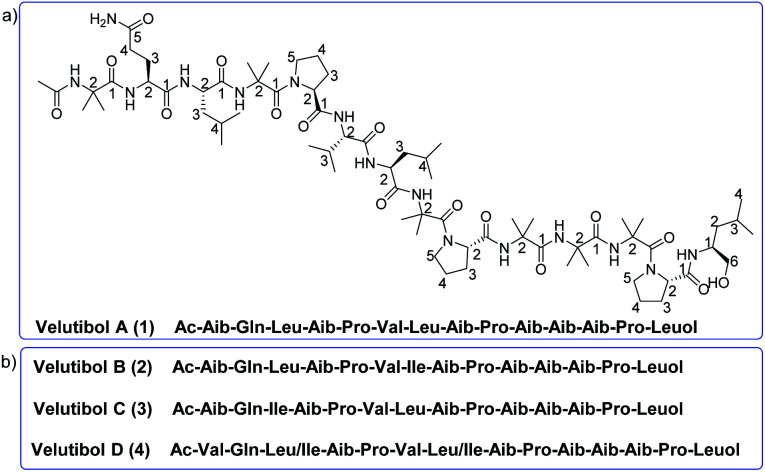
(a) Structure of velutibol A (1); (b) plausible sequence of velutibol B–D (2–4).

In our previous report, four lipopeptaibols namely lipovelutibols A–D from fungus *T. velutinum* were isolated from soils of Himalayan cold habitat.^33^ In continuing the exploration of peptaibiotics from *T. velutinum*, we herein, report isolation, structure elucidation, conformational analysis along with cytotoxic and anti-tubercular evaluation of peptaibol velutibol A (1) (Fig. 1a). As per our knowledge, this study represents the first report of 14-residue peptaibol having cytotoxic and weak anti-tubercular activity. In addition to 1, we herein, also provide probable structures of three other 14-residue peptaibols namely velutibol B–D (2–4) as shown in Fig. 1b, isolated in a trace amount and thus characterized through HRESIMS/MS studies and Marfey's analysis using LCMS.

## Results and discussion

The chromatographic purification of the ethyl acetate extract was carried out through RP-HPLC, which gave 15 fractions (Fig. S1, ESI†). The re-purification of fraction-6, 7, 8 and 10 gave four lipopeptaibols as described previously.^33^ The Fr-6 and Fr-7 also contained mixtures of peptides with higher *m*/*z* values, which were further purified through RP-HPLC to get the peptaibols (1–4) as discussed in the Experimental section (Fig. S2 and S3, ESI†).

The High Resolution Mass Spectroscopy (HRMS) of velutibol A (1) gave the molecular formula as C_69_H_119_N_15_O_16_ showing *m*/*z* 1414.9031 [M + H]^+^ (calcd 1414.9032) and 1436.8857 [M + Na]^+^ (calcd 1436.8851) (Fig. S4, ESI†) indicating eighteen double bond equivalents. The 1D proton NMR spectrum showed 13 NH protons in the region ranging at *δ*_H_ 6.5–9.5 ppm, 8 α-H ranging at *δ*_H_ 3.7–4.5 ppm and presence of acetyl protons at *δ*_H_ 1.9 ppm. It also showed the presence of 36 protons signals for 12 methyls in region *δ*_H_ 1.2–1.5 ppm and 24 protons signals corresponding to 8 methyls at *δ*_H_ 0.7–1.0 ppm. The close observation of 1D carbon NMR experiments including ^13^C and DEPT spectra revealed 69 carbon signals, which were assigned for 15 carbonyls, 6 carbons (*α*,*α*-disubstituted carbon), 12 methines (distinguished in DEPT-90), 15 methylene (including 3 methylene merged in DMSO signal as observed in DEPT-135) and 21 methyl carbons (including signal at *δ*_C_ 23.66 ppm for 2 carbons and one methyl carbon at *δ*_C_ 25.61 ppm as observed in DEPT-135) (Table 1) (Fig. S5–S8, ESI†).

**Table 1:** Proton and carbon NMR value of 1 in DMSO-*d*_6_

Residue		Type	^13^C (*δ*)	^1^H (*δ*), (mult. *J* in Hz)	Residue		Type	^13^C (*δ*)	^1^H (*δ*), (mult. *J* in Hz)
Ac	1	C=O	171.2	—		5	CH_3_	21.5	0.79 (m)^b^
	2	CH_3_	22.9	1.90, (s)		6	CH_3_	22.8	0.85 (d, 6.8)
Aib-1	1	C=O	176.3	—			NH		7.21 (d, 8.4)
	2	C	55.9	—	Aib-8	1	C=O	172.1	
	3	CH_3_	23.6	1.34 (s)		2	C	55.6	
	4	CH_3_	26.5	1.36 (s)^b^		3	CH_3_	23.7^b^	1.40 (s)
		NH		8.73 (s)		4	CH_3_	25.6	1.29 (s)
Gln-2	1	C=O	172.6				NH		7.63 (s)
	2	CH	55.0	3.96 (m)	Pro-9	1	C=O	172.9	
	3	CH_2_	25.0	1.97 (m)		2	CH	63.2	4.13 (t, 8.4)
	4	CH_2_	31.1	2.29 (m), 2.18 (m)		3	CH_2_	28.5	2.20(m), 1.61 (m)
	5	C=O	174.5			4	CH_2_	25.1	1.86 (m), 1.74(m)
		NH_2_		7.48 (br s), 6.95 (br s)		5	CH_2_	48.1	3.68(m), 3.58 (m)
		NH		8.93 (d, 5.2)	Aib-10	1	C=O	173.6	
Leu-3	1	C=O	171.7			2	C	56.1	
	2	CH	51.2	4.18 (m)		3	CH_3_	25.6^a^	1.36 (s)^b^
	3	CH_2_	39.1^a^	1.78 (m), 1.47 (m)		4	CH_3_	23.6	1.36 (s)^b^
	4	CH	24.2	1.57 (m)			NH		7.84 (s)
	5	CH_3_	22.8	0.89 (d, 6.8)	Aib-11	1	C=O	175.5	
	6	CH_3_	20.7	0.79 (m)^b^		2	C	56.0	
		NH		7.88 (d, 8.4)		3	CH_3_	26.5	1.36 (s)^b^
Aib-4	1	C=O	173.6			4	CH_3_	23.6	1.44 (s)
	2	C	55.8				NH		7.34 (s)
	3	CH_3_	23.4	1.49 (s)	Aib-12	1	C=O	171.6	
	4	CH_3_	25.4	1.36 (s)^b^		2	C	55.7	
		NH		7.93 (s)		3	CH_3_	23.7^b^	1.39 (s)
Pro-5	1	C=O	172.7			4	CH_3_	25.0	1.36 (s)^b^
	2	CH	63.1	4.23 (m)			NH		7.58 (s)
	3	CH_2_	28.7	2.18 (m), 2.08 (m)	Pro-13	1	C=O	170.9	
	4	CH_2_	25.4^a^	1.86 (m), 1.74 (m)		2	CH	61.9	4.23 (m)
	5	CH_2_	48.4	3.68 (m), 3.58 (m)		3	CH_2_	28.1	2.18(m), 2.08 (m)
Val-6	1	C=O	171.4			4	CH_2_	25.6^a^	1.86(m), 1.74 (m)
	2	CH	60.1	3.85 (t, 8.0)		5	CH_2_	48.4	3.68(m), 3.58 (m)
	3	CH	28.8	2.18 (m)	Leuol	1	CH	48.4	3.77 (m)
	4	CH_3_	19.0	0.97 (d, 6.4)		2	CH_2_	39.3^a^	1.37 (m)
	5	CH_3_	18.9	0.91 (d, 6.8)		3	CH	23.8	1.67 (m)
		NH		7.28 (d, 8.4)		4	CH_3_	22.8	0.82 (m)^b^
Leu-7	1	C=O	172.2			5	CH_3_	20.2	0.79 (m)^b^
	2	CH	51.2	4.29 (m)		6	*CH*_2_OH	63.9	3.27(m), 3.18 (m)
	3	CH_2_	39.6^a^	1.54 (m)			NH		7.15 (d, 9.6)
	4	CH	24.4	1.67 (m)			OH		4.23 (m)

a From DEPT-135.

b Signal overlap.

**Fig. 2:**
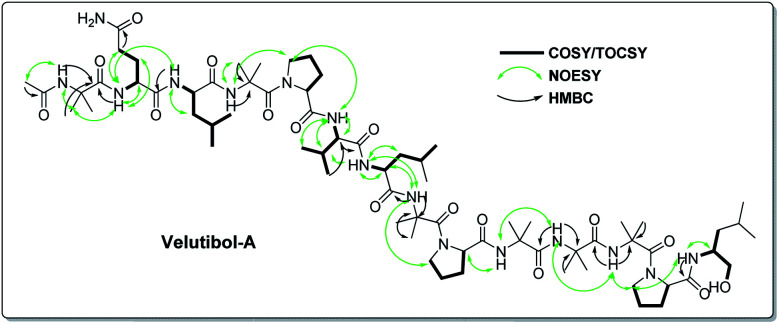
The key correlation from 2D NMR (COSY, TOCSY, NOESY and HMBC) of compound 1.

The detailed examination of 1D along with 2D experiments such as COSY, TOCSY, NOESY and HMBC data elucidated the structure as 1, which comprises 13 amino acids *viz.* six α-aminoisobutyric acids (Aib), two leucines (Leu), one valine (Val), three prolines (Pro) and one glutamine (Gln) and an amino alcohol leucinol (Leuol) at the C-terminus. The 14-residue sequence was found to have acetylation at the N-terminus (Fig. 2). For instance, the 2D experiments such as COSY and TOCSY showed three Pro, two Leu, one Leuol, and one Val moieties in the molecule. The amino acid Gln was recognized by the presence of correlation between two NHs having broad singlets at *δ*_H_ 6.95 and 7.48 in COSY and TOCSY experiments along with the correlations obtained from α-H, β-H_2_, and γ-H_2_. The presence of six Aib was revealed by HMBC correlations as shown in Fig. 2 (Fig. S9–S13, ESI†).

**Fig. 3:**
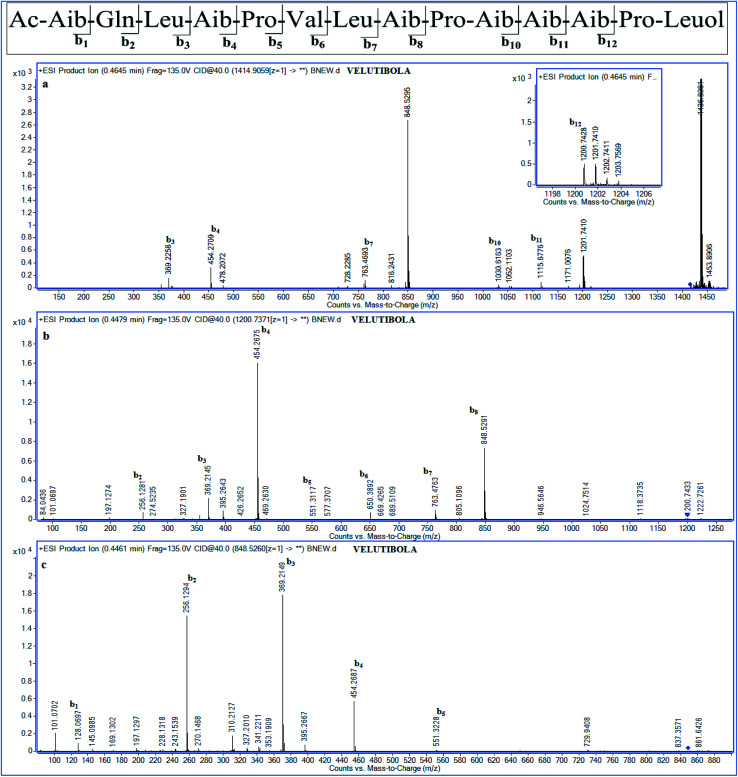
MS^*n*^ studies of compound 1 (a) MS^2^ at *m*/*z* 1414.9059 [M + H]^+^; (b) MS^3^ for daughter ion *b*_12_ at *m*/*z* 1200.7371 (c) MS^3^ for daughter ion *b*_8_ at *m*/*z* 848.526.

The peptide primary sequence was determined using NOESY correlations. The NOE experiment revealed acetyl moiety at N-terminus of Aib-1 *i.e.* α-H_3_ (*δ*_H_ 1.90 acetyl)/NH-1 (*δ*_H_ 8.73, Aib-1). Thus, the sequential assignment was observed through β-H_3_ (*δ*_H_ 1.36, Aib-1)/NH-2 (*δ*_H_ 8.93, Gln-2), NH-2/NH-3 (*δ*_H_ 7.88, Leu-3), NH-4 (*δ*_H_ 7.93, Aib-4)/*δ*-H_2_-5 (*δ*_H_ 3.68, 3.58, Pro-5), *δ*-H_2_-5/NH-5 (*δ*_H_ 7.28, Val-6), β-H-6 (*δ*_H_ 2.18, Val-6)/NH-6 (*δ*_H_ 7.21, Leu-7), NH-6/NH-7 (*δ*_H_ 7.63, Aib-8), NH-8/*δ*-H_2_-9 (*δ*_H_ 3.68, 3.58, Pro-9), α-H-9 (*δ*_H_ 4.13, Pro-9)/NH-9 (*δ*_H_ 7.84, Aib-10), NH-9/NH-10 (*δ*_H_ 7.34, Aib-11), NH-10/NH-11 (*δ*_H_ 7.58, Aib-12), NH-11/*δ*-H_2_-13 (*δ*_H_ 3.68, 3.58, Pro-13), *δ*-H_2_-13/NH-12 (*δ*_H_ 7.15, Leuol). These observations along with HMBC correlations as shown in Fig. 2 (Tables S1 and S2, ESI†) gave the primary sequence of 1 as Ac-Aib-Gln-Leu-Aib-Pro-Val-Leu-Aib-Pro-Aib-Aib-Aib-Pro-Leuol. The MS/MS studies for [M + H]^+^at *m*/*z* 1414.90 showed prominent *b*-ions at *m*/*z* 1200.74 (*b*_12_), 848.52 (*b*_8_) and 454.26 (*b*_4_), an indication of easily labile Aib–Pro bond in ESI fragmentation, along with other *b*-ions at 1115.68 (*b*_11_), 1030.62 (*b*_10_), 763.47 (*b*_7_) and 369.22 (*b*_3_). Further, MS/MS of 1200.74 (*b*_12_) gave daughter ions at 650.39 (*b*_6_), 551.31 (*b*_5_) and 256.13 (*b*_2_). The daughter ion 128.07 (*b*_1_) was observed *via* MS/MS of 848.53 (*b*_8_) (Fig. 3). The MS/MS data in combination with the NOESY and HMBC correlations established the sequence of compound 1 as Ac-Aib-Gln-Leu-Aib-Pro-Val-Leu-Aib-Pro-Aib-Aib-Aib-Pro-Leucinol.

The advanced Marfey's method^49^ was used for the determination of stereochemistry. Acid hydrolysis of the compound 1 was done and derivatized with 1-fluoro-2,4-dinitrophenyl-5-l-alaninamide (^L^FDAA). Likewise, both the d- and l-amino acid standards were derivatized with ^L^FDAA. The amino acid glutamine gets converted into glutamic acid on acid hydrolysis,^35^ thus ^L^Glu and ^D^Glu was derivatized for stereochemical determination of Gln. The amino acid standards thus obtained were compared with l-FDAA derivatives of acid hydrolyzed compounds using LCMS instrument. The retention times for standard amino acid-^L^FDAA derivatives were found to be ^L^Glu/^D^Glu (21.0/21.8), ^L^Pro/^D^Pro (23.6/24.4), ^L^Val/^D^Val (26.9/29.5), ^L^Leu/^D^Leu (29.8/32.3) and ^L^Leuol/^D^Leuol (29.4/32.6) and for the acid hydrolyzed-^L^FDAA derivatives were 21.0, 23.5, 27.0, 29.4 and 29.8 for ^L^Glu/^L^Gln, ^L^Pro, ^L^Val, ^L^Leuol, and ^L^Leu respectively. Thus, the advanced Marfey's analysis carried out for 1 indicated the l-form of Glu/Gln, Leu, Val, Pro and Leuol (Fig. S14, ESI†).

As the compounds velutibol B–D (2–4) were isolated in very low amounts (0.3–0.1 mg) the structural characterization could not be established through NMR. The characterization of velutibol B–D (2–4) was established through tandem mass spectrometry utilizing HRMS. The HRMS of 2 and 3 gave the molecular formula as C_69_H_119_N_15_O_16_ showing *m*/*z* 1414.9060 [M + H]^+^ and 1414.9022 [M + H]^+^ respectively (calcd 1414.9032). The probable primary sequence of these peptides has been given in Table 2. The compound 2 and 3 have similar *m*/*z* values as that of 1. The sequence of 2 was elucidated by tandem mass spectroscopy. The MS/MS scan of 1414.903 [M + H]^+^ which gave *b*-ions at *m*/*z* 256.13 (*b*_2_), 369.21 (*b*_3_), 454.27 (*b*_4_), 551.31 (*b*_5_), 650.38 (*b*_6_), 763.47 (*b*_7_), 848.52 (*b*_8_), 945.57 (*b*_9_), 1030.63 (*b*_10_), 1115.67 (*b*_11_), and 1200.73 (*b*_12_). The *b*_1_ ion (*m*/*z* 128.07) was found from the MS/MS scan for the peak of *m*/*z* 454.27 (Fig. S20a and b, ESI†). This gave the sequence as Ac-Aib-Gln-Lxx-Aib-Pro-Vxx-Lxx-Aib-Pro-Aib-Aib-Aib-Pro-Lxxol. The similar sequence was observed for compound 3. The daughter ions obtained from tandem mass spectroscopy of [M + H]^+^ ion of 3 are given in Table 2 (Fig. S23a–c, ESI†). The molecular formula for compound 4 was found to be C_70_H_121_N_15_O_16_ showing *m*/*z* 1428.9188 [M + H (calcd 1428.9188). The tandem mass studies for compound 4 revealed a difference of 14 Da for *b*_1_ ion as *m*/*z* of 142.09 corresponding for Ac-Vxx moiety. The daughter ions obtained from the fragmentation of compound 4 having *m*/*z* 1428.9191 [M + H]^+^ were 468.28 (*b*_4_), 862.54 (*b*_8_), 1044.65 (*b*_10_), 1129.71 (*b*_11_), and 1214.75 (*b*_12_). Further, MS/MS for *m*/*z* 1214.7532 (*b*_12_) gave daughter ions as 142.09 (*b*_1_), 270.14 (*b*_2_), 383.23 (*b*_3_), 565.34 (*b*_5_), 644.40 (*b*_6_), 777.49 (*b*_7_) and 969.60 (*b*_9_) along with other *b* ions (Fig. S26a and b, ESI†). This gave the sequence as Ac-Vxx-Gln-Lxx-Aib-Pro-Vxx-Lxx-Aib-Pro-Aib-Aib-Aib-Pro-Lxxol. These compounds were eluted at different retention times during purification and subsequently showed different retention times on further HPLC analysis (Fig. S27, ESI†), which reflected that these compounds were different with similar *m*/*z* values.

**Table 2:** Plausible sequence of 2–4 with their respective daughter ions from MS/MS studies

Compounds	Sequence	*m*/*z*^a^	*t*_R_ (min)	Respective *b*-ions
Velutibol B (2)	Ac-Aib-Gln-Lxx-Aib-Pro-Vxx-Lxx-Aib-Pro-Aib-Aib-Aib-Pro-Lxxol	1414.9060, 1436.8882	43.5	1200.73, 1115.67, 1030.63, 945.57, 848.52, 763.47, 650.38, 551.31, 454.27, 369.21, 256.13 and 128.07
Velutibol C (3)	Ac-Aib-Gln-Lxx-Aib-Pro-Vxx-Lxx-Aib-Pro-Aib-Aib-Aib-Pro-Lxxol	1414.9022, 1436.8835	44.3	1200.74, 1115.68, 1030.62, 848.52, 763.47, 650.38, 551.31, 454.27, 369.21, 256.13 and 128.07
Velutibol D (4)	Ac-Vxx-Gln-Lxx-Aib-Pro-Vxx-Lxx-Aib-Pro-Aib-Aib-Aib-Pro-Lxxol	1428.9188, 1450.9013	45.0	1214.75, 1129.71, 1044.65, 959.60, 862.54, 777.49, 644.40, 565.34, 468.28, 383.23, 270.14 and 142.09

a HRMS observed values for [M + H]^+^ and [M + Na]^+^, *t*_R_ = retention time.

The Marfey's analysis of the compounds 2–4 were also carried at a scale of 20 μg owing to their low quantities. It revealed presence of ^L^Gln/^L^Glu, ^L^Pro, ^L^Val, ^L^Ile, ^L^Leu and ^L^Leuol. The extracted ion chromatogram (EIC) for *m*/*z* 368.0, 370.0, 384.0 and 400.0 gave one, two, two and one peaks respectively for compound 2–4. The respective amino acid derivatives were found to be in-line with the EIC obtained for compounds 2–4 (Fig. S28–S33, ESI†). The amino acid ^L^Ile was distinguished from l-*allo*-isoleucine (^L^*allo*-Ile) *via* using chiral LCMS. The three peptaibols studied here were found to have ^L^Ile in their respective sequence (Fig. S34, ESI†). Moreover, the compound 2 and 3 having similar primary sequence with same amino acids asserted that the amino acid Ile and Leu were probably exchanged in respective position. The close observation of MS/MS for 2 (Fig. S20b, ESI†) gave a *m*/*z* 327.20 for fragmentation of –CH(CH_3_)_2_ corresponding to Leu-3 similarly a *m*/*z* 341.22 for fragment –CH_2_CH_3_ corresponding to Ile-3 for 3 (Fig. S23b, ESI†). Thus, combining Marfey's analysis with tandem mass spectral analysis gave the sequence of 2, 3 and 4 as Ac-Aib-Gln-Leu-Aib-Pro-Val-Ile-Aib-Pro-Aib-Aib-Aib-Pro-Leuol, Ac-Aib-Gln-Ile-Aib-Pro-Val-Leu-Aib-Pro-Aib-Aib-Aib-Pro-Leuol and Ac-Val-Gln-Leu/Ile-Aib-Pro-Val-Leu/Ile-Aib-Pro-Aib-Aib-Aib-Pro-Leuol respectively.

**Fig. 4:**
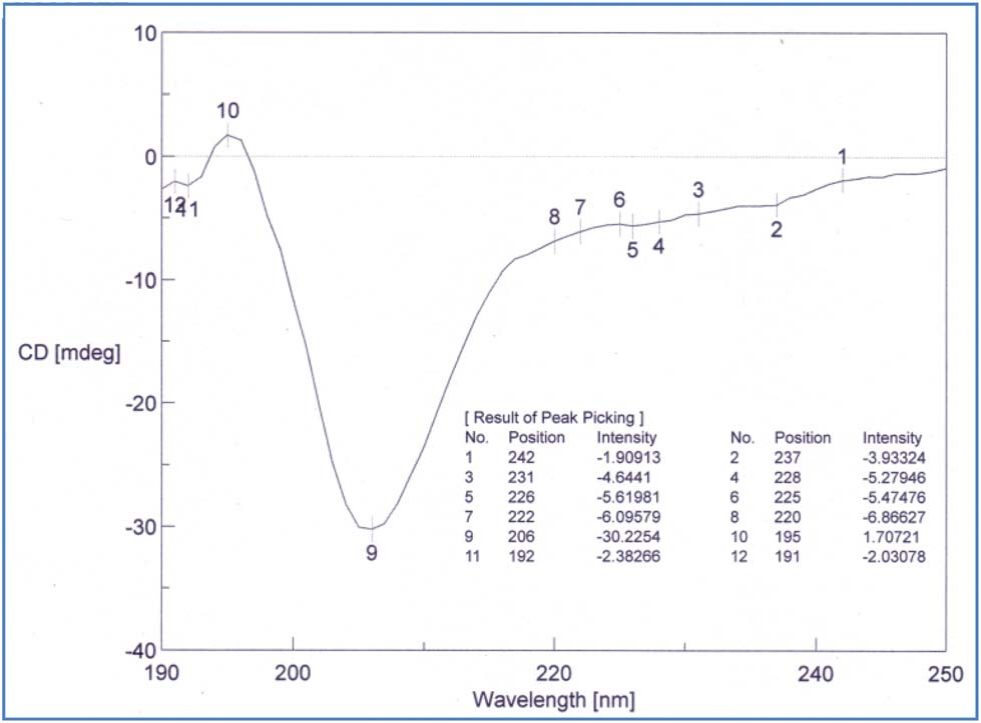
CD spectrum of compound 1 in methanol.

Conformational studies were performed through CD technique^50,51^ and NMR-VT (variable temperature).^52,53^ The CD spectra of the 1 in methanol were analyzed as shown in Fig. 4. The shapes of the CD pattern showed a −ive maxima at 206 nm and a −ive shoulder at 220 nm (in the vicinity of 222 nm). The ellipticity ratio *R* [*θ*_*T*_^220^]/[*θ*_*T*_^206^] was found to be 0.23. This clearly indicated that the compound 1 folds in a 3_10_-helix in methanol as the value for *R* was less than 0.50, which is known for the high propensity of 3_10_-helix.

**Fig. 5:**
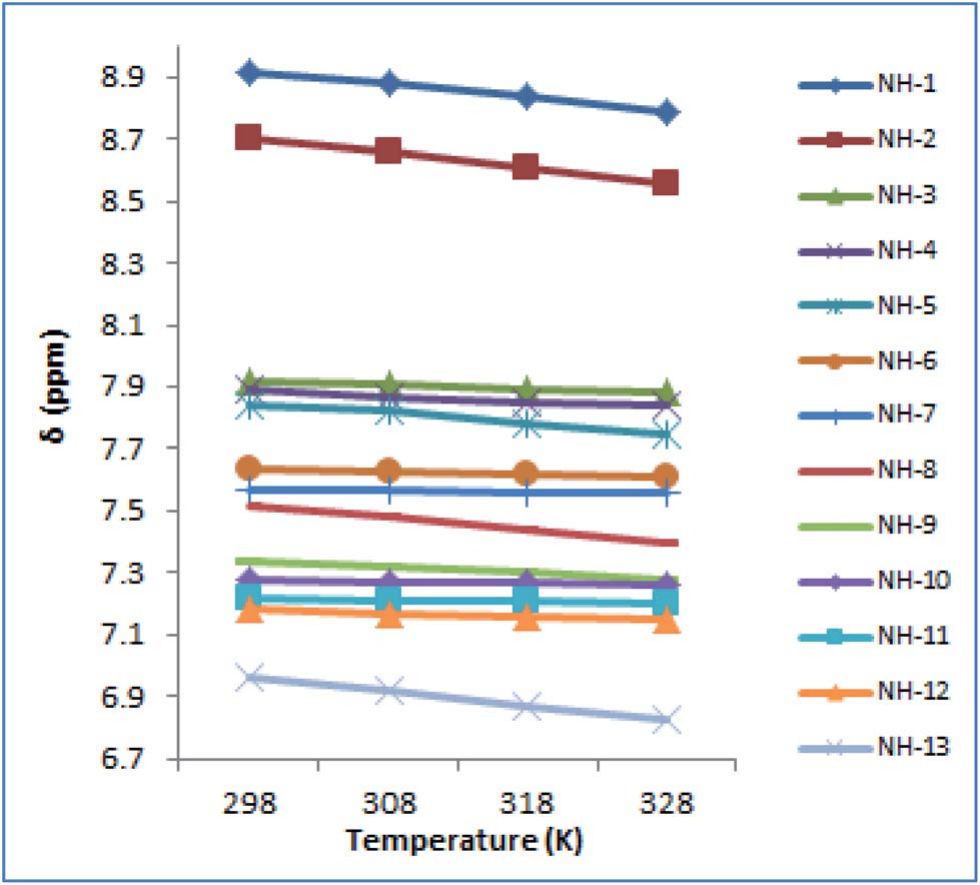
NMR-VT experiment performed for compound 1 at four different temperatures *i.e.* 298 K, 308 K, 318 K and 328 K.

The NMR-VT examined the manner in which the NH groups in the peptide behaved as a function of temperature.^52,53^^1^H NMR of peptaibol 1 was recorded at four different temperatures *i.e.* 298 K, 308 K, 318 K, 328 K in DMSO-*d*6. Out of the thirteen NH protons in the putative peptide, the chemical shifts of NH-1, NH-2, NH-5 along with amide NH_2_ of Gln (NH-8 and NH-13) were sensitive to heating while the other NH groups had shown very little change in chemical shift values as shown in Fig. 5. On the basis of these ^1^H NMR-VT experiment results (Fig. S35, ESI†), it was evident that NH-3 (Leu-3), NH-4 (Aib-4), NH-6 (Leu-7), NH-7 (Aib-8), NH-9 (Aib-10), NH-10 (Aib-11), NH-11 (Aib-12), and NH-12 (Leuol) were involved in intramolecular hydrogen bonding in peptaibol 1.

**Fig. 6:**
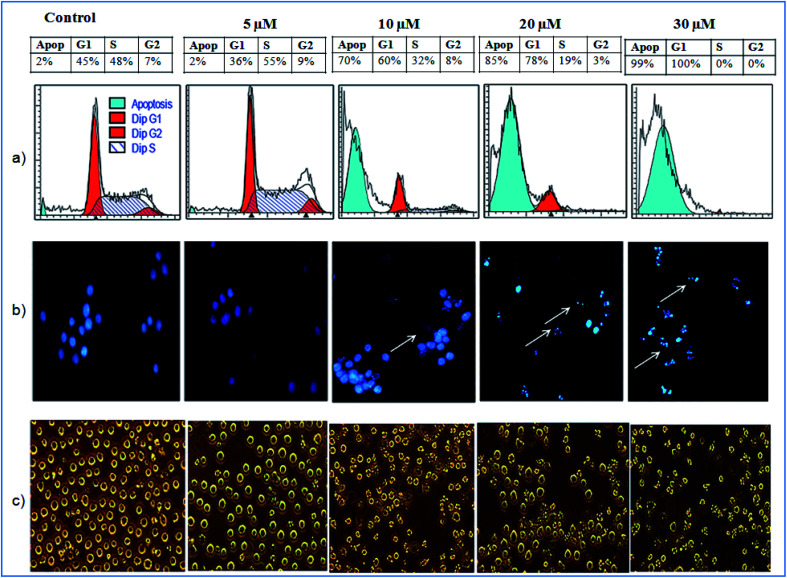
Effect of 1 (a) on cell cycle phase distribution; (b) on the morphology and (c) on the nuclear morphology of HL-60 cells.

**Fig. 7:**
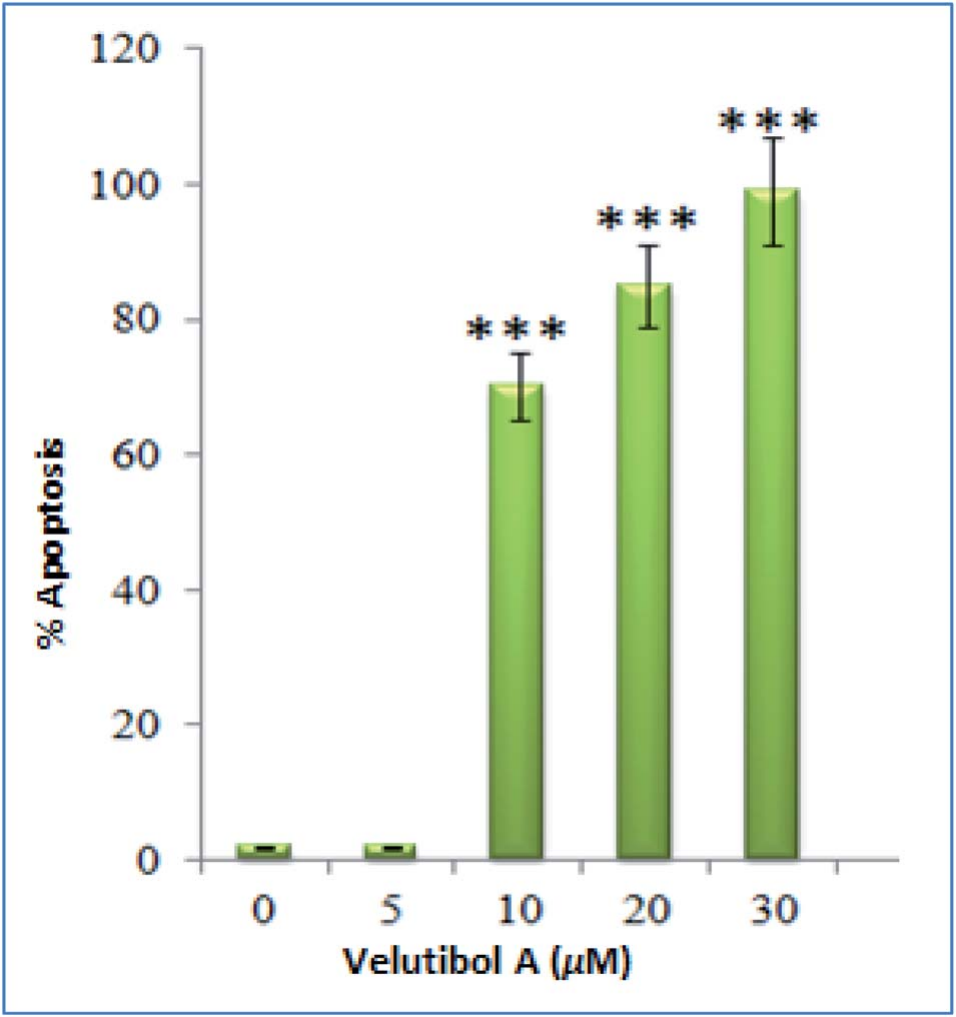
Graphical representation of cells undergoing apoptosis after treatment with compound 1. Columns, mean; bars, SD with ***p < 0.001 *versus* control.

The cytotoxicity testing of compound 1 was performed against various cell lines, which includes human lung cancer (A549), human colon cancer (LS-180), human myeloid leukemia (HL-60) and human breast cancer (MDA-MB-231) using MTT (3-(4,5-dimethylthiazole-2-yl)-2,5-diphenyltetrazolium bromide). Herein, paclitaxel was used as positive control for cytotoxic assay. The MTT assay showed inhibitory activity of compound 1 against MDA-MD-231 and HL-60 cancer cell lines having IC_50_ values of 7 μM and 4 μM respectively (Table 3). The IC_50_ for positive control was found to be in range of 2.0 to 6.8 nM against four cancer cell lines tested. Further, the cell-cycle assay was performed on HL-60 cell lines. The compound 1 was treated against HL-60 cells at concentrations 5, 10, 20 and 30 μM and incubated for 24 h. The compound 1 showed DNA damage in a dose-dependent manner. The HL-60 cells, with no treatment, showed non-significant 2% apoptotic DNA whereas the cells treated with 1 showed 70%, 85% and 99% apoptotic DNA population at 10, 20 and 30 μM concentrations respectively (Fig. 6a). Apoptotic bodies are small DNA fragments wrapped around the single membrane vesicles mainly formed when cells undergo apoptosis. These apoptotic bodies can be visualized under a fluorescent microscope by using DNA staining dyes such as 4′,6-diamidino-2-phenylindole (DAPI). The HL-60 cells were incubated with the compound 1 at different concentrations (5, 10, 20 and 30 μM) for 24 h. The apoptotic bodies (indicated by white arrows in Fig. 6b) were seen in cells treated with tested compound whereas healthy and round nuclei with no DNA fragmentation was observed with the untreated cells. Formation of apoptotic bodies increased in a dose-dependent manner. Similarly, the phase contrast microscopy of cells treated with 1 showed bled and distorted cells while the control cells were observed as healthy (Fig. 6c). The % apoptosis is also represented graphically in Fig. 7.

**Table 3:** Cytotoxic assay of 1 against various cell lines

	IC_50_^a^ ± standard deviation (μM)			
	A549	LS-180	HL-60	MDA-MB-231
1	23 ± 0.8	26 ± 1.1	4 ± 0.1	7 ± 0.2
Paclitaxel (nM)	6.2 ± 0.1	2.2 ± 0.1	1.8 ± 0.1	3.3 ± 0.1

a IC_50_ = half maximal inhibitory concentration. The experiments were conducted three times and data represented as mean ± standard deviation, with *p* value less than 0.05 calculated using Student *t*-test.

The compound 1 was also tested for anti-tubercular potential. The Minimum Inhibitory Concentration (MIC) of compound 1 was found to be 32 μg mL^−1^ against *Mycobacterium tuberculosis.* Rifampicin was used as a positive control with MIC 0.06 μg mL^−1^ (Fig. S36, ESI†).

## Experimental

### Materials

The chemicals used in the study such as Marfey's reagent (^L^FDAA), all the standard l and d-amino acids (except ^L^*allo*-Ile), propidium iodide (PI), RPMI-1640, 4′,6-diamidino-2-phenylindole (DAPI), and 3-(4,5-dimethylthiazole-2-yl)-2,5 diphenyltetrazolium bromide (MTT) were purchased from Sigma-Aldrich, USA. The amino acid ^L^*allo*-Ile was purchased from TCI Chemicals, Japan. The Middlebrook 7H9 broth was purchased from Difco Laboratories, Detroit, MI, USA, Tween 80 from Himedia, Mumbai, India and albumin dextrose catalase (ADC) from Becton Dickinson, Sparks, MD, USA. The HPLC grade solvents such as water, methanol and acetonitrile were purchased from Merck-India. The HL-60 cancer cell lines were purchased from ECACC, England. The cells were grown at 37 °C with 95% humidity in a CO_2_ incubator (Thermocon Electron Corporation, Houston, TX) in RPMI-1640 growth medium supplemented with 10% FCS and 1× antibiotic/antimycotic solution obtained from Gibco, USA. The *M. tuberculosis* H_37_Rv (ATCC 27294) was obtained from American Type Culture Collection, Manassas, VA, USA. The FT-IR spectroscopy was carried out in chloroform on PerkinElmer instrument. The optical rotation of compound 1 was recorded on a Perkin-Elmer 241 polarimeter.

### Fungal cultivation

The isolation, microbial identification, characterization and cultivation for *Trichoderma velutinum* has been described in previous reports.^33,54^

### Isolation of compounds (1–4)

The semi-preparative HPLC purification was performed using reverse-phase (RP) column (Reprosil Gold C_18_, 10 × 250 mm, 5 μm, Dr Maisch GmbH) on ThermoFinnigan HPLC system. The purification details were same as provided in the previous report.33 The Fr-6 on further isolation gives three fractions (Fig. S2, ESI†), one was lipovelutibol A, the other two being velutibol A (1) and velutibol B (2) in 3.5 mg and 0.3 mg respectively. Similarly, Fr-7 gave two fractions, the first being lipovelutibol B, and the second fraction (0.4 mg) was re-purified through-HPLC using RP column (LiChrospher RP18e, 125 × 4 mm, 5 μm, Merck) on Shimadzu HPLC system through a gradient program comprising 0.1% TFA in water as mobile phase A and acetonitrile as mobile phase B. The method used was initiated with 10% B for 5 min then gradually increased to 60% B in next 30 min, kept at 60% B for 10 min, then gradually decreased to 10% B in 5 min and maintained at 10% B for next 10 min, with an overall run time of 60 min at 1 mL min^−1^ flow rate of and observed at 214 nm wavelength. This gave two fractions as velutibol C (3) (0.2 mg approx.) and velutibol D (4) (0.1 mg approx.) (Fig. S3, ESI†).

#### Velutibol A (1)

Colourless powder, [*α*]^25^_D_ (−) 33 (c 0.03, in chloroform); UV(methanol) *λ*_max_ 193 nm; IR (in chloroform): 3366, 2920, 2850, 1658, 1652, 1534, 1414, 1018 cm^−1^; HRESIMS *m*/*z* 1414.9031 [M + H]^+^ (calcd for C_69_H_120_N_15_O_16_, 1414.9032), 1436.8857 [M + Na]^+^ (calcd for C_69_H_119_N_15_O_16_Na, 1436.8851). See Table 1 for ^1^H and ^13^C NMR data.

### HPLC purity analysis

The HPLC analysis was carried out on Shimadzu HPLC system coupled with a PDA detector, thermostat and quaternary pump using a RP column (LiChrospher RP18e, 5 μm, 250 × 4 mm, Merck). The mobile phase A comprises of 0.1% formic acid in water and mobile phase B as acetonitrile. The gradient program was started initially by keeping 10% B for 5 min, gradually increased from 10–60% B in next 30 min, remain at 60% B for next 10 min, followed by gradual decrease from 60–10% B in 5 min and finally remained at 10 for next 13 min with an overall run time of 63 min. It was detected at 214 nm with 1 mL min^−1^ flow rate.

### LCHRMS analysis

The HRMS and tandem mass spectroscopy was carried out on Agilent HRMS 6450 4HD Q-TOF Mass Spectrometer coupled with LC system. The instrument was maintained at capillary voltage 3500 V, source temperature 350 °C, 7.0 L min^−1^ gas flow and spray voltage of 4.5 KV with a resolution of 30 000 were used for HRMS analysis. The tandem mass spectroscopy (MS^*n*^) was performed for the range of 100–2000 amu at fragmentor voltage at 135.0 V. 20 ions per cycle was selected for precursor ion with the minimum intensity of 5000 and collision induced dissociation (CID) at 30 and 40 V.

#### Amino acid configuration determination (Marfey's analysis)

The Marfey's method was used for amino acid configuration determination. The compound 1 (approx 0.2 mg) is hydrolysed in 9 N HCl (0.5 mL) at 110 °C for 18 h. The solution thus obtained was evaporated under vacuum to dryness. It was then dissolved in water (25 μL) and transferred to 2 mL vial. To this, 1 M solution of sodium bicarbonate (10 μL) and 1% solution of Marfey's reagent in acetone (50 μL) was added. The mixture thus obtained was heated at 40 °C under agitation. After 1 h, 2 N HCl (5 μL) was added to quench the reaction. It was dried under stream of air and reconstituted in methanol (0.2 mL) for further analysis. This was analysed by Shimadzu triple quad MS coupled with LC system using a RP column (LiChrospher RP18e, 5 μm, 250 × 4 mm, Merck). The eluent A comprises of 0.1% formic acid in water and eluent B as acetonitrile was used through a gradient program of 10–60% B in 30 min, keeping 60% B for 10 min, reduced gradually to 10% B in 1 min and maintained at 10% B for next 4 min with 0.5 mL min^−1^ flow rate and overall runtime of 45 min. The amino acids (approx. 0.2 mg) was added with water (50 μL), 1 M sodium bicarbonate solution (20 μL) and 1% solution of Marfey's reagent in acetone (100 μL) in separate 2 mL vials and heated at 40 °C. After 1 h, 2 N HCl (10 μL) was added to the reaction and was dried using air stream. Further, it was reconstituted in methanol (1 mL) and analysed using aforementioned method. This was then compared with LCMS chromatogram of derivatized compound 1 to establish amino acid configuration. The compound 2, 3 and 4 were analysed similarly on 20 μg scale to ascertain amino acid configuration. The amino acids ^L^Ile and ^L^*allo*-Ile were differentiated through a chiral LCMS. These were treated with Marfey's reagent as stated earlier and analysed using column (Merck, ChiraDex HR, 4.0 × 250 mm) with gradient programme. The mobile phase consists of A (0.1% formic acid in water) and B (acetonitrile), with the gradient initiated at 5% B and gradually increased to 50% B in 35 min, stayed at 50% B for next 5 min followed by sharp decrease to 5% B in 2 min and stayed at the same for next 5 min making a overall run time of 47 min with a flow rate of 0.5 mL min^−1^. The compounds 2, 3 and 4 previously treated with acid and derivatized with Marfey's reagent were compared with elution of amino acid standards.

### NMR analysis

The 1D and 2D NMR experiments were recorded on a Bruker 400 MHz NMR instrument. The data was recorded in ppm scale using tetramethylsilane or residual NMR solvent resonance as a reference. All the 1D and 2D NMR spectra were processed *via* MestReNova. The NMR-VT experiment was carried out at Bruker 500 MHz spectrometer coupled with a thermostat. The data was acquired at four different temperatures *i.e.* at 298 K, 308 K, 318 K and 328 K.

### CD analysis

The circular dichroism (CD) experiment was performed on a Jasco J-1500 CD spectrophotometer using 2 mm cuvette. The solution of compound 1 in methanol was prepared at 0.1 mg mL^−1^ concentration. The spectrum was recorded from 260 to 180 nm with 50 nm min^−1^ scanning speed at a response time of 1 s and 1 nm bandwidth. The data obtained was accumulation of two scans.

### Cytotoxic assay (MTT-assay)

The cytotoxic effect of compound 1 on cancer cells was assayed by using MTT dye. The HL-60 cell lines were seeded for 4 h in round bottom 96 well plates. It was then treated with different concentrations of compound 1 dissolved in DMSO and incubated for 48 h. With 3 h earlier to experiment termination, the MTT dye (20 μL) from the stock solution of 2.5 mg mL^−1^ was added to each well. The MTT-formazan crystals thus formed were dissolved in DMSO (160 μL) and observed at 570 nm. The untreated control culture was treated with DMSO (<0.2%).

### Cell cycle analysis

The cell cycle phase distribution analysis in HL-60 cells was carried out by propidium iodide (PI) staining. The HL-60 cells were treated with different concentration (5, 10, 20 and 30 μM) of 1. After 24 h, the cells were centrifuged and collected at 400 × *g* and washed two times with PBS followed by fixing with ice-cold ethanol (70%). It was incubated overnight at 4 °C. Next day, cells were centrifuged at 4000 rpm, collected and washed with PBS followed by incubation with RNase (0.2 mg mL^−1^) for 90 min at 37 °C. It was then stained with PI (10 μg mL^−1^) and kept for 30 min in dark. The analysis of cells were carried out through flow-cytometer (FACS Calibur, Becton Dickinson) using list mode on 10 000 events for FL2-A *vs.* FL2-W. The ModFit software was used for the calculation of fluorescence intensity of the sub-G1 cell fraction representing the apoptotic cell population.

### Hoechst staining

HL-60 cells were incubated with compound 1 in a 12 well plate. After 24 h, cells were centrifuged at 400 × *g*, collected and washed two times with PBS. The cells were fixed using 1 mL solution of cold acetic acid in methanol (1 : 3) and incubated overnight at 4 °C. Further, the cells were centrifuged at 400 × *g*, collected and re-suspended in fixing solution (100 μL). Further, the cells were spread on a slide and dried for 4–6 h at room temperature. Slides were then stained with DAPI in PBS containing 0.05% Tween 20 (5 μg mL^−1^) and incubated for 30 min followed by washing with water and PBS. The slide covered by a coverslip was mounted with fluid containing 50 μL glycerol and PBS (1 : 1) and sealed with white nail polish. Nuclei were observed using microscope (20×) and photographed by Olympus IX70 microscope. The treated and untreated control cells were simply photographed using a microscope.

### Anti-tubercular assay

The MIC of compound 1 was determined by the micro-broth dilution method against *M. tuberculosis* H_37_Rv.^55^ The bacterial cells was grown in Middlebrook 7H9 broth containing glycerol (0.5%), Tween 80 (0.05%), and ADC (10%) under constant shaking for 10 to 15 days at 37 °C in 5% CO_2_. A suspension of *M. tuberculosis* cells was prepared in normal saline solution containing Tween 80 (0.5%). The turbidity of the resulting suspension was attuned by 1 McFarland (McF) standard (≈1.0 × 10^7^ CFU mL^−1^). The bacterial suspension was further diluted 10 folds with the medium. The compound 1 was serially diluted in Middlebrook 7H9 to 2-folds in 100 μL per well in 96-well U bottom microtitre plates. A 100 μL of the diluted suspension was added to each well resulting in a final inoculum of 1.0 × 10^6^ CFU mL^−1^ in the well, and the compound 1 was finally in range from 0.5 μg mL^−1^ to 128 μg mL^−1^. The plates were incubated at 37 °C for seven days in 5% CO_2_. The Resazurin Microtiter Assay (REMA) method was used for evaluation of the results. After incubation, resazurin (15 μL, 0.04%) and Tween 80 (12.5 μL, 20%) was added in each well of the plate. The plates were incubated for 48 h and were read visually to ascertain the MIC from the minimum concentration of the compound showing no change in color.

## Conclusions

Herein, we report isolation of four 14-residue peptaibols from a psychrotrophic fungus *T. velutinum* of the Himalayan cold habitat. The complete structural characterization of 1 was established by intensive 1D, 2D NMR, and tandem mass spectroscopic studies. Whereas, the probable primary sequence for compound 2–4 was established using MS/MS studies. The stereochemical determination by Marfey's analysis revealed l-form of the amino acids in these putative peptides. The conformational studies carried out using CD spectroscopy revealed that compound 1 folds in a 3_10_-helix in methanol. The variable temperature experiment performed through NMR showed that NH-3 (Leu-3), NH-4 (Aib-4), NH-6 (Leu-7), NH-7 (Aib-8), NH-9 (Aib-10), NH-10 (Aib-11), NH-11 (Aib-12) and NH-12 (Leuol) were involved in intramolecular hydrogen bonding in peptaibol 1. These experiments also gave an insight into its secondary structure and may correlate to the biological activities asserted by 1. The cytotoxic assay showed compound 1 having IC_50_ values of 7 μM and 4 μM against MDA-MD-231 and HL-60 cancer cells respectively. Further, the cell cycle assay revealed DNA damage and formation of apoptotic bodies in a dose-dependent manner against HL-60 cancer cells. The compound 1 also showed weak MIC of 32 μg mL^−1^ against *M. tuberculosis*.

## Conflicts of interest

There are no conflicts to declare.

## Supplementary Material

RA-010-D0RA05780K-s001
