# Supplementary material for: 14-Residue peptaibol velutibol A from Trichoderma velutinum: its structural and cytotoxic evaluation
Source: RSC Adv. 2020 Aug 24;10(52):31233–42. doi: 10.1039/d0ra05780k (PMC9056410; doi:10.1039/d0ra05780k)
Supplement: RA-010-D0RA05780K-s001 [file RA-010-D0RA05780K-s001.pdf]

Electronic Supplementary Material (ESI) for RSC Advances.
This journal is © The Royal Society of Chemistry 2020

Supporting Information

14-Residue Peptaibol Velutibol A from *Trichoderma velutinum* of the Himalayan Cold Habitat with Cytotoxic and Anti-tubercular Activity.

Varun Pratap Singh,[a,b] *Anup Singh Pathania,*[c] *Manoj Kushwaha,*[d] *Samsher Singh,*[e] *Vandana Sharma,*[d,g] *Fayaz Malik,*[c] *Inshad Khan,*[e,f] *Anil Kumar,*[b] *Deepika Singh,*[*,a,d] *and Ram A. Vishwakarma*[*,a]

[a] Medicinal Chemistry Division, CSIR-Indian Institute of Integrative Medicine, Canal Road, Jammu 180 001, India

[b] Department of Biotechnology, Faculty of Sciences, Shri Mata Vaishno Devi University, Katra, Jammu and Kashmir 182320, India.

[c] Pharmacology Division, CSIR-Indian Institute of Integrative Medicine, Canal Road, Jammu 180 001, India

[d] Quality Control and Quality Assurance Division, CSIR-Indian Institute of Integrative Medicine, Canal Road, Jammu 180 001, India

[e] Clinical Microbiology Division, CSIR-Indian Institute of Integrative Medicine, Canal Road, Jammu 180 001, India

[f] Department of Microbiology, Central University, Rajasthan 305 817, India

[g] Academy of Scientific and Innovative Research, Jammu 180001, India

RTable of content

S. No.	Contents	Pages
1	**Fig. S1**: HPLC chromatogram of crude extract showing for 15 fractions	4
2	**Fig. S2**: HPLC chromatogram for purification of compound **1** and **2**	4
3	**Fig. S3**: HPLC chromatogram for re-purification for compound **3** and **4.**	5
4	**Fig. S4:** HRMS of compound **1**.	6
5	**Fig. S5:** ^{1}H NMR of compound **1** in DMSO-d_6 at 400 MHz	7
6	**Fig. S5a:** Expanded ^{1}H NMR of compound **1** in DMSO-d_6 at 400 MHz (6.8 – 9.0 ppm)	7
7	**Fig. S5b:** Expanded ^{1}H NMR of compound **1** in DMSO-d_6 at 400 MHz (3.0 – 4.4 ppm)	8
8	**Fig. S5c:** Expanded ^{1}H NMR of compound **1** in DMSO-d_6 at 400 MHz (0.0 – 2.6 ppm)	8
9	**Fig. S6:** ^{13}C NMR of compound **1** in DMSO-d_6 at 100 MHz	9
10	**Fig. S6a:** Expanded ^{13}C NMR of compound **1** in DMSO-d_6 at 100 MHz	9
11	**Fig. S6b:** Expanded ^{13}C NMR of compound **1** in DMSO-d_6 at 100 MHz	10
12	**Fig. S6c:** Expanded ^{13}C NMR of compound **1** in DMSO-d_6 at 100 MHz	10
13	**Fig. S7:** DEPT-135 NMR of compound **1** in DMSO-d_6 at 100 MHz	11
14	**Fig. S7a:** Expanded DEPT-135 NMR of compound **1** in DMSO-d_6 at 100 MHz	11
15	**Fig. S8:** DEPT-90 NMR of compound **1** in DMSO-d_6 at 100 MHz	12
16	**Fig. S9:** COSY spectrum of compound **1** in DMSO-d_6 at 400 MHz for ^{1}H NMR	13
17	**Fig. S9a:** Expanded COSY spectrum of compound **1** in DMSO-d_6 at 400 MHz for ^{1}H NMR	14
18	**Fig. S9b:** Expanded COSY spectrum of compound **1** in DMSO-d_6 at 400 MHz for ^{1}H NMR	15
19	**Fig. S10:** TOCSY spectrum of compound **1** in DMSO-d_6 at 400 MHz for ^{1}H NMR	16
20	**Fig. S10a:** Expanded TOCSY spectrum of compound **1** in DMSO-d_6 at 400 MHz for ^{1}H NMR	17
21	**Fig. S11:** NOESY spectrum of compound **1** in DMSO-d_6 at 400 MHz for ^{1}H NMR	18
22	**Fig. S11a:** Expanded NOESY spectrum of compound **1** in DMSO-d_6 at 400 MHz for ^{1}H NMR	19
23	**Fig. S11b:** Expanded NOESY spectrum of compound **1** in DMSO-d_6 at 400 MHz for ^{1}H NMR	20
24	**Fig. S12:** HMBC spectrum of compound **1** in DMSO-d_6 at 400 MHz for ^{1}H NMR	21
25	**Fig. S12a:** Expanded HMBC spectrum of compound **1** in DMSO-d_6 at 400 MHz for ^{1}H NMR	22
26	**Fig. S12b:** Expanded HMBC spectrum of compound **1** in DMSO-d_6 at 400 MHz for ^{1}H NMR	23
27	**Fig. S13:** HSQC spectrum of compound **1** in DMSO-d_6 at 400 MHz for ^{1}H NMR and using DEPT 135 for f1.	24
28	**Table S1**: 2D-COSY, TOCSY and NOESY correlations of **1** at 400MHz for ^{1}H NMR.	25
29	**Table S2**: 2D-HMBC correlations of **1** at 400 MHz for ^{1}H NMR and 100 MHz for ^{13}C NMR	27
30	**Fig. S14**: Marfey's analysis of **1** using LCMS	29
31	**Fig. S15**: HPLC purity of compound **1**.	30
32	**Fig. S16:** UV-spectrum of compound **1**.	31
33	**Fig. S17:** IR spectrum of compound **1** in $CHCl_3$	32
34	**Fig. S18**: HPLC chromatogram of **2**	33
35	**Fig. S19:** HRMS of **2**	34
36	**Fig. S20a**: MS/MS of compound **2** for *m/z* 1414.9030 $[M+H]^+$	35
37	**Fig. S20b**: MS/MS of *m/z* 454.2661 daughter ion b_4 for compound **2.**	36
38	**Fig. S21**: HPLC chromatogram of **3**	37
39	**Fig. S22:** HRMS of **3.**	38
40	**Fig. S23a**: MS/MS of compound **3** for *m/z* 1414.9043 $[M + H]^+$.	39
41	**Fig. S23b**: MS/MS of *m/z* 1200.7363 daughter ion b_{12} for compound **3.**	40
42	**Fig. S23c**: MS/MS of *m/z* 848.5256 daughter ion b_8 for compound **3.**	41
43	**Fig. S24:** HPLC chromatogram of compound **4.**	42
44	**Fig. S25**: HRMS of **4.**	43
45	**Fig. S26a:** MS/MS of *m/z* 1428.9191 parent ion $[M+H]^+$ for compound **4**.	44
46	**Fig. S26b:** MS/MS of *m/z* 1214.7532 daughter ion b_{12} for compound **4.**	45
47	**Fig. S27:** HPLC overlay chromatogram of compounds **1**, **2**, **3** and **4.**	46
48	**Fig. S28**: Marfey's analysis of compound **2**	47

49	**Fig. S29**: Extracted ion chromatograms of *m/z* 368, 370, 384 and 400 for compound **2**	48
50	**Fig. S30**: Marfey's analysis of compound **3**	49
51	**Fig. S31**: Extracted ion chromatograms of *m/z* 368, 370, 384 and 400 for compound **3**	50
52	**Fig. S32**: Marfey's analysis of compound **4**	51
53	**Fig. S33**: Extracted ion chromatograms of *m/z* 368, 370, 384 and 400 for compound **4**	52
54	**Fig. S34**: Extracted ion chromatograms of *m/z* 382 (-ESI) for compound ^{L}*allo*-Ile, ^{L}Ile **2**, **3** and **4** using chiral LCMS	53
55	**Fig. S35:** NMR-VT experiment performed at 298K, 308K, 318K, and 328K for compound **1** in DMSO-d_6 at 400 MHz (Region δ 6.7 – 9.1).	54
56	**Fig. S36:** Anti-tubercular screening report of compound **1**	55

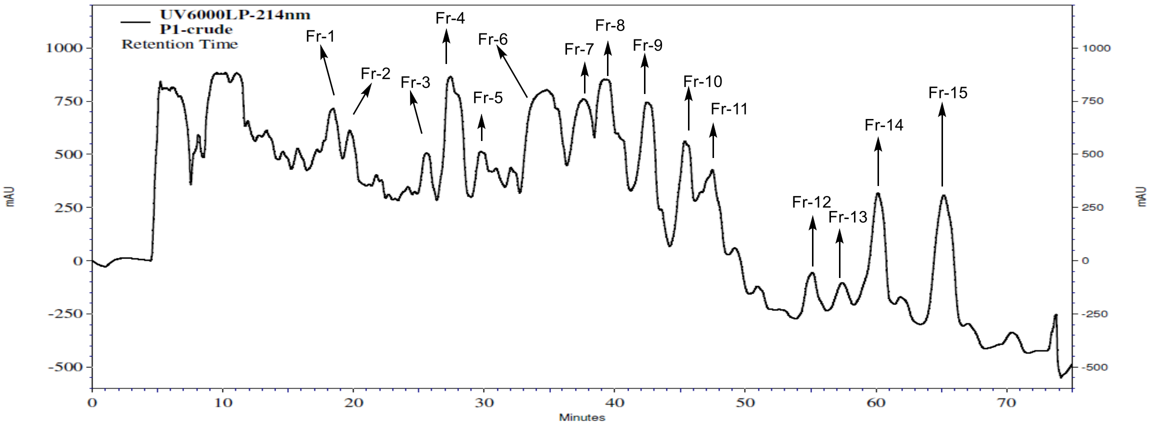

Fig. S1: HPLC chromatogram of crude extract showing for 15 fractions

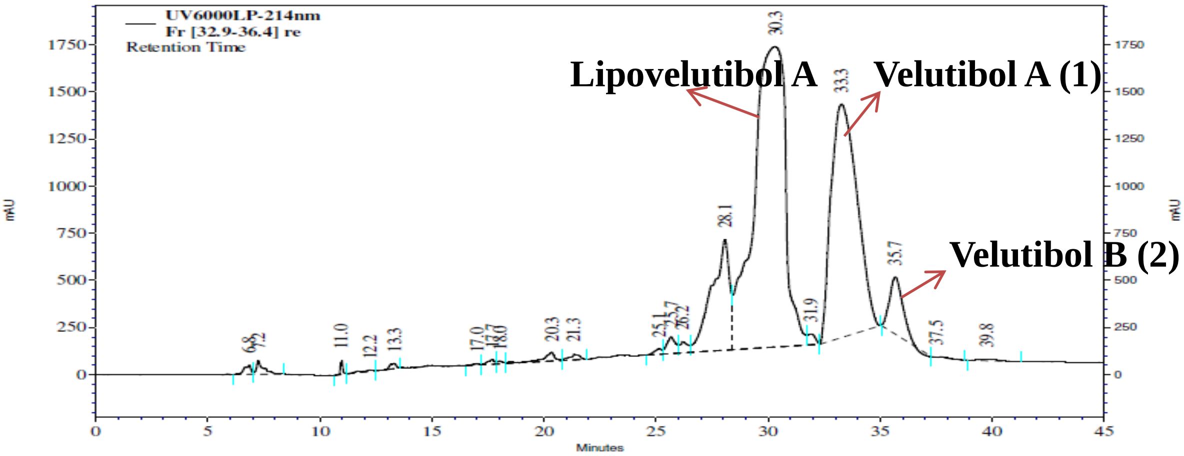

Fig. S2: HPLC chromatogram for purification of compound **1** and **2.**

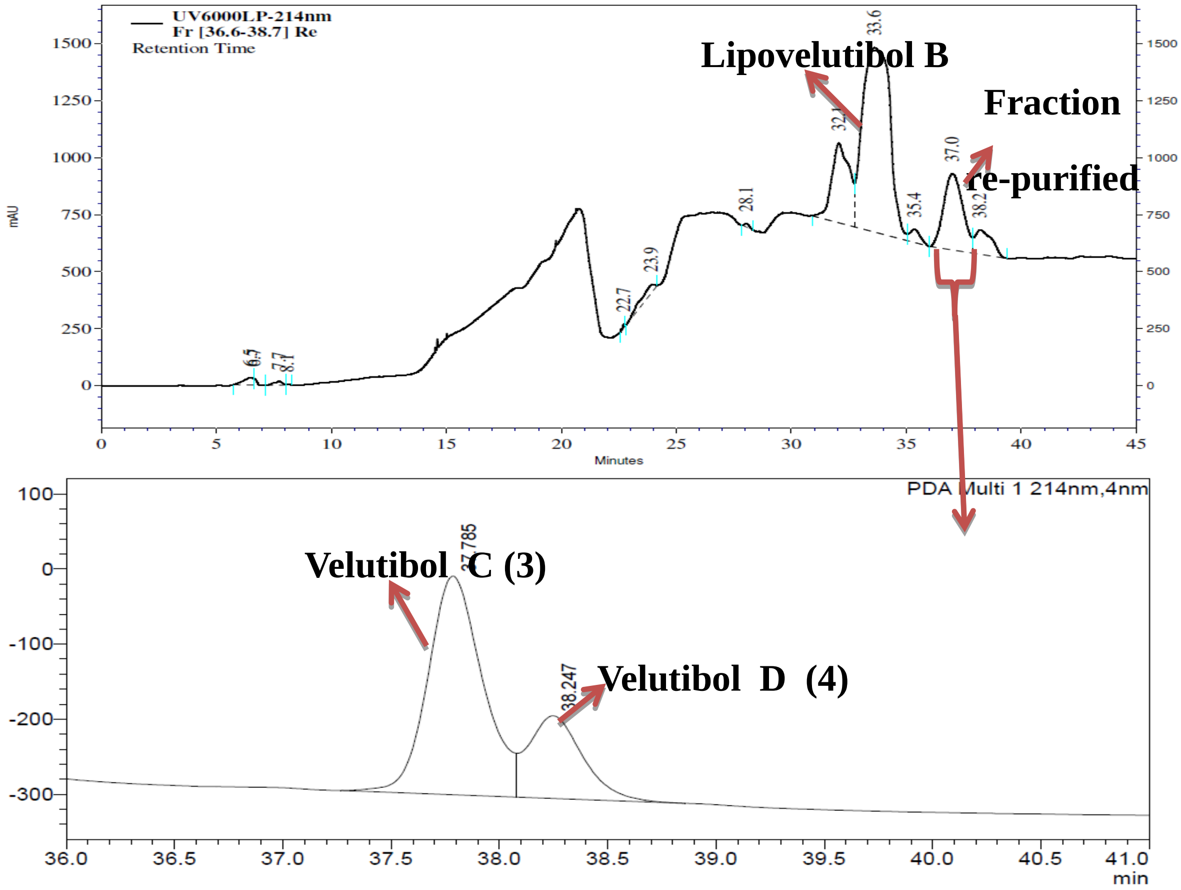

Fig. S3: HPLC chromatogram for re-purification for compound **3** and **4.**

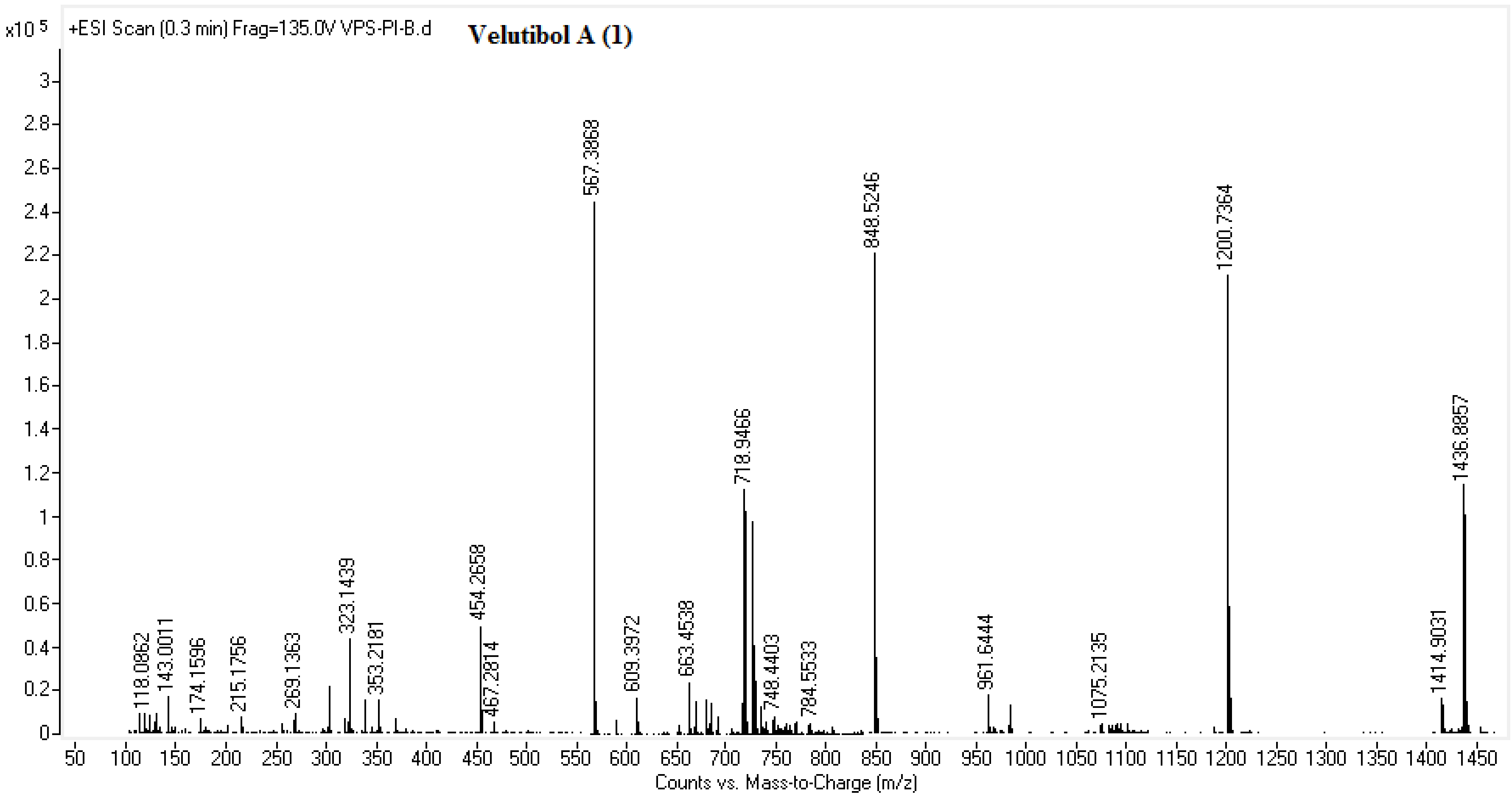

Fig. S4: HRMS of compound **1**.

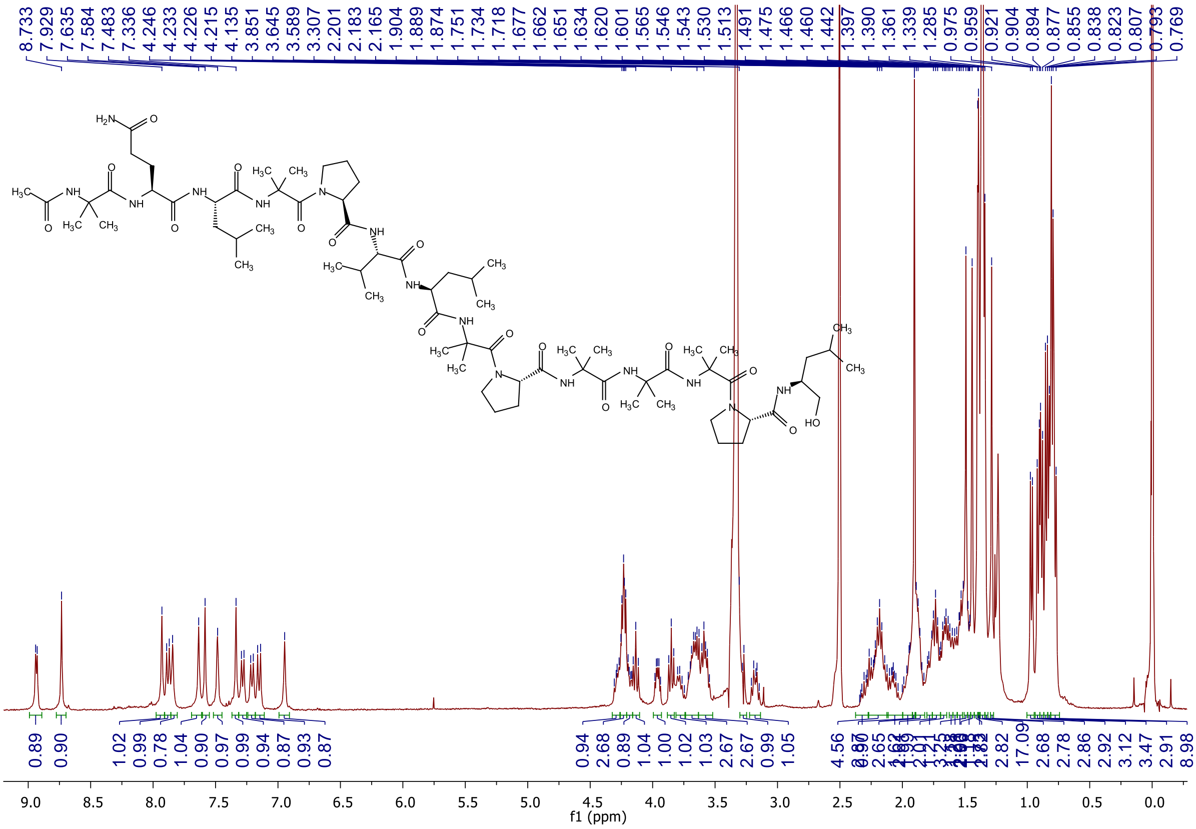

Fig. S5: ^{1}H NMR of compound **1** in DMSO-d_6 at 400 MHz

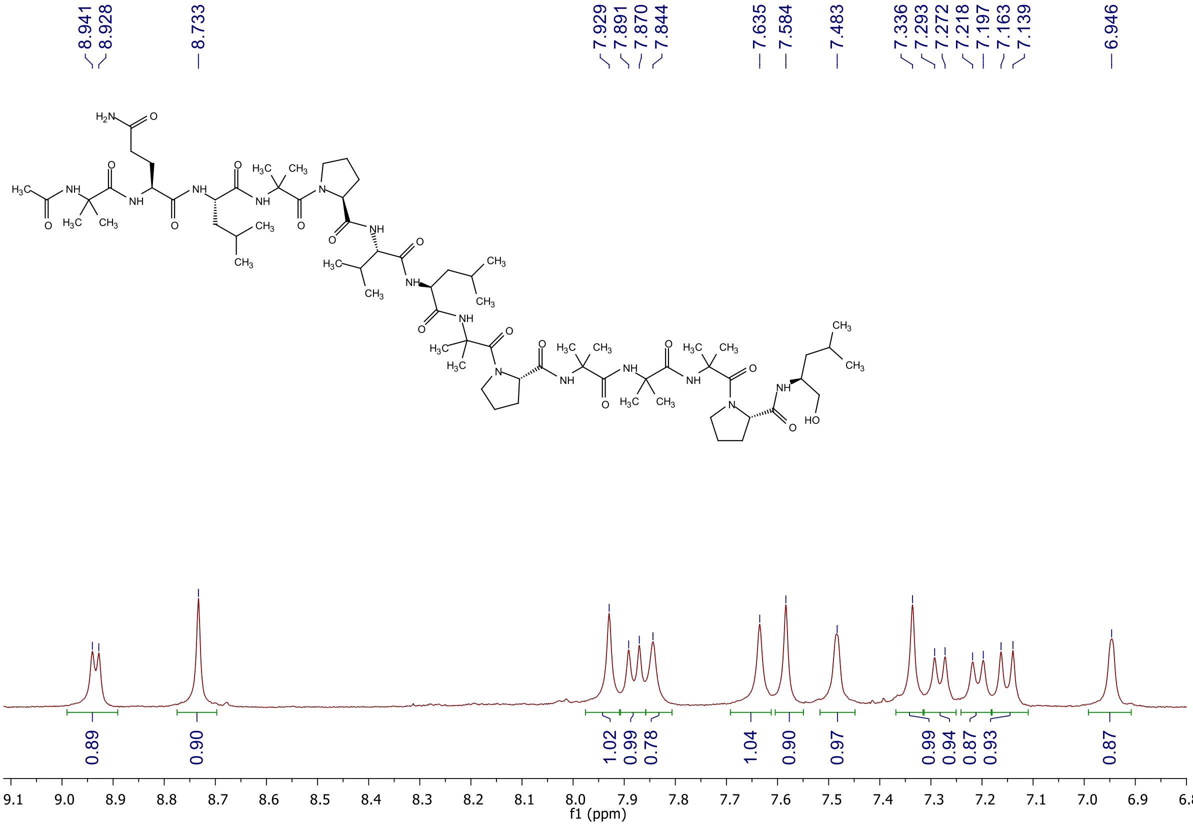

Fig. S5a: Expanded ^{1}H NMR of compound **1** in DMSO-d_6 at 400 MHz (6.8 – 9.0 ppm)

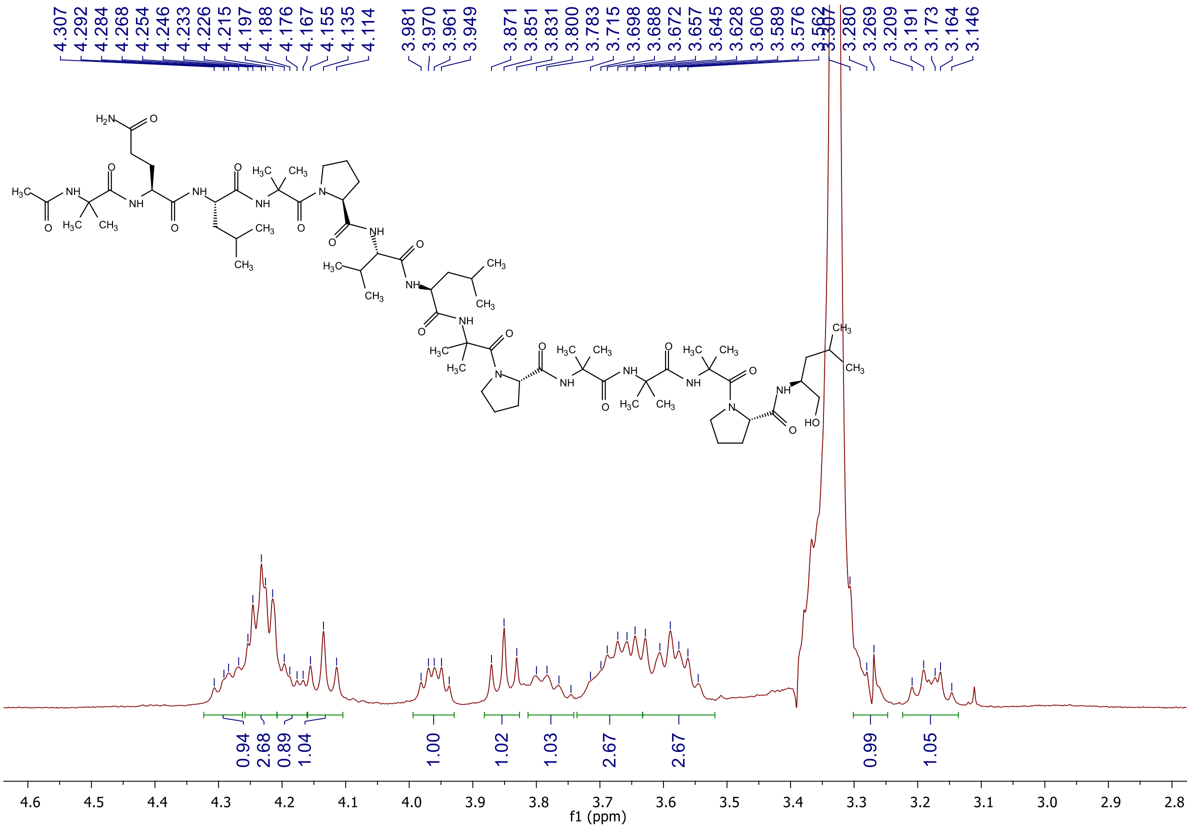

Fig. S5b: Expanded ^{1}H NMR of compound **1** in DMSO-d_6 at 400 MHz (3.0 – 4.4 ppm)

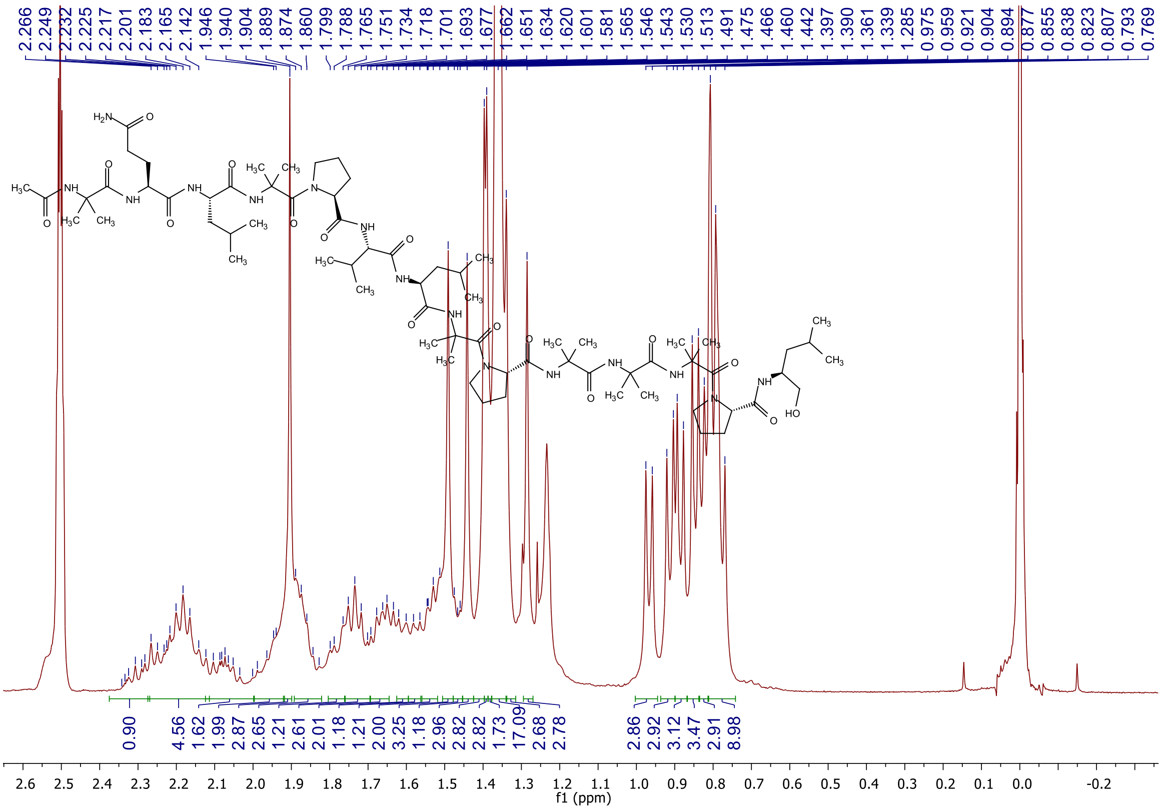

Fi

g. S5c: Expanded ^{1}H NMR of compound **1** in DMSO-d_6 at 400 MHz (0.0 – 2.6 ppm)

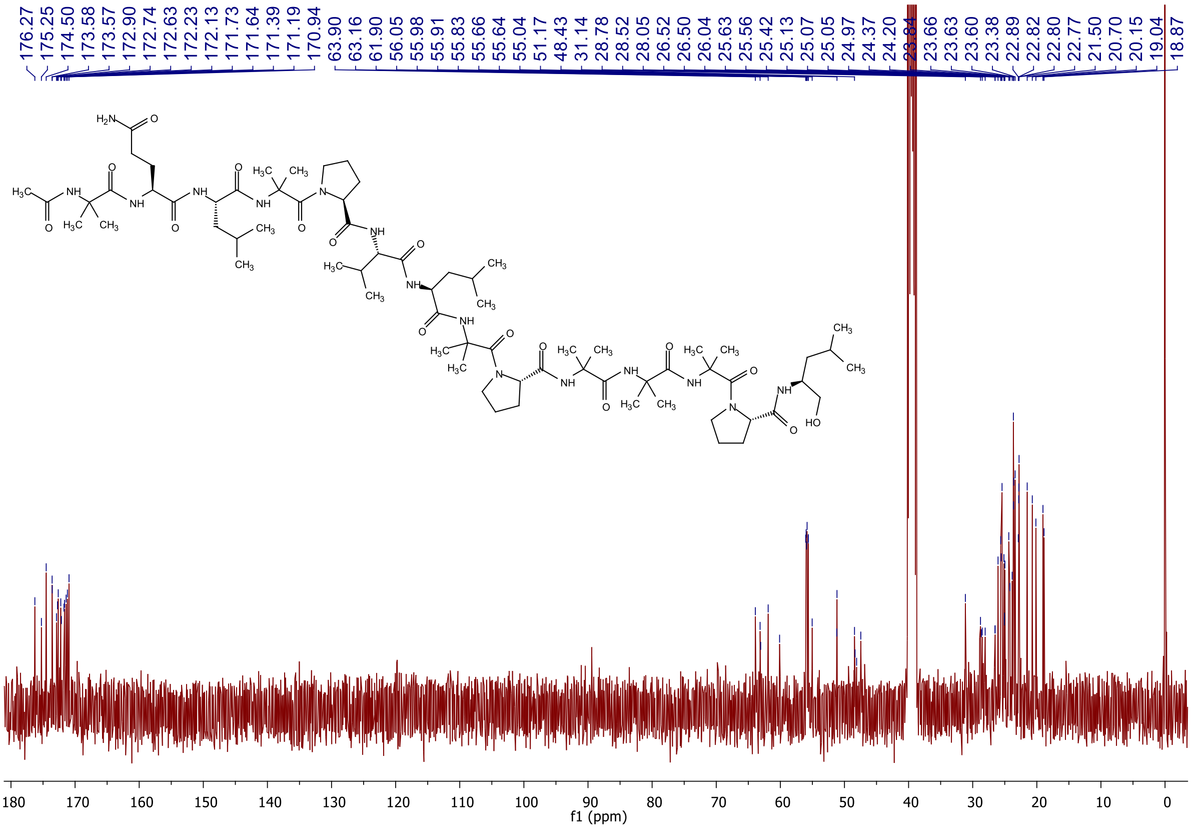

Fig. S6: ^{13}C NMR of compound **1** in DMSO-d_6 at 100 MHz

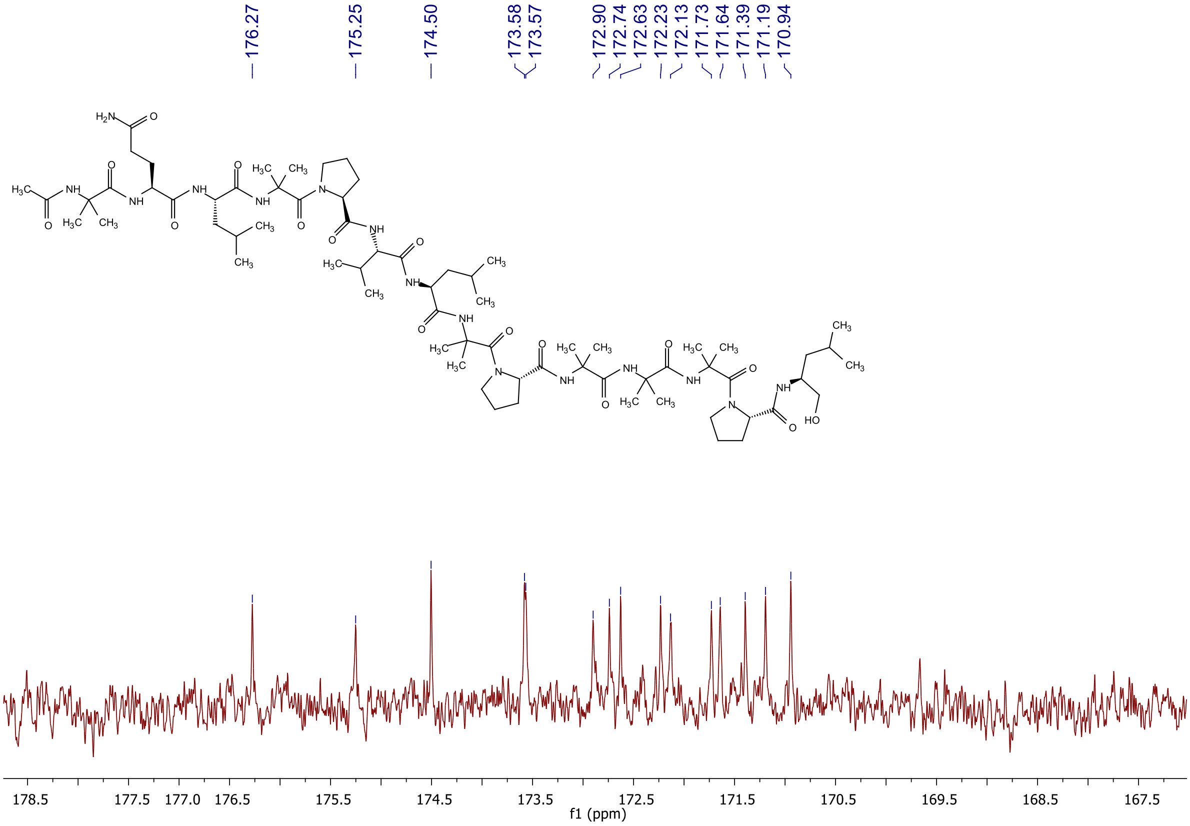

Fig. S6a: Expanded ^{13}C NMR of compound **1** in DMSO-d_6 at 100 MHz

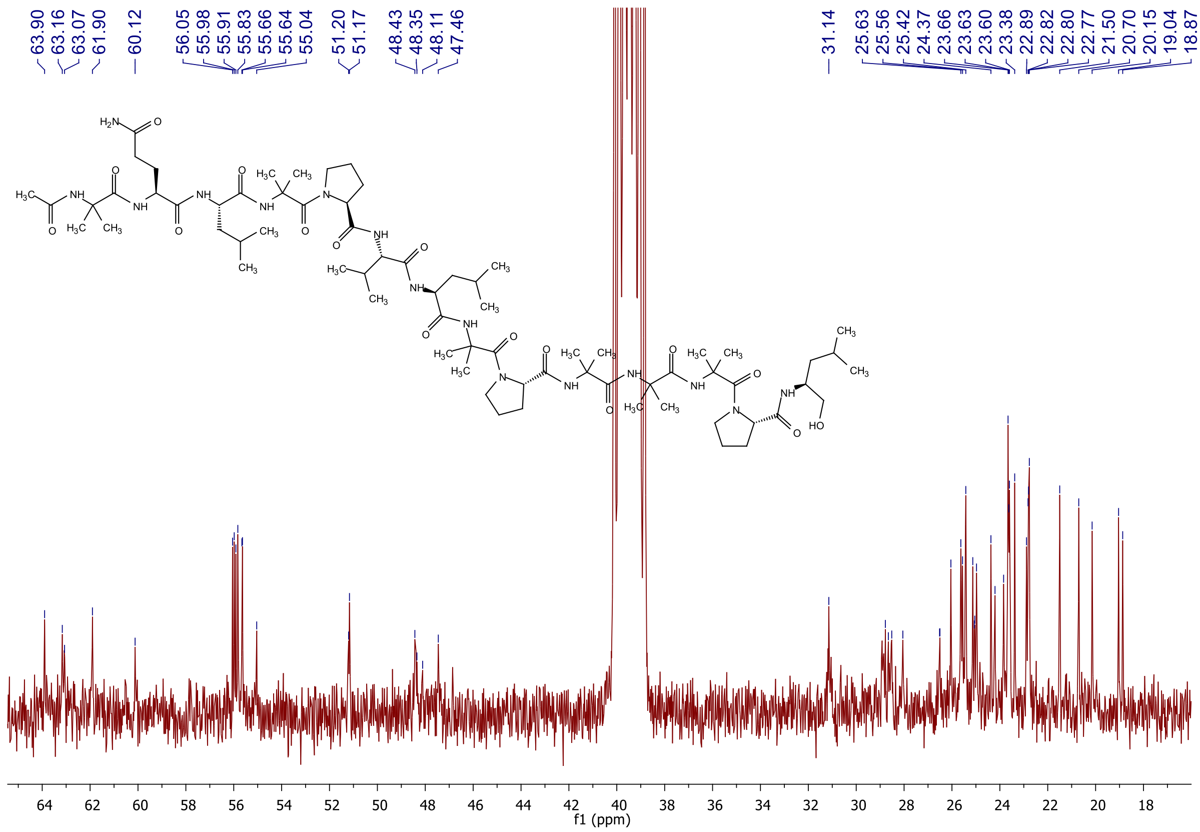

Fig. S6b: Expanded ^{13}C NMR of compound **1** in DMSO-d_6 at 100 MHz

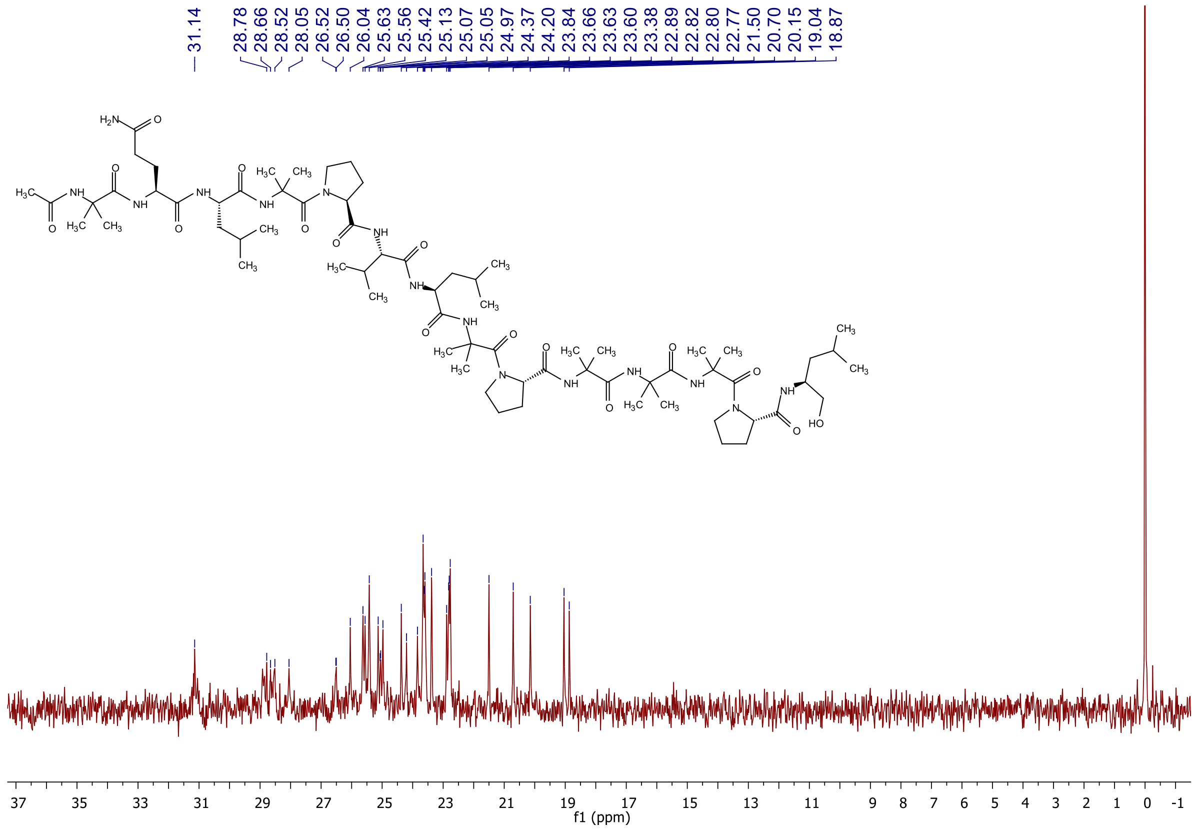

Fig. S6c: Expanded ^{13}C NMR of compound **1** in DMSO-d_6 at 100 MHz

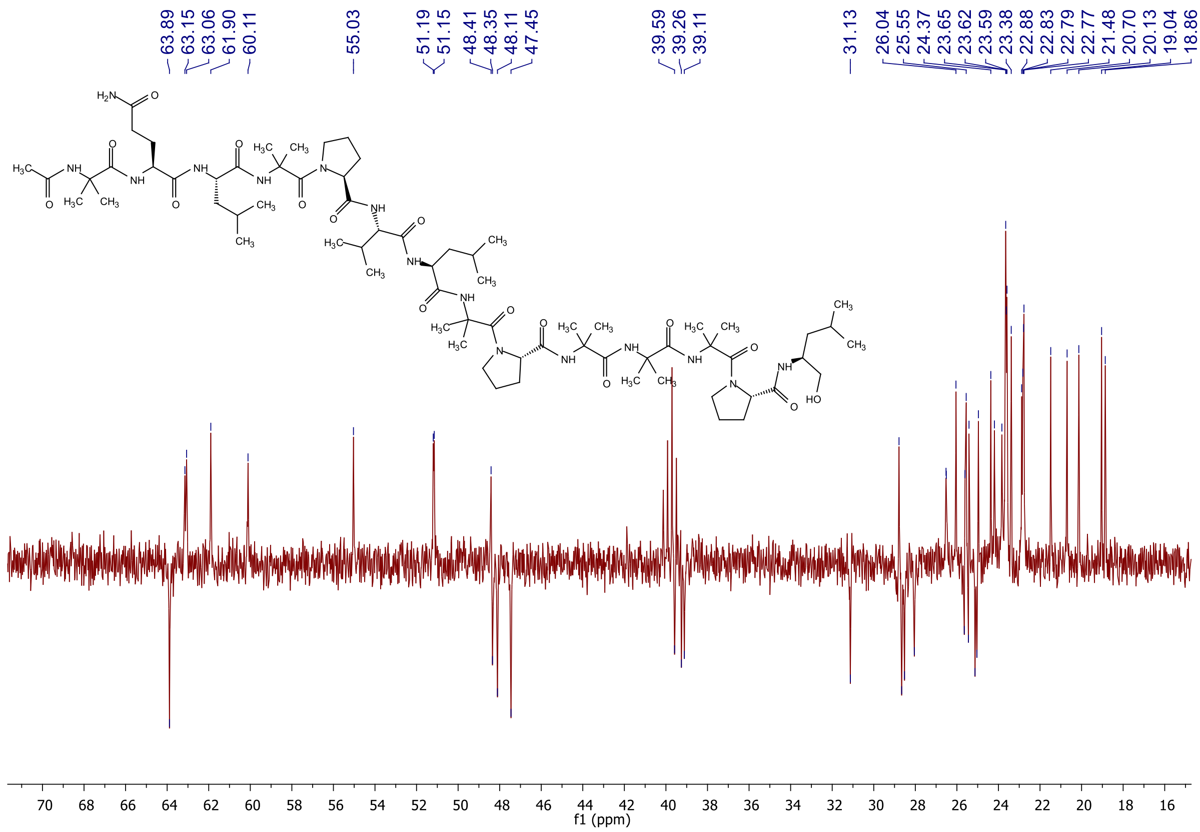

Fig. S7: DEPT-135 NMR of compound **1** in DMSO-d_6 at 100 MHz

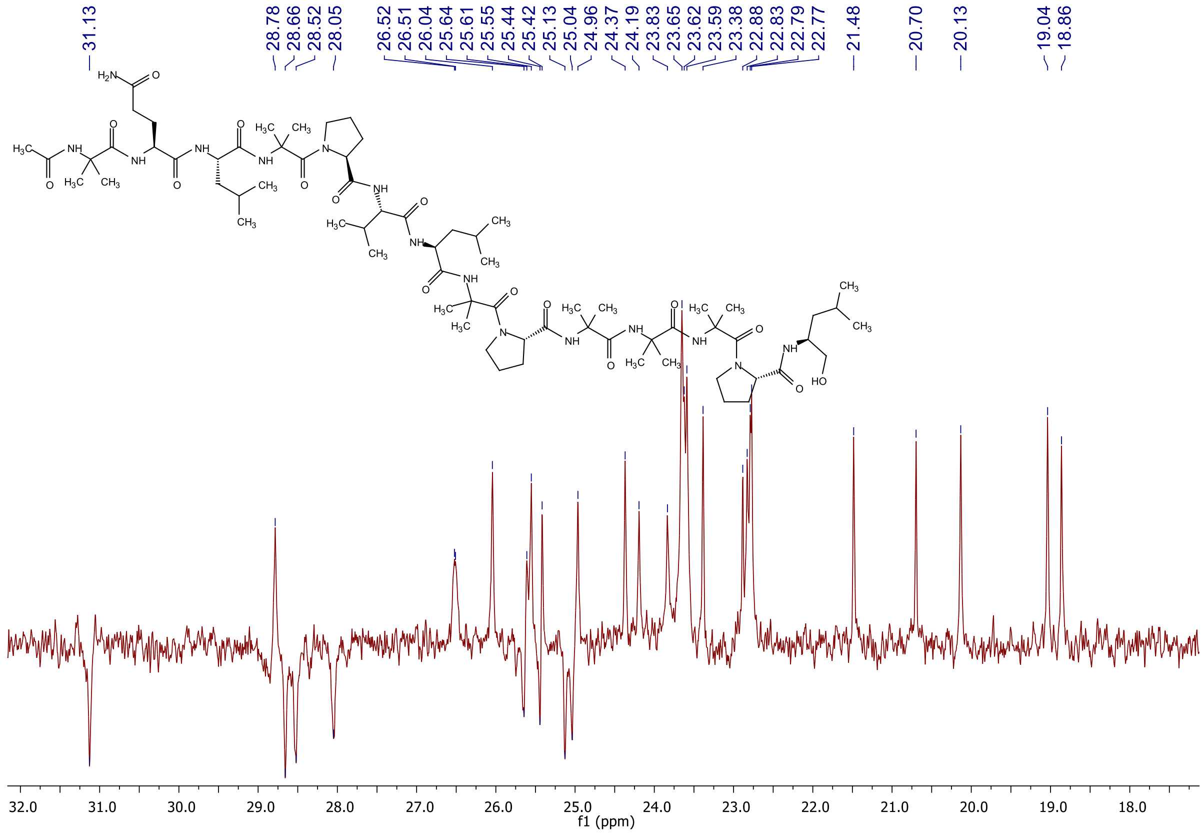

Fig. S7a: Expanded DEPT-135 NMR of compound **1** in DMSO-d_6 at 100 MHz

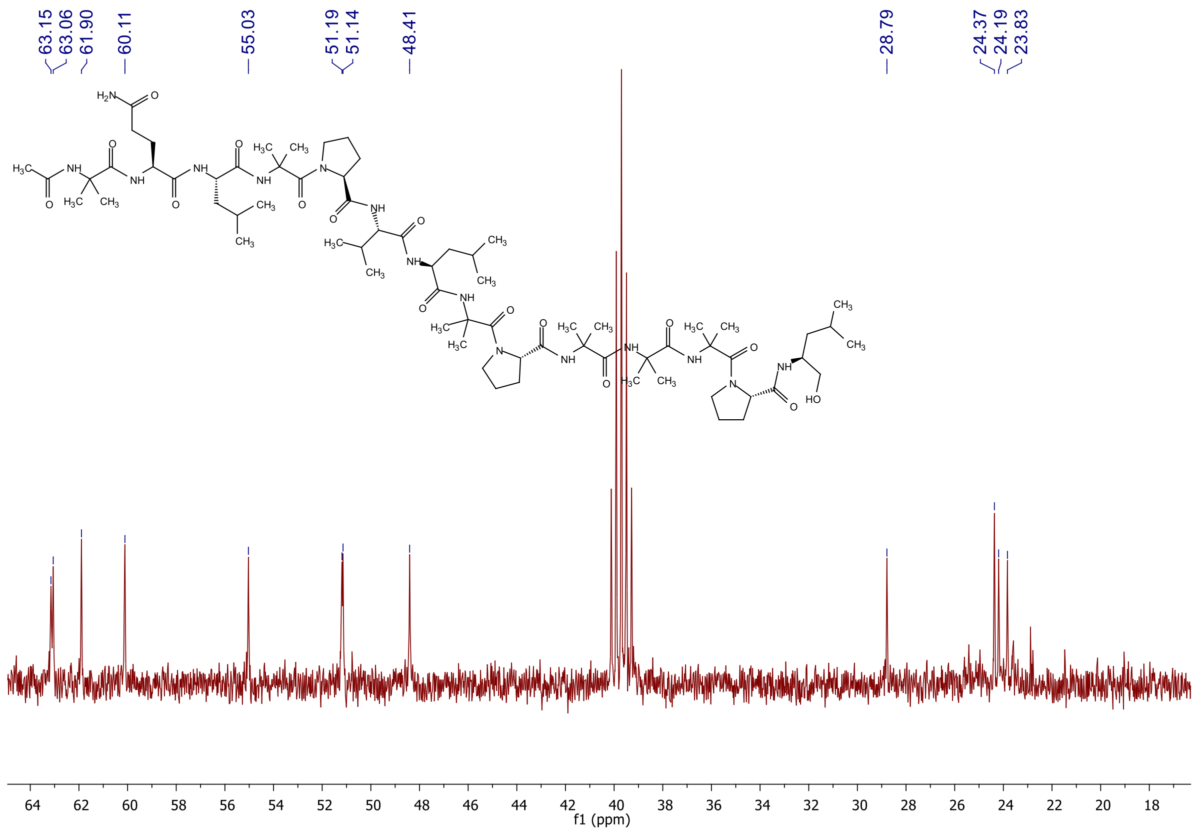

Fig. S8: DEPT-90 NMR of compound **1** in DMSO-d_6 at 100 MHz

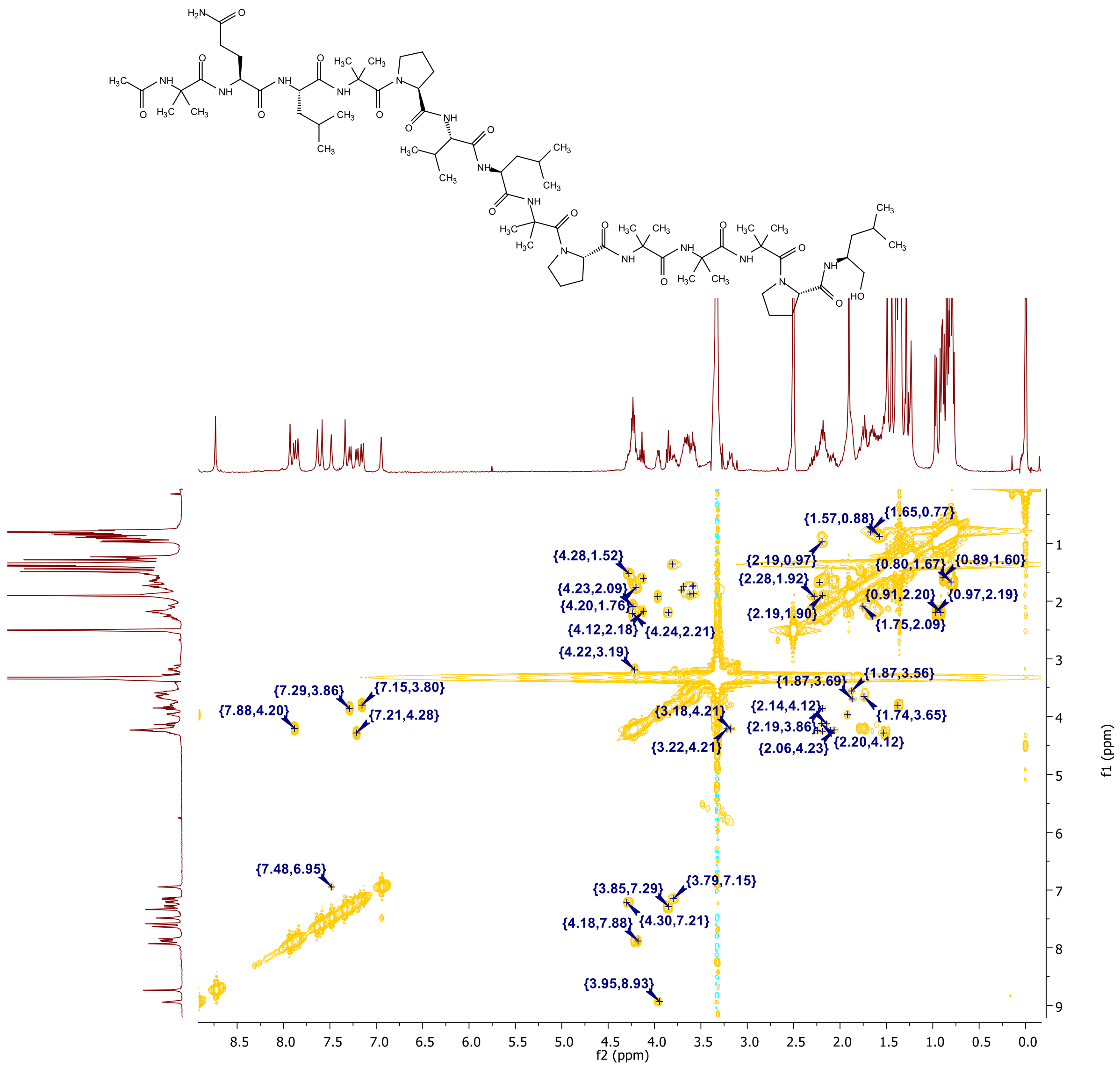

Fig. S9: COSY spectrum of compound **1** in DMSO-d_6 at 400 MHz for ^{1}H NMR

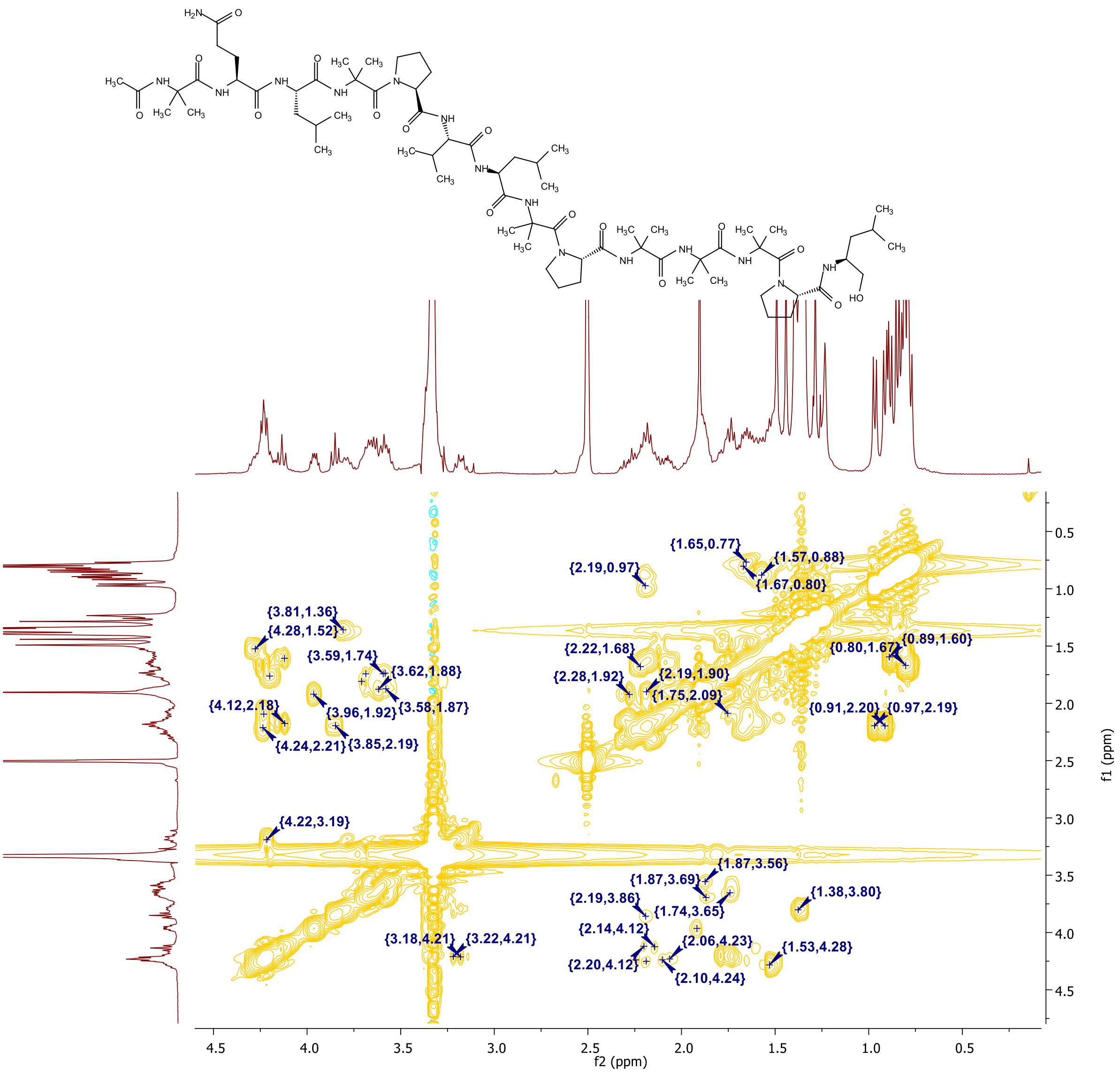

Fig. S9a: Expanded COSY spectrum of compound **1** in DMSO-d_6 at 400 MHz for ^{1}H NMR

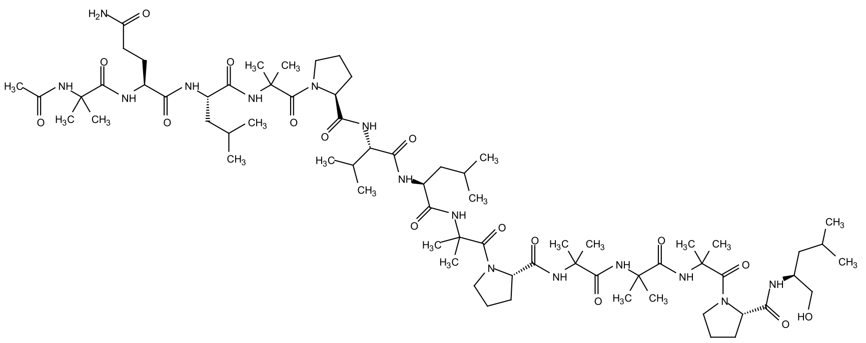

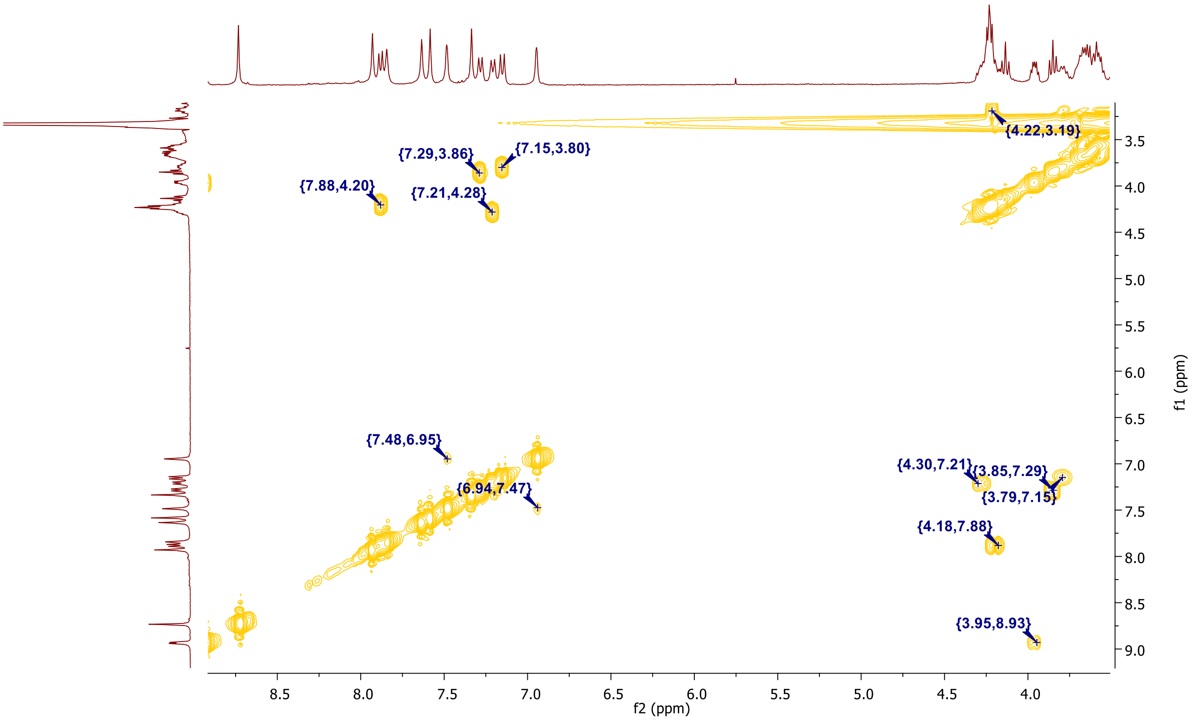

Fig. S9b: Expanded COSY spectrum of compound **1** in DMSO-d_6 at 400 MHz for ^{1}H NMR

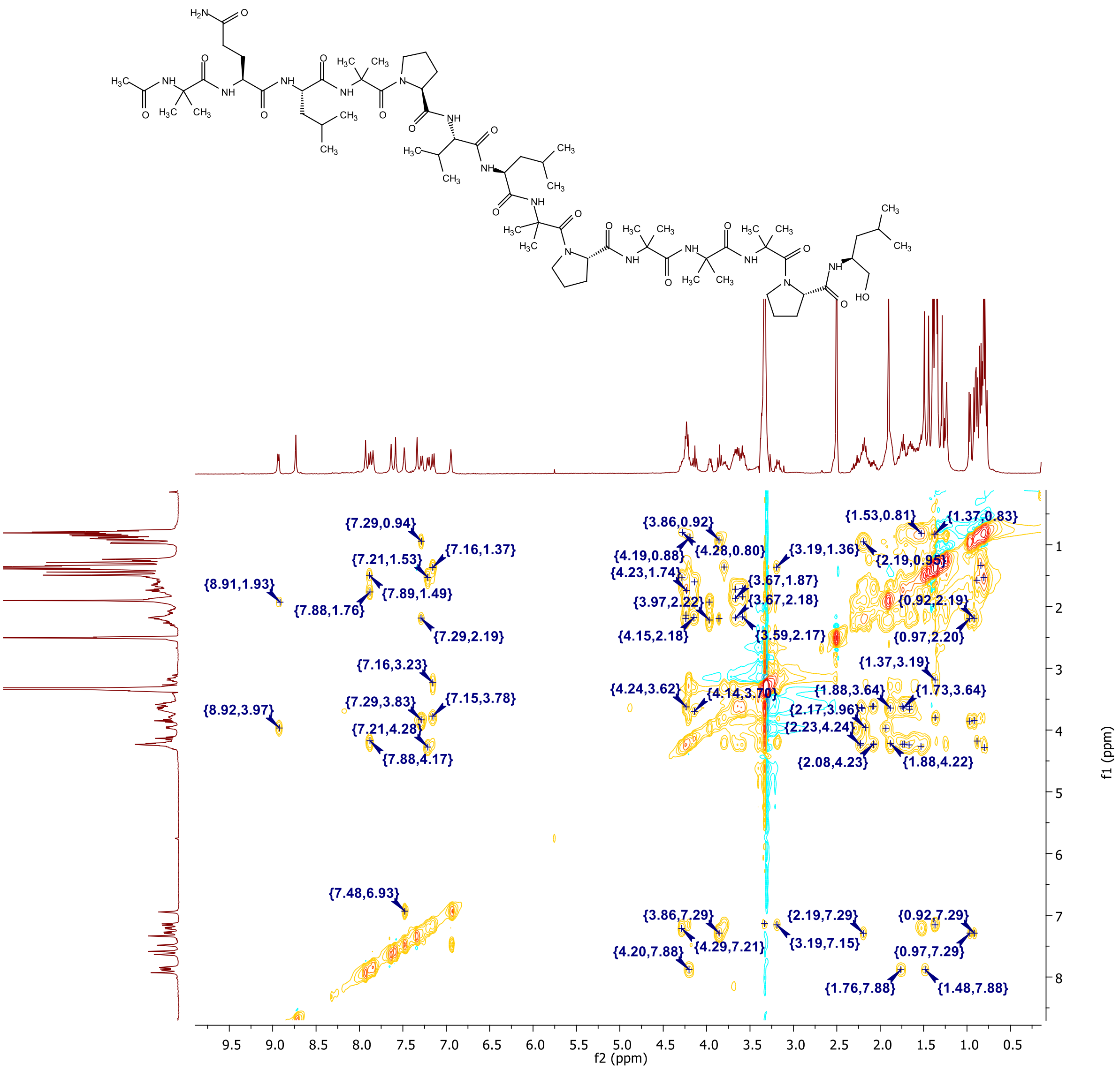

Fig. S10: TOCSY spectrum of compound **1** in DMSO-d_6 at 400 MHz for ^1H NMR

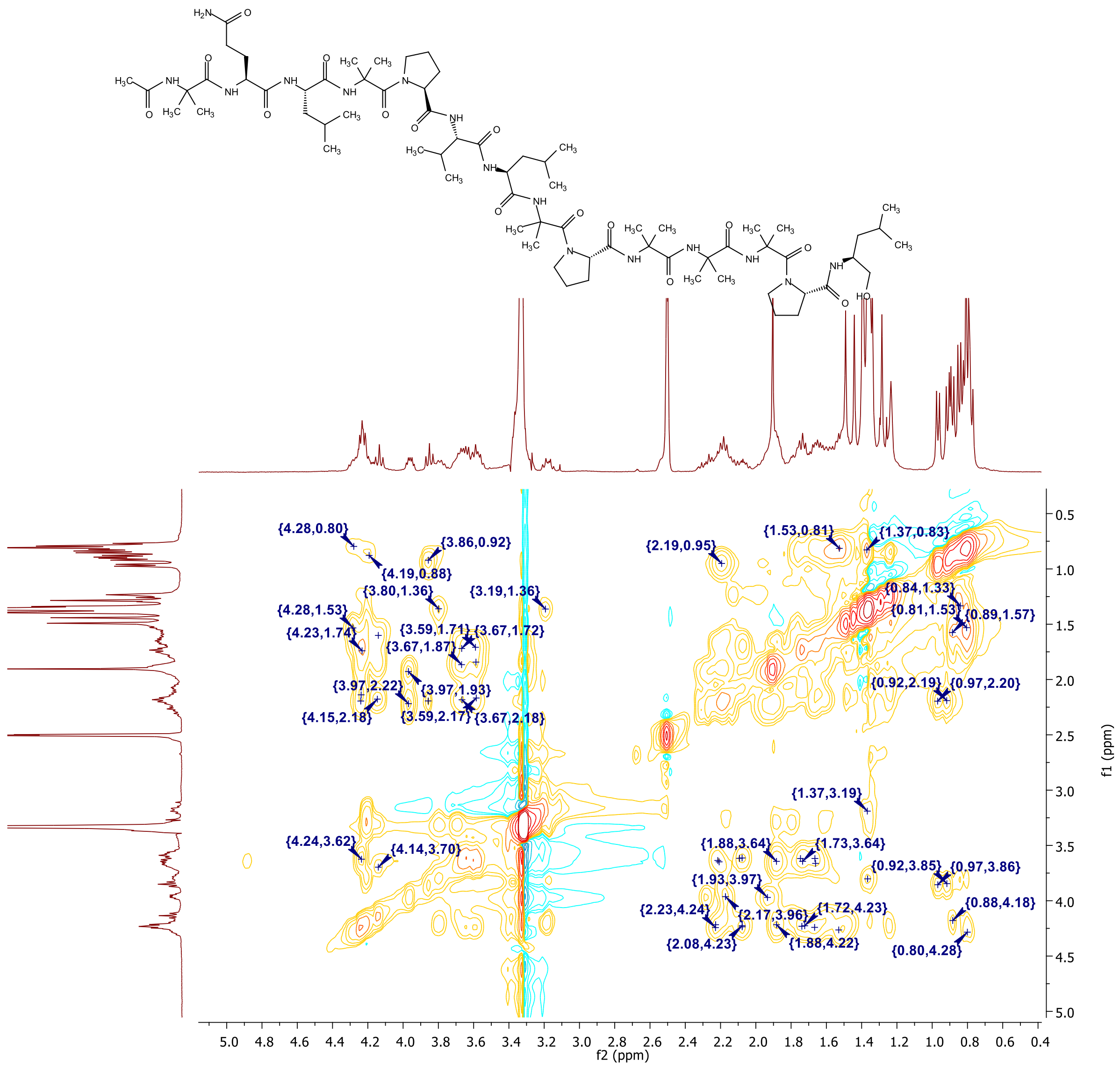

Fig. S10a: Expanded TOCSY spectrum of compound **1** in DMSO-d_6 at 400 MHz for ^1H NMR

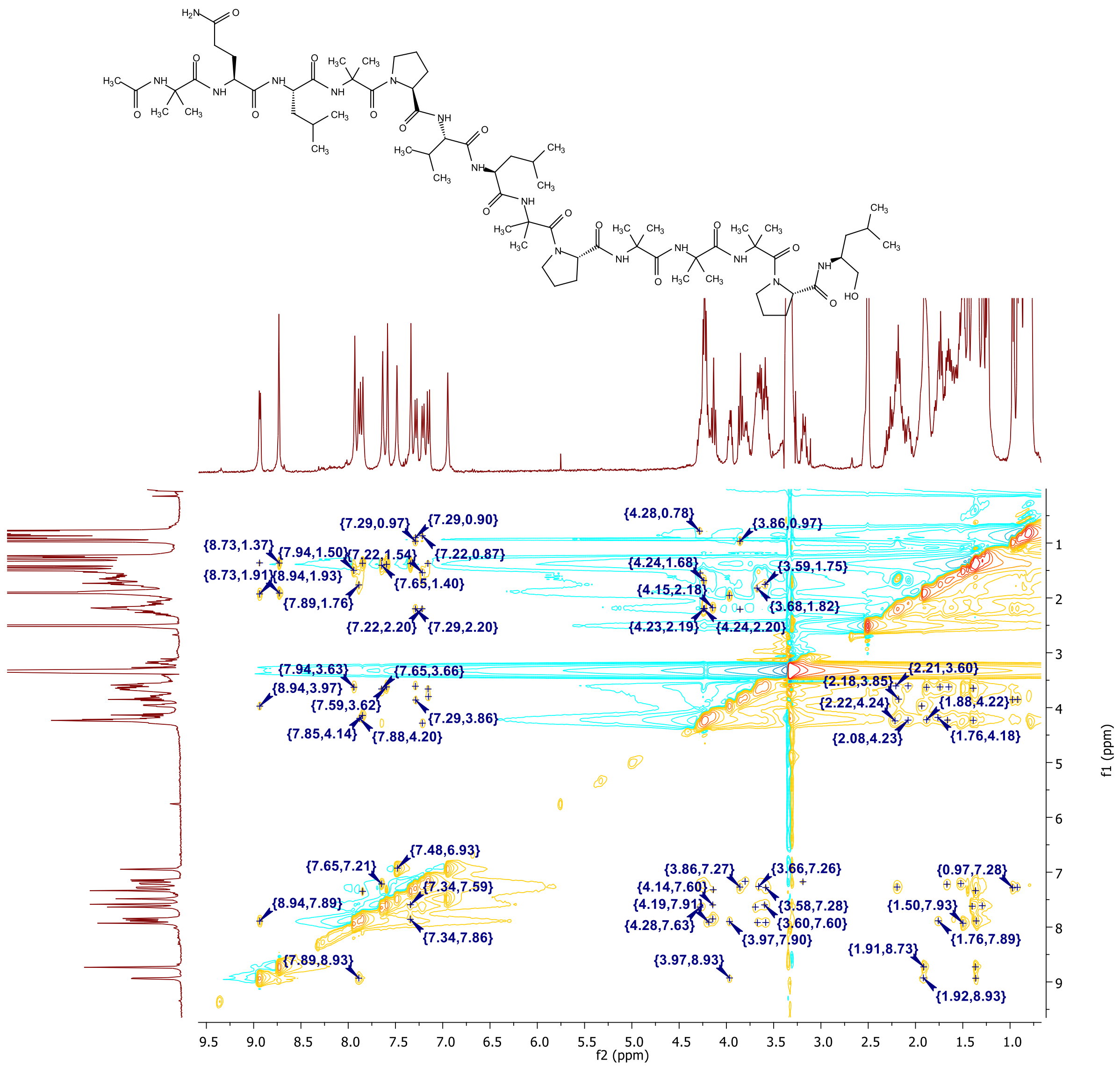

Fig. S11: NOESY spectrum of compound **1** in DMSO-d_6 at 400 MHz for ^1H NMR

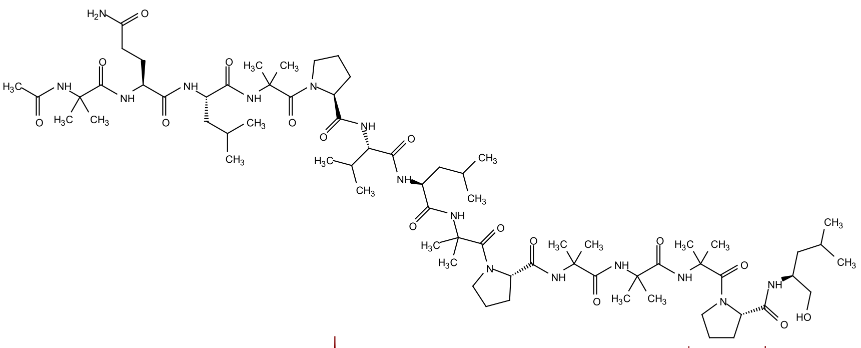

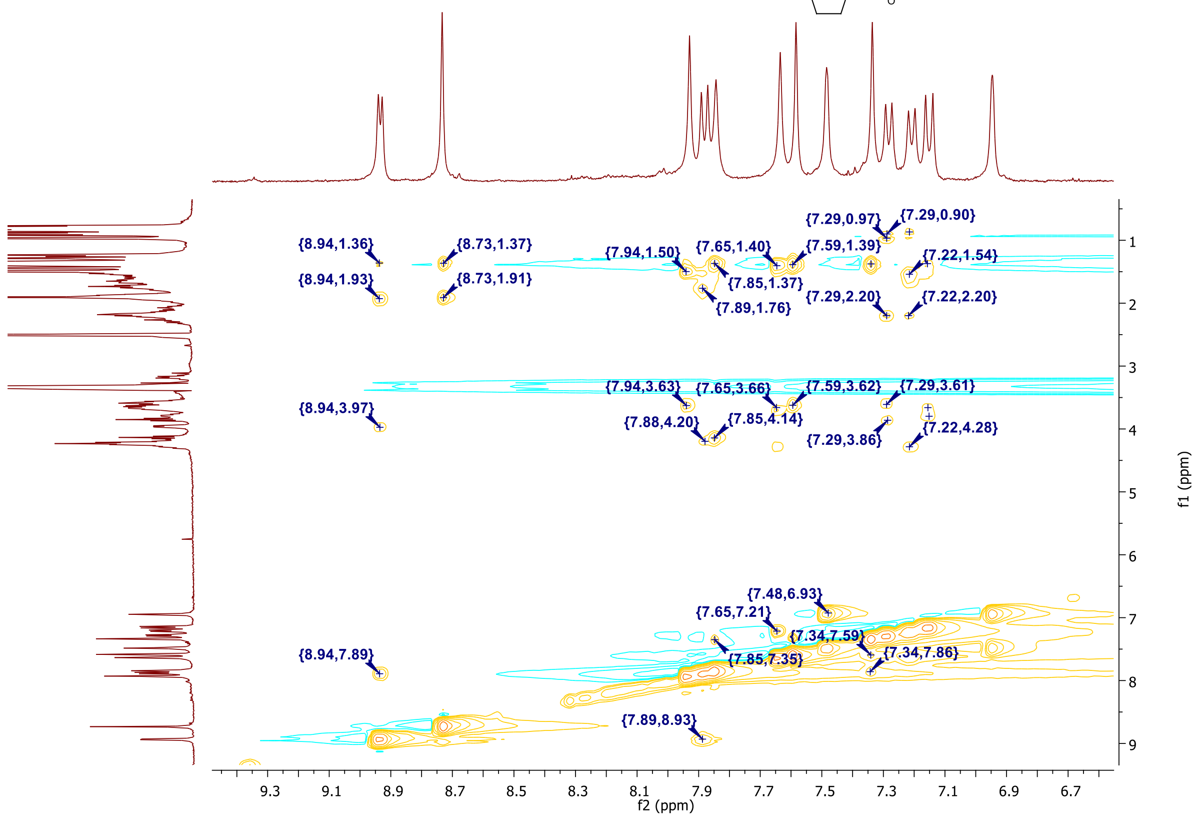

Fig. S11a: Expanded NOESY spectrum of compound **1** in DMSO-d_6 at 400 MHz for ^{1}H NMR

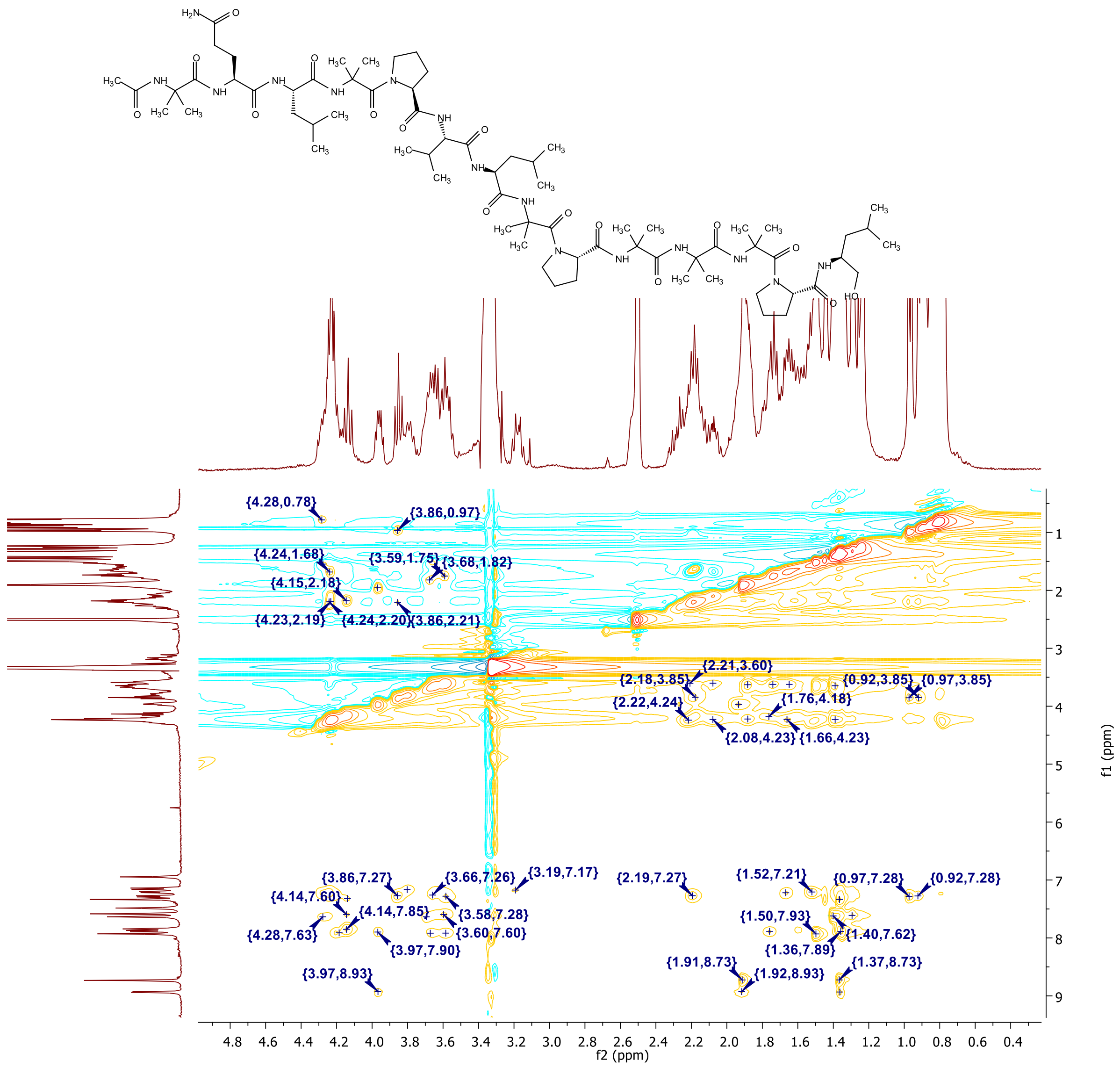

Fig. S11b: Expanded NOESY spectrum of compound **1** in DMSO-d_6 at 400 MHz for ^{1}H NMR

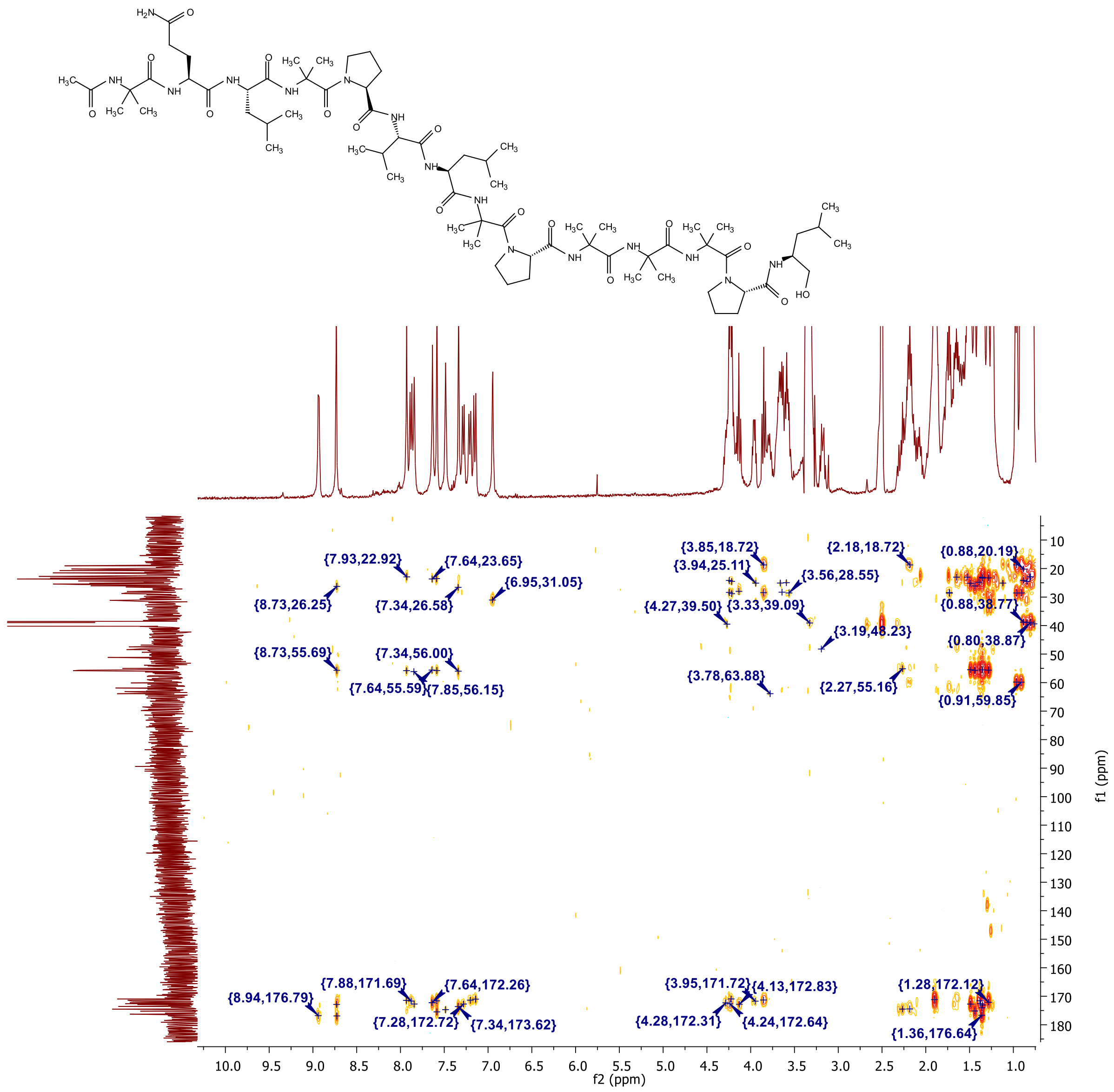

Fig. S12: HMBC spectrum of compound **1** in DMSO-d_6 at 400 MHz for ^{1}H NMR

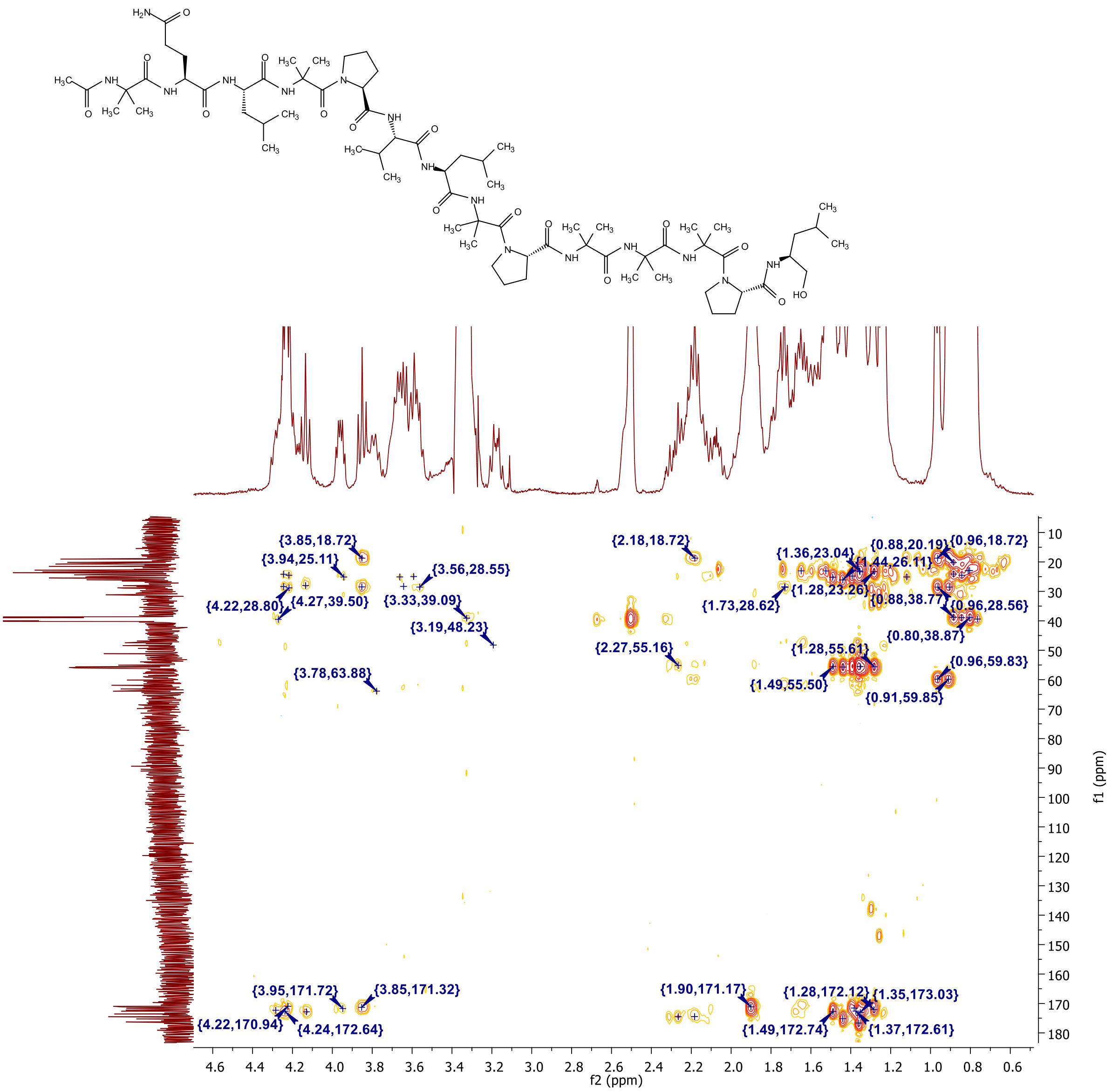

Fig. S12a: Expanded HMBC spectrum of compound **1** in DMSO-d_6 at 400 MHz for ^{1}H NMR

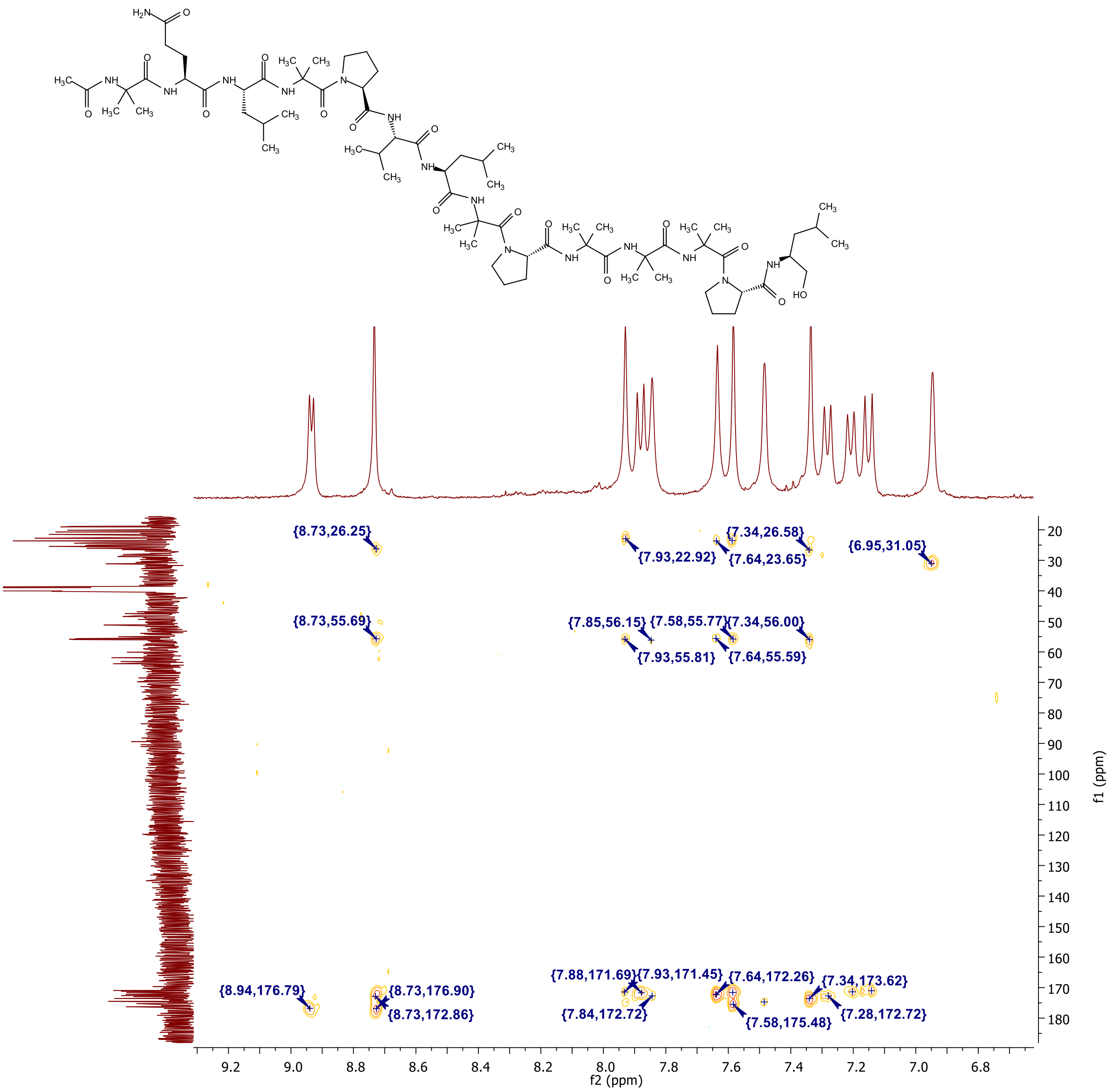

Fig. S12b: Expanded HMBC spectrum of compound **1** in DMSO-d_6 at 400 MHz for ^{1}H NMR

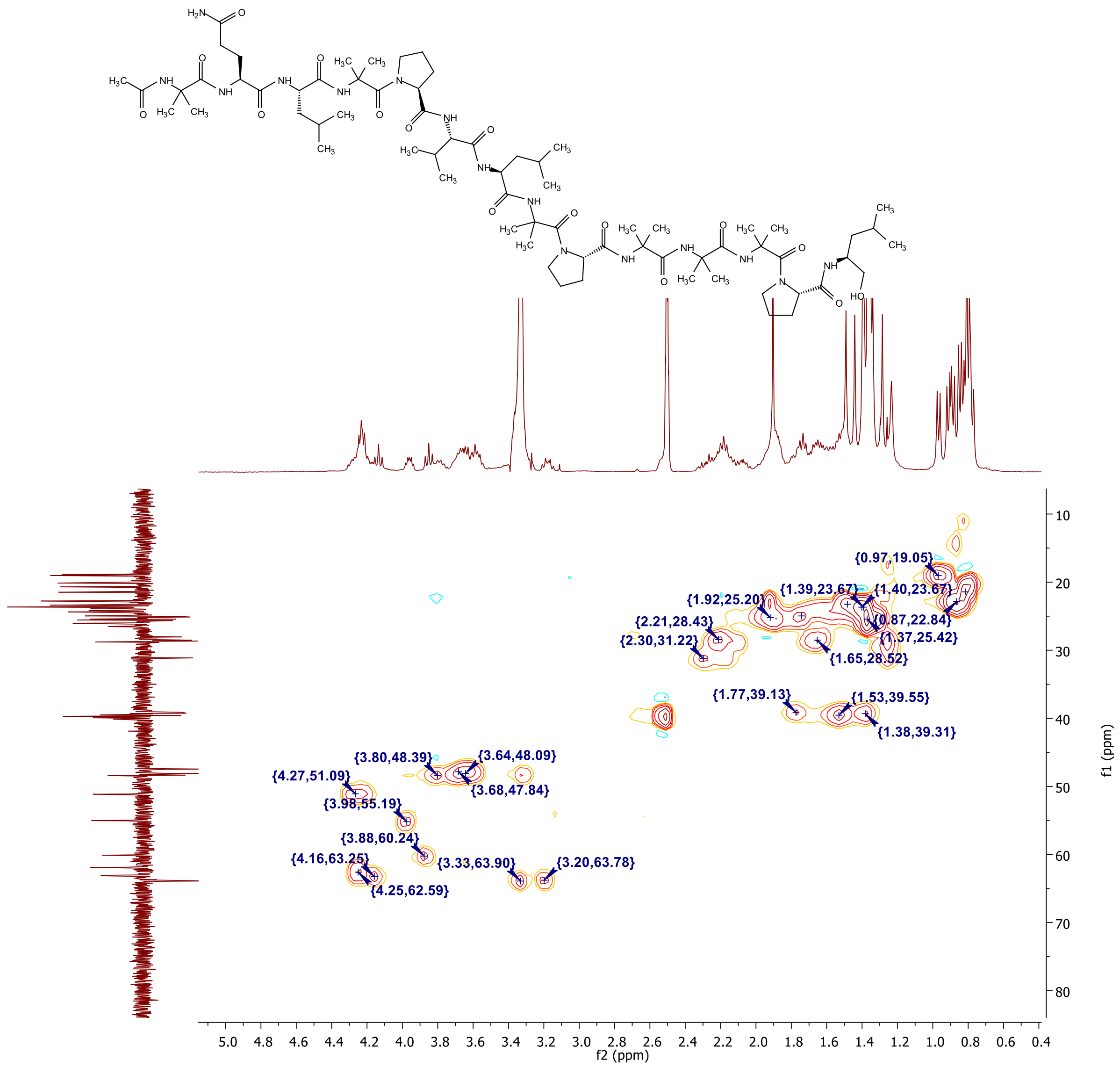

Fig. S13: HSQC spectrum of compound **1** in DMSO-d_6 at 400 MHz for ^{1}H NMR and using DEPT 135 for f1.

Table S1: 2D-COSY, TOCSY and NOESY correlations of **1** at 400MHz for ^{1}H NMR.

Residue		Type	^{1}H (δ), (Mult. *J* in Hz)	COSY Correlations	TOCSY Correlations	NOESY Correlations
Ac	1	C=O	---	---	---	---
	2	CH_3	1.90, (s)	---	---	NH/Aib-1, NH/Gln-2-weak
Aib-1	1	C=O	---	---	---	---
	2	C	---	---	---	---
	3	CH_3	1.34 (s)	---	---	
	4	CH_3	1.36 (s)*	---	---	NH/Aib-1, NH/Gln-2
		NH	8.73 (s)	---	---	4-CH_3/Aib-1, CH_3/Ac
Gln-2	1	C=O		---	---	---
	2	CH	3.96 (m)	NH/Gln-2	3-CH_2, 4-CH_2, NH/ Gln-2	3-CH_2, NH/Gln-2, NH/Leu-3
	3	CH_2	1.96 (m)	2-CH/Gln-2	2-CH/Gln-2	2-CH/Gln-2
	4	CH_2	2.29 (m), 2.18 (m)	3-CH_2/Gln-2	2-CH/Gln-2	---
	5	C=O		---	---	---
		NH_2	7.48(brs), 6.95 (brs)	Self-correlating	Self-correlating	Self-correlating
		NH	8.93 (d, 5.2)	2-CH/Gln-2	2-CH, 3-CH_2/Gln-2	2-CH, 3-CH_2/Gln-2,4-CH_3/Aib-1, NH/Leu-3
Leu-3	1	C=O	---	---	---	---
	2	CH	4.18 (m)	3-CH_2/Leu-3	5-CH_3, NH/ Leu-3	NH/Aib-4
	3	CH_2	1.78 (m), 1.47 (m)			2-CH, NH/Leu-3
	4	CH	1.57 (m)	5-CH_3/Leu-3	6-CH_3/ Leu-3	
	5	CH_3	0.89 (d, 6.8)	4-CH/Leu-3	2-CH, 4-CH/Leu-3	
	6	CH_3	0.79 (m)*	---		
		NH	7.88 (d, 8.4)	2-CH/Leu-3	2-CH, 3-CH_2/Leu-3	2-CH, 3-CH_2/Leu-3, NH/Gln-2
Aib-4	1	C=O	---	---	---	---
	2	C	---	---	---	---
	3	CH_3	1.49 (s)	---	---	NH/Aib-4
	4	CH_3	1.36 (s)*	---	---	
		NH	7.93 (s)	---	---	3-CH_3/Aib-4, 5-CH_2/Pro-5
Pro-5	1	C=O		---	---	---
	2	CH	4.23 (m)	3-CH_2/Pro-5	3-CH_2,4-CH_2, 5-CH_2/Pro-5	3-CH_2/Pro-5
	3	CH_2	2.18 (m), 2.08 (m)		2-CH/Pro-5	2-CH, 5-CH_2/Pro-5
	4	CH_2	1.86 (m), 1.74 (m)		2-CH, 5-CH_2/Pro-5	2-CH, 5-CH_2/Pro-5
	5	CH_2	3.68 (m), 3.58 (m)	4-CH_2/Pro-5	3-CH_2, 4-CH_2/Pro-5	4-CH_2/Pro-5, NH/Aib-4, NH/Val-6
Val-6	1	C=O	---	---	---	---
	2	CH	3.85 (t, 8.0)	NH, 2-CH/Val-6	3-CH, 5-CH_3, NH /Val-6	4-CH_3, NH/Val-6
	3	CH	2.18 (m)	3-CH,4-CH_3/Val-6	5-CH_3/Val-6	2-CH, NH/Val-6
	4	CH_3	0.97 (d, 6.4)	3-CH/Val-6	2-CH, 3-CH, NH/Val-6	2-CH, NH/Val-6
	5	CH_3	0.91 (d, 6.8)	3-CH/Val-6	2-CH, 3-CH, NH/Val-6	2-CH, NH/Val-6
		NH	7.28 (d, 8.4)	2-CH/Val-6	2-CH, 3-CH , 4-CH_3 /Val-6	2-CH, 3-CH,4-CH_3,5-CH_3/Val-6, 5-CH_2/Pro-5
Leu-7	1	C=O	---	---	---	---
	2	CH	4.29 (m)	NH/Leu-7	3-CH_2, 5-CH_3, NH/Leu-7	3-CH_2, 5-CH_3 /Leu-7, NH/Aib-8
	3	CH_2	1.54 (m)		2-CH/Leu-7	NH/leu-7
	4	CH	1.67 (m)	5-CH_3/Leu-7		NH/leu-7
	5	CH_3	0. 79 (m)*	4-CH/Leu-7	2-CH, 3-CH_2/Leu-7	
	6	CH_3	0.85 (d, 6.8)			
		NH	7.21 (d, 8.4)	2-CH/Leu-7	2-CH, 3-CH_2/Leu-7	2-CH, 3-CH_2, 6-CH_3 /Leu-7, 3-CH /Val-6
Aib-8	1	C=O	---	---	---	---
	2	C	---	---	---	---
	3	CH_3	1.40 (s)	---	---	NH/Aib-8
	4	CH_3	1.29 (s)	---	---	NH/Aib-8
		NH	7.63 (s)	---	---	NH (Leu-7)
Pro-9	1	C=O	---	---	---	---
	2	CH	4.13 (t, 8.4)	3-CH_2/Pro-9	3-CH_2, 5-CH_2 /Pro-9	3-CH_2/Pro-9, NH/Aib-8, NH/Aib-10, NH/Aib-11 weak
	3	CH_2	2.20(m), 1.61 (m)		5-CH_2/Pro-9	5-CH_2/Pro-9
	4	CH_2	1.86 (m), 1.74(m)	5-CH_2/Pro-9	5-CH_2/Pro-9	5-CH_2/Pro-9
	5	CH_2	3.68(m), 3.58 (m)	4-CH_2/Pro-9	3-CH_2, 4-CH_2/Pro-9	NH (Aib-8)
Aib-10	1	C=O	---	---	---	---
	2	C	---	---	---	---
	3	CH_3	1.36 (s)*	---	---	
	4	CH_3	1.36 (s)*	---	---	
		NH	7.84 (s)	---	---	4-CH_3/Aib-10, 2-CH/Pro-9, NH/Aib-11

Aib-11	1	C=O	---	---	---	---
	2	C	---	---	---	---
	3	CH_3	1.36 (s)*	---	---	NH/Aib-11
	4	CH_3	1.44 (s)	---	---	
		NH	7.34 (s)	---	---	3-CH_3/Aib-11, NH/Aib-10, NH/Aib-12
Aib-12	1	C=O	---	---	---	---
	2	C	---	---	---	---
	3	CH_3	1.39 (s)	---	---	2-CH/Pro-13, 5-CH_2/Pro-13
	4	CH_3	1.36 (s)*	---	---	
		NH	7.58 (s)	---	---	5-CH_2/Pro-13
Pro-13	1	C=O	---	---	---	
	2	CH	4.23 (m)	3-CH_2/Pro-13	3-CH_2, 4-CH_2/Pro-13	3-CH_2/Pro-13
	3	CH_2	2.18(m), 2.08 (m)		2-CH, 5-CH_2/Pro-13	5-CH_2/Pro-13
	4	CH_2	1.86(m), 1.74 (m)		2-CH, 5-CH_2/Pro-13	2-CH/Pro-13, 5-CH_2/Pro-13
	5	CH_2	3.68(m), 3.58 (m)	4-CH_2/Pro-13	3-CH_2, 4-CH_2/Pro-13	4-CH_2/Pro-13
Leuol	1	CH	3.77 (m)	2-CH_2,NH/Leuol	2-CH_2/Leuol	NH/Leuol
	2	CH_2	1.37 (m)	1-CH/Leuol	1-CH, 4-CH_3, 6-CH_2, NH /Leuol	
	3	CH	1.67 (m)	5-CH_3 /Leuol	OH	OH
	4	CH_3	0.82 (m)*		2-CH_2/Leuol	
	5	CH_3	0.79 (m)*	3-CH/Leuol		
	6	*CH_2* OH	3.27(m), 3.18 (m)	OH	2-CH_2, NH/Leuol	NH/Leuol
		NH	7.15 (d, 9.6)	1-CH/ Leuol	1-CH,2-CH_2, 6-CH_2 /Leuol	1-CH, 2-CH_2/Leuol, 5-CH_2/Pro-13
		OH	4.23 (m)	6-CH_2/Leuol		

[a]From DEPT-135, *signal overlap

Table S2: 2D-HMBC correlations of **1** at 400 MHz for ^{1}H NMR and 100 MHz for ^{13}C NMR

Residue		Type	^{13}C (*δ*)	^{1}H (*δ*), (Mult. *J* in Hz)	HMBC Correlations
Ac	1	C=O	171.2	---	---
	2	CH_3	22.9	1.90, (s)	CO/Ac
Aib-1	1	C=O	176.3	---	---
	2	C	55.9	---	---
	3	CH_3	23.6*	1.34 (s)	
	4	CH_3	26.5	1.36 (s)*	CO/Aib-1
		NH		8.73 (s)	CO, 2-C, 4-CH_3 /Aib-1, CO/Gln-2
Gln-2	1	C=O	172.6		---
	2	CH	55.0	3.96 (m)	3-CH_2/Gln-2, CO/Leu-3
	3	CH_2	25.0	1.97 (m)	---
	4	CH_2	31.1	2.29 (m), 2.18 (m)	2-CH, 5-CO/Gln-2
	5	C=O	174.5		---
		NH_2		7.48 (br s), 6.95 (br s)	4-CH_2, 5-CO/Gln-2
		NH		8.93 (d, 5.2)	CO/Aib-1
Leu-3	1	C=O	171.7		---
	2	CH	51.2	4.18 (m)	---
	3	CH_2	39.1[a]	1.78 (m), 1.47 (m)	5-CH_3/Leu-3
	4	CH	24.2	1.57 (m)	
	5	CH_3	22.8	0.89 (d, 6.8)	3-CH_2, 4-CH/Leu-3
	6	CH_3	20.7	0.79 (m)*	5-CH_3/Leu-3
		NH		7.88 (d, 8.4)	CO/Leu-3
Aib-4	1	C=O	173.6		---
	2	C	55.8		---
	3	CH_3	23.4	1.49 (s)	2-C, 4-CH_3/Aib-4, CO/Pro-5
	4	CH_3	25.4	1.36 (s)*	2-C, 3-CH_3/Aib-4
		NH		7.93 (s)	2-C, 3-CH_3/Aib-4, CO/Leu-3
Pro-5	1	C=O	172.7		---
	2	CH	63.1	4.23 (m)	CO, 3-CH_2/Pro-5
	3	CH_2	28.7	2.18 (m), 2.08 (m)	
	4	CH_2	25.4[a]	1.86 (m), 1.74 (m)	3-CH_2/Pro-5
	5	CH_2	48.4	3.68 (m), 3.58 (m)	
Val-6	1	C=O	171.4		---
	2	CH	60.1	3.85 (t, 8.0)	CO, 3-CH, 5-CH_3/Val-6
	3	CH	28.8	2.18 (m)	5-CH_3/Val-6
	4	CH_3	19.0	0.97 (d, 6.4)	2-CH, 3-CH, 5-CH_3/Val-6,
	5	CH_3	18.9	0.91 (d, 6.8)	2-CH, 3-CH/Val-6
		NH		7.28 (d, 8.4)	CO/Pro-5
Leu-7	1	C=O	172.2		---
	2	CH	51.2	4.29 (m)	CO, 3-CH_2 /Leu-7
	3	CH_2	39.6[a]	1.54 (m)	6-CH_3/Leu-7
	4	CH	24.4	1.67 (m)	
	5	CH_3	21.5	0. 79 (m)*	3-CH_2, 6-CH_3/Leu-7,
	6	CH_3	22.8	0.85 (d, 6.8)	3-CH_2, 4-CH/Leu-7
		NH		7.21 (d, 8.4)	CO/Val-6
Aib-8	1	C=O	172.1		---
	2	C	55.6		---
	3	CH_3	23.7*	1.40 (s)	
	4	CH_3	25.6	1.29 (s)	CO, 2-C/Aib-8
		NH		7.63 (s)	CO/Leu-7, 2-C, 3-CH_3/Aib-8
Pro-9	1	C=O	172.9		---
	2	CH	63.2	4.13 (t, 8.4)	CO, 3-CH_2/Pro-9
	3	CH_2	28.5	2.20(m), 1.61 (m)	
	4	CH_2	25.1	1.86 (m), 1.74(m)	3-CH_2/Pro-9
	5	CH_2	48.1	3.68(m), 3.58 (m)	3-CH_2, 4-CH_2/Pro-9
Aib-10	1	C=O	173.6		---
	2	C	56.1		---
	3	CH_3	25.6[a]	1.36 (s)*	
	4	CH_3	23.6*	1.36 (s)*	CO/Aib-10
		NH		7.84 (s)	CO/Pro-9, 2-C/Aib-10

Aib-11	1	C=O	175.5		---
	2	C	56.0		---
	3	CH_3	26.5	1.36 (s)*	
	4	CH_3	23.6	1.44 (s)	CO/Aib-11
		NH		7.34 (s)	CO/Aib-10, 3-CH_3/Aib-11
Aib-12	1	C=O	171.6		---
	2	C	55.7		---
	3	CH_3	23.7*	1.39 (s)	CO, 4-CH_3/Aib-12
	4	CH_3	25.0	1.36 (s)*	
		NH		7.58 (s)	CO, 2-C, 3-CH_3/Aib-12, CO/Aib-11
Pro-13	1	C=O	170.9		---
	2	CH	61.9	4.23 (m)	CO/Pro-13
	3	CH_2	28.1	2.18(m), 2.08 (m)	
	4	CH_2	25.6[a]	1.86(m), 1.74 (m)	
	5	CH_2	48.4	3.68(m), 3.58 (m)	3-CH_2/Pro-13
Leuol	1	CH	48.4	3.77 (m)	6-CH_2OH/Leuol weak
	2	CH_2	39.3[a]	1.37 (m)	
	3	CH	23.8	1.67 (m)	4-CH_3/Leuol
	4	CH_3	22.8	0.82 (m)*	
	5	CH_3	20.2	0.79 (m)*	2-CH_2/Leuol
	6	*CH_2*OH	63.9	3.27(m), 3.18 (m)	1-CH, 2-CH_2/Leuol
		NH		7.15 (d, 9.6)	CO/Pro-13
		OH		4.23 (m)	

[a]From DEPT-135, *signal overlap

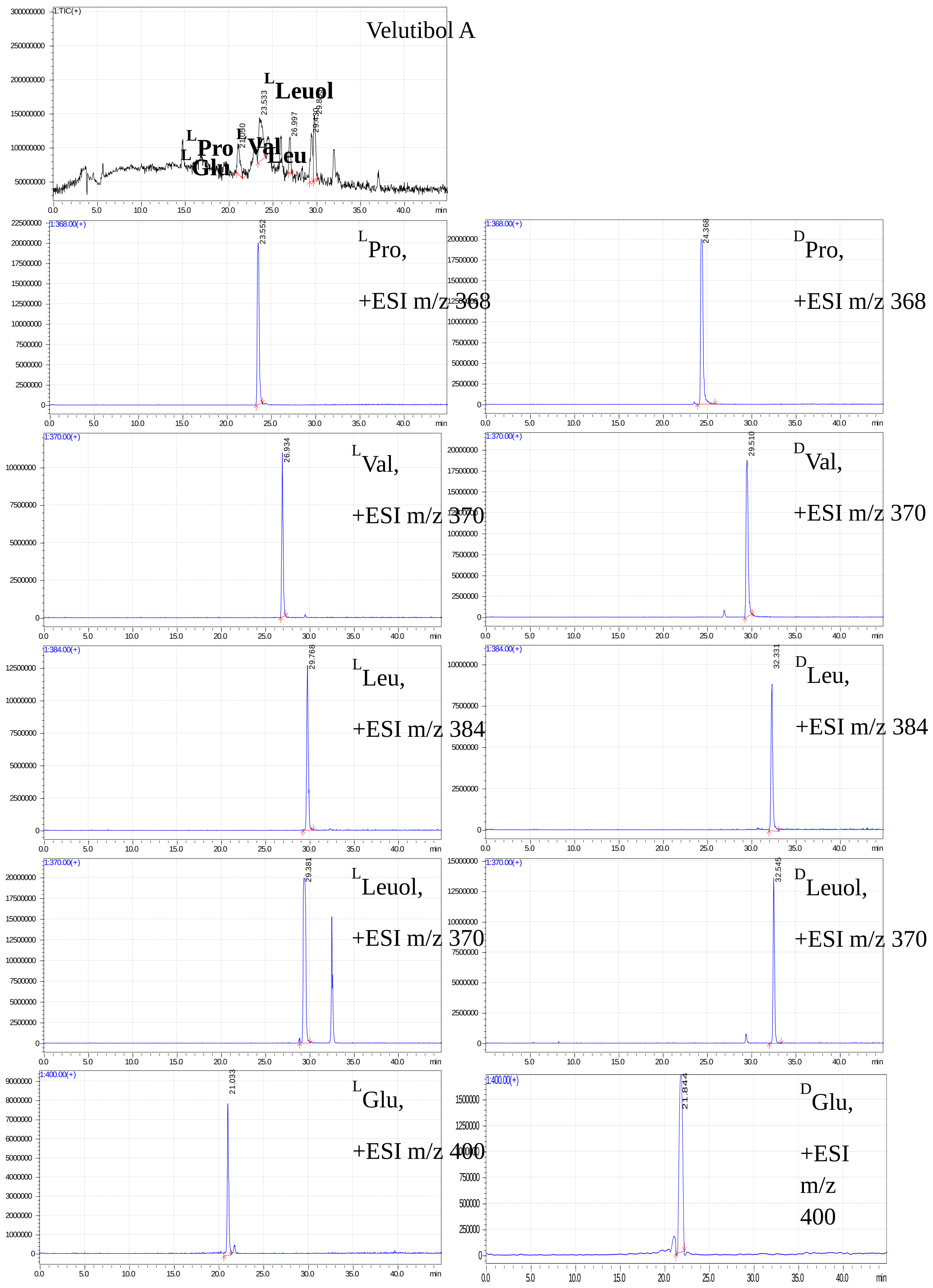

Fig. S14: Marfey's analysis of **1** using LCMS.

Analysis Report

<Sample Information>

Sample Name	: B **VELUTIBOL A (1)**		
Sample ID	: B		
Data Filename	: B.lcd		
Method Filename	: 10-60 ACN63.lcm		
Batch Filename	: BATCH SAMPLE, 07-01-2019.lcb		
Vial #	: 1-47	Sample Type	: Unknown
Injection Volume	: 5 uL		
Date Acquired	: 07-01-2019 22:33:26	Acquired by	: System Administrator
Date Processed	: 07-01-2019 23:36:29	Processed by	: System Administrator

<Chromatogram>

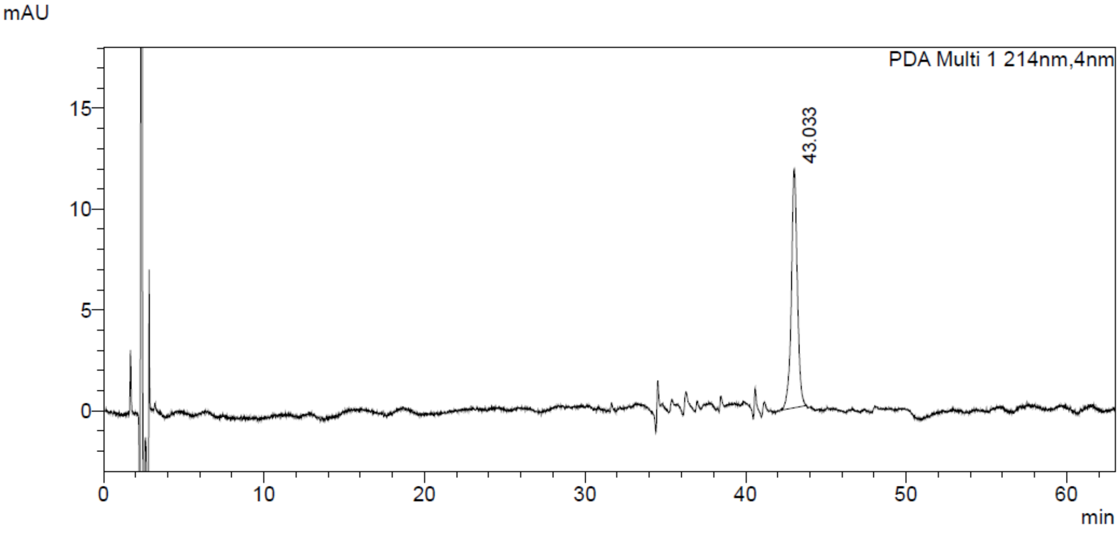

<Peak Table>

PDA Ch1 214nm

Peak#	Ret. Time	Area	Height	Area%
1	43.033	307734	11855	100.000
Total		307734	11855	100.000

Fig. S15: HPLC purity of compound **1**.

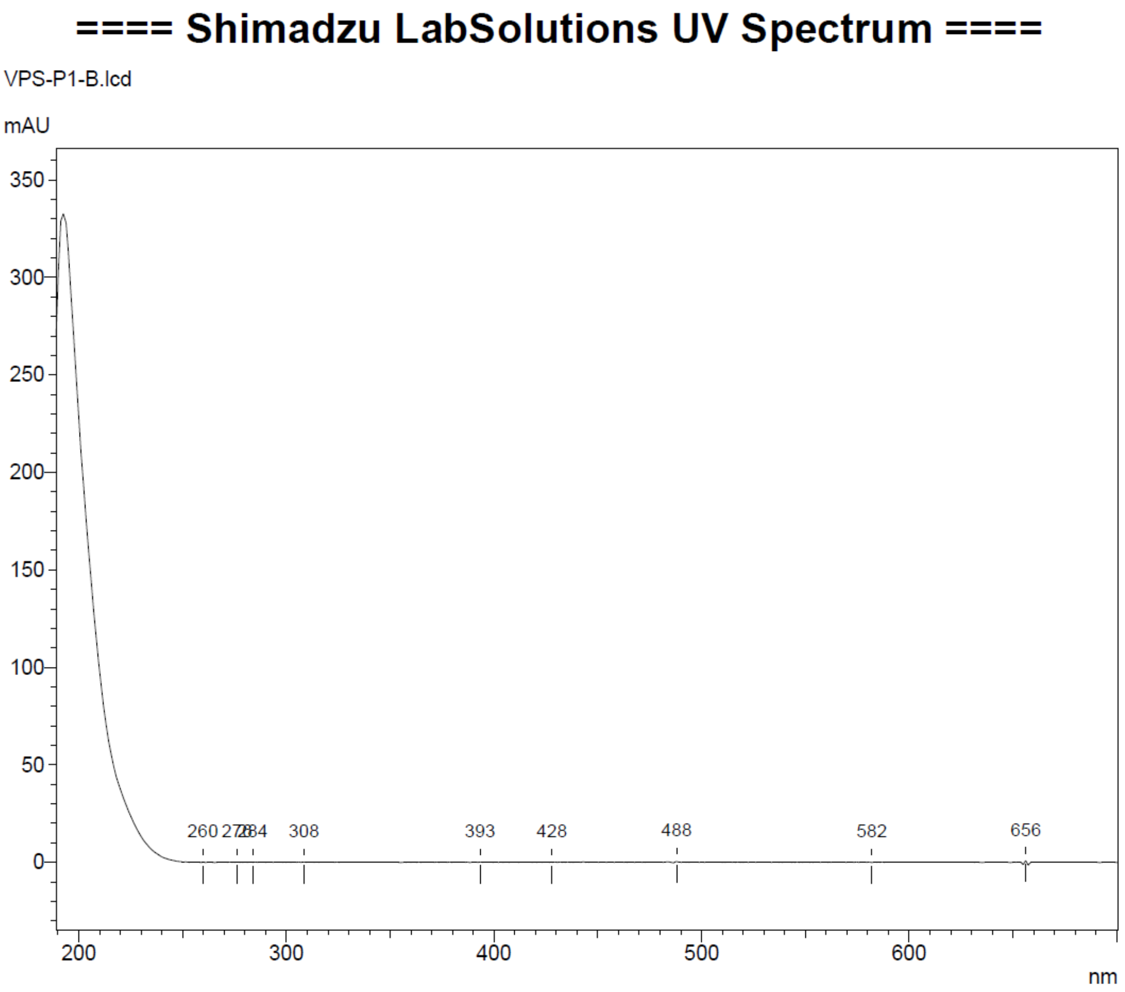

Fig. S16: UV-spectrum of compound **1**.

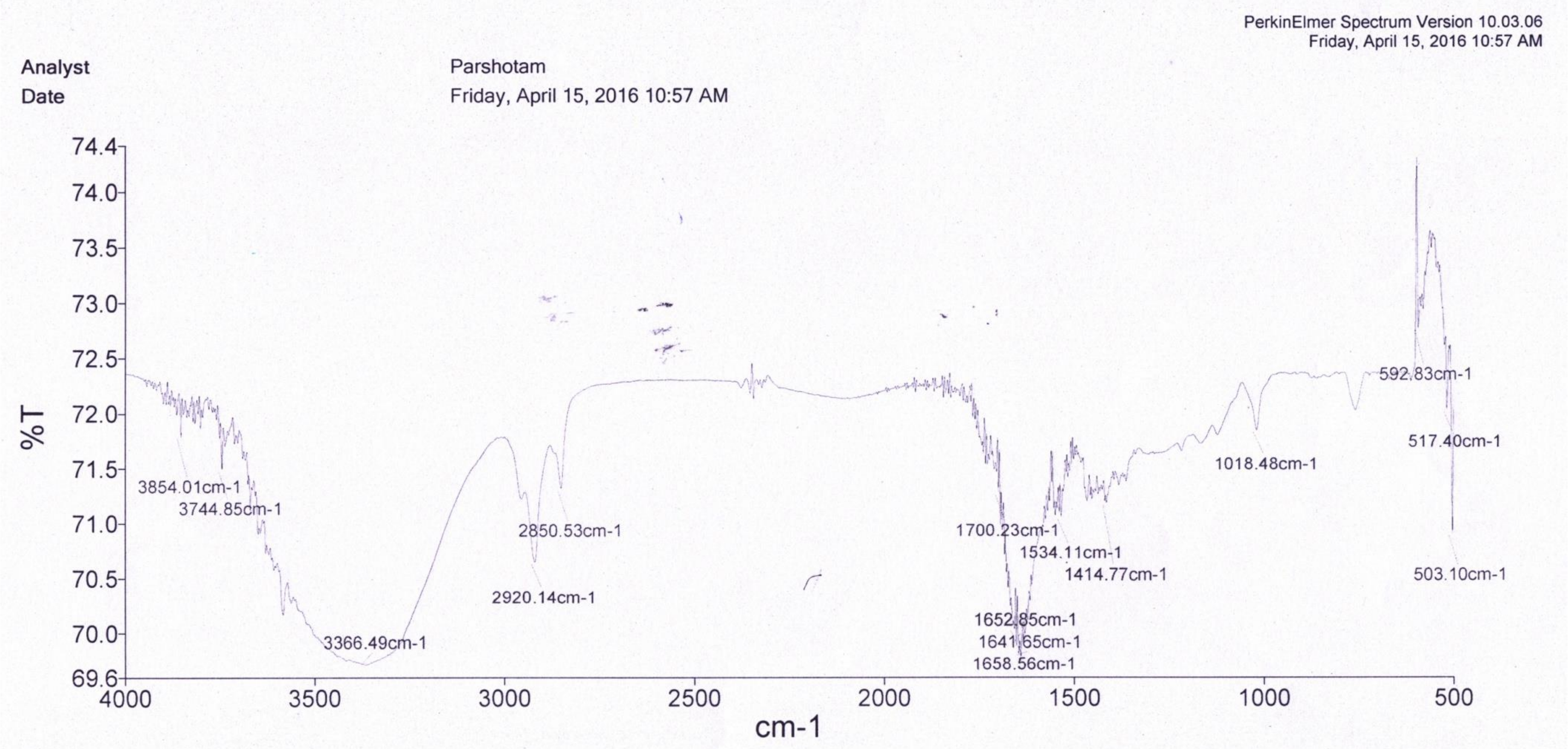

Sample Name	Description	Quality Checks
VPS-P1-B	CHCL3	The Quality Checks give rise to multiple warnings for the sample.

Fig. S17: IR spectrum of compound **1** in $CHCl_3$

SHIMADZU LabSolutions

Analysis Report

<Sample Information>

Sample Name	: B1 **VELUTIBOL B (2)**		
Sample ID	: B1		
Data Filename	: B1.lcd		
Method Filename	: 10-60 ACN63.lcm		
Batch Filename	: BATCH SAMPLE, 07-01-2019.lcb		
Vial #	: 1-56	Sample Type	: Unknown
Injection Volume	: 5 uL		
Date Acquired	: 08-01-2019 09:08:28	Acquired by	: System Administrator
Date Processed	: 08-01-2019 10:11:32	Processed by	: System Administrator

<Chromatogram>

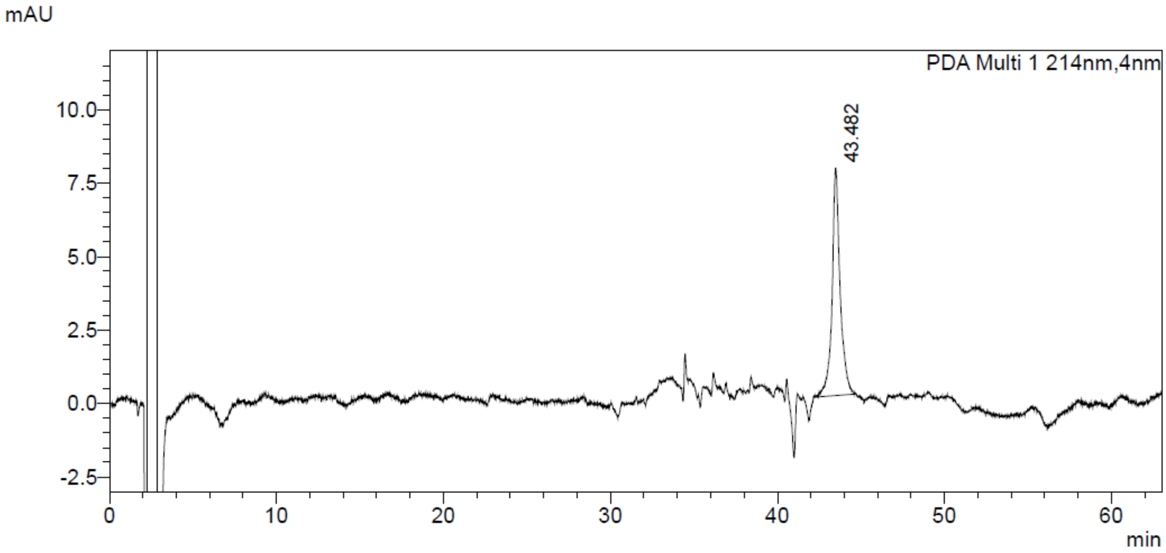

<Peak Table>

PDA Ch1 214nm

Peak#	Ret. Time	Area	Height	Area%
1	43.482	260648	7738	100.000
Total		260648	7738	100.000

Fig. S18: HPLC chromatogram of **2**

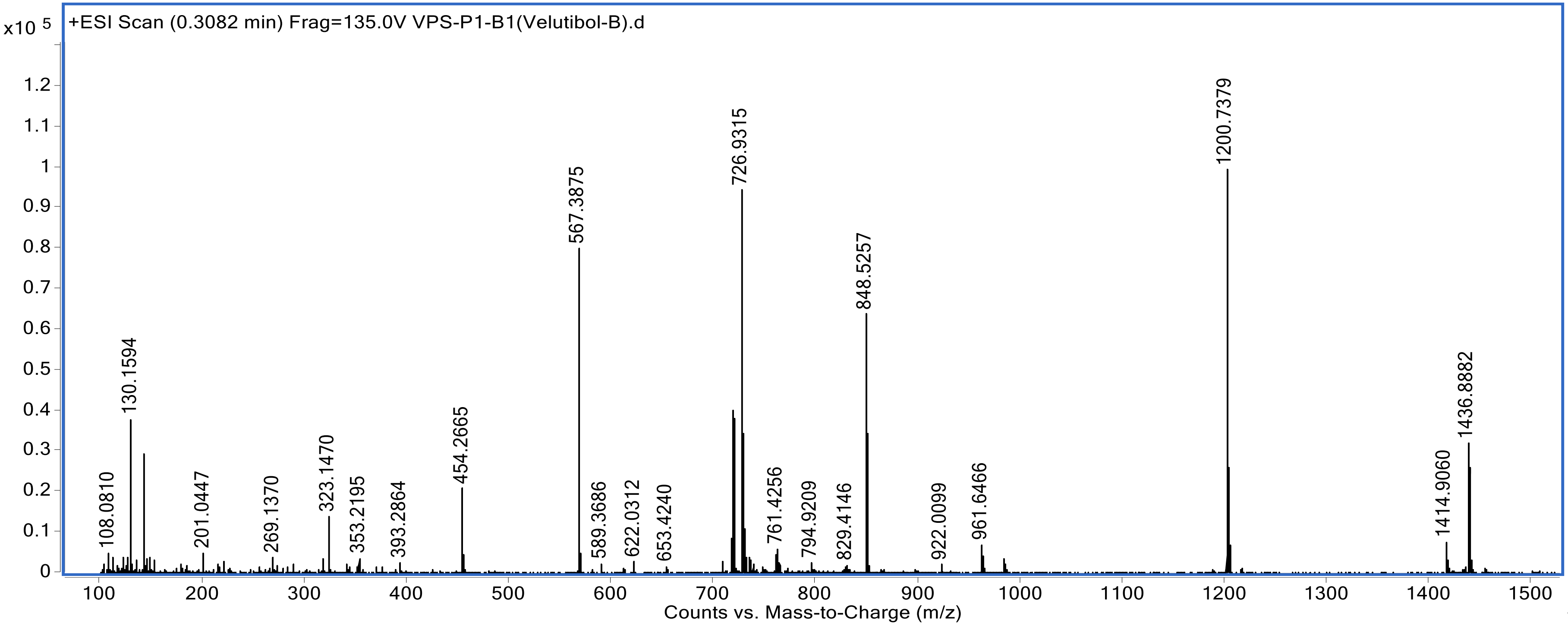

F

ig. S19: HRMS of 2

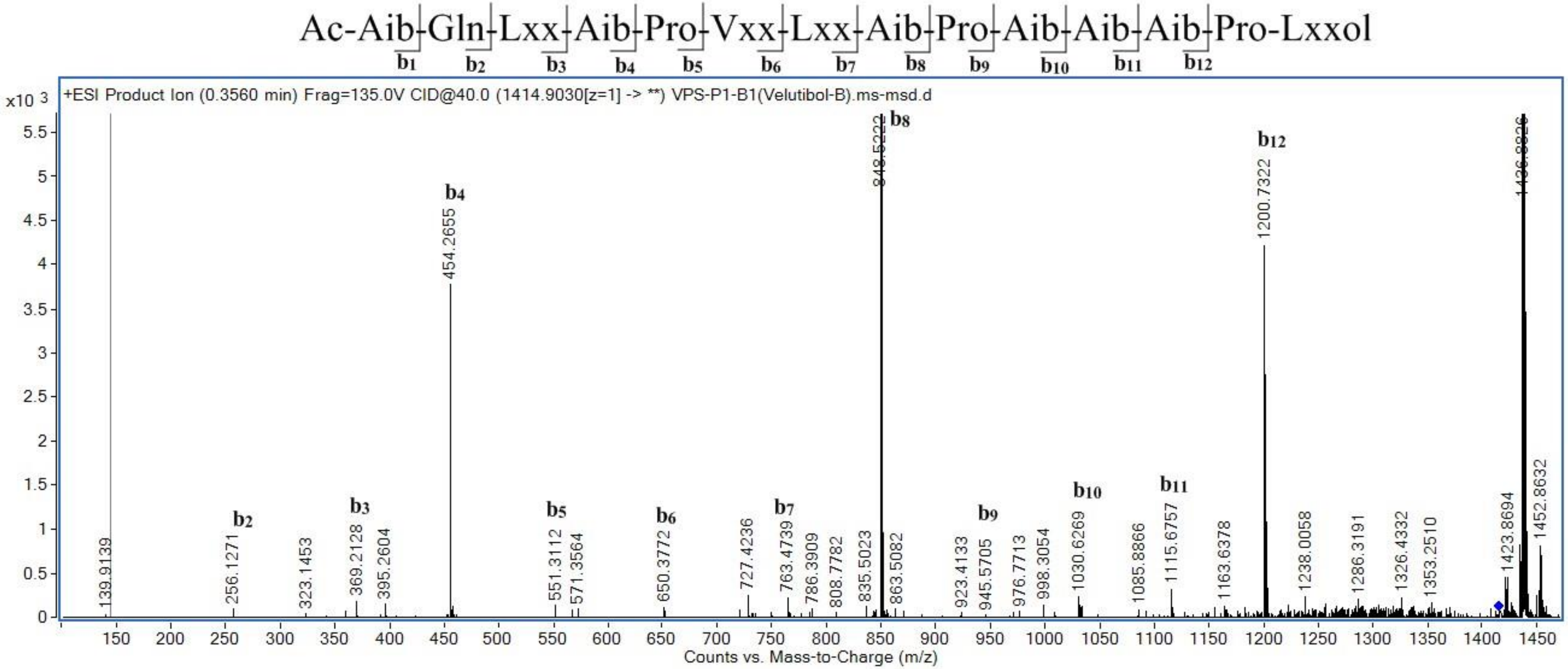

Fig. S20a: MS/MS of compound **2** for *m/z* 1414.9030 $[M+H]^+$.

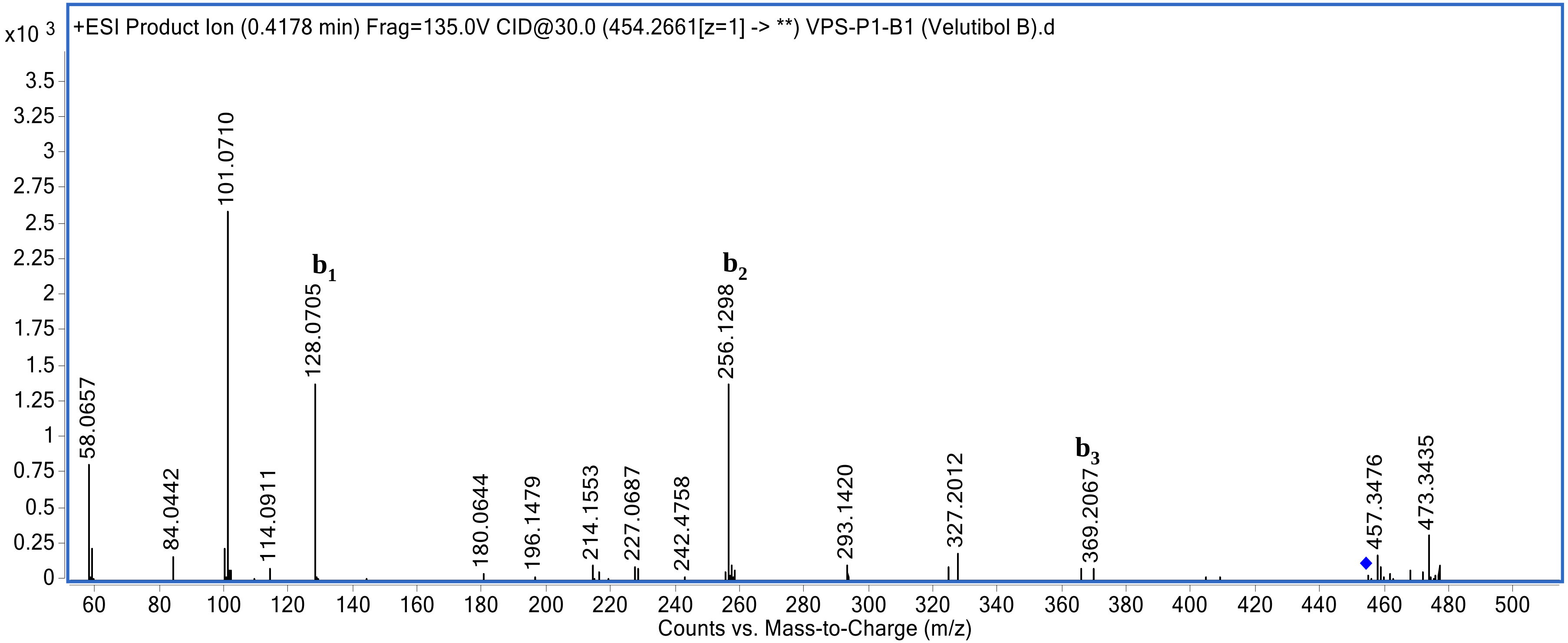

Fig. S20b: MS/MS of *m/z* 454.2661 daughter ion b_4 for compound **2.**

SHIMADZU LabSolutions **Analysis Report**

<Sample Information>

Sample Name : D1 **VELUTIBOL C (3)**
Sample ID : D1
Data Filename : D1.lcd
Method Filename : 10-60 ACN63.lcm
Batch Filename : BATCH SAMPLE, 07-01-2019.lcb
Vial # : 1-50
Sample Type : Unknown
Injection Volume : 50 uL
Date Acquired : 08-01-2019 02:47:26
Acquired by : System Administrator
Date Processed : 08-01-2019 03:50:29
Processed by : System Administrator

<Chromatogram>

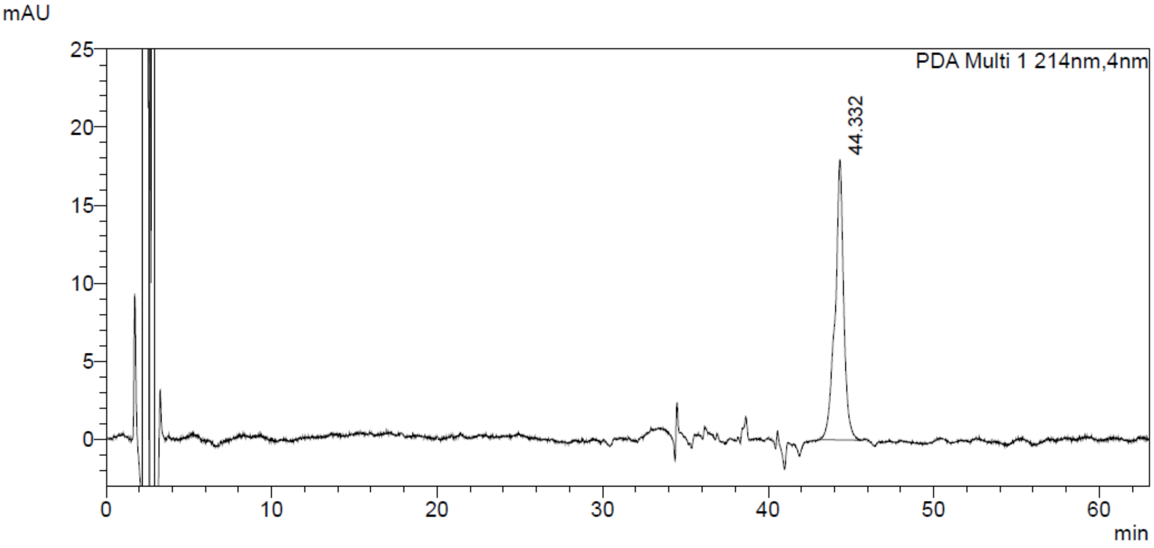

<Peak Table>

PDA Ch1 214nm

Peak#	Ret. Time	Area	Height	Area%
1	44.332	652508	17962	100.000
Total		652508	17962	100.000

Fig. S21: HPLC chromatogram of **3**

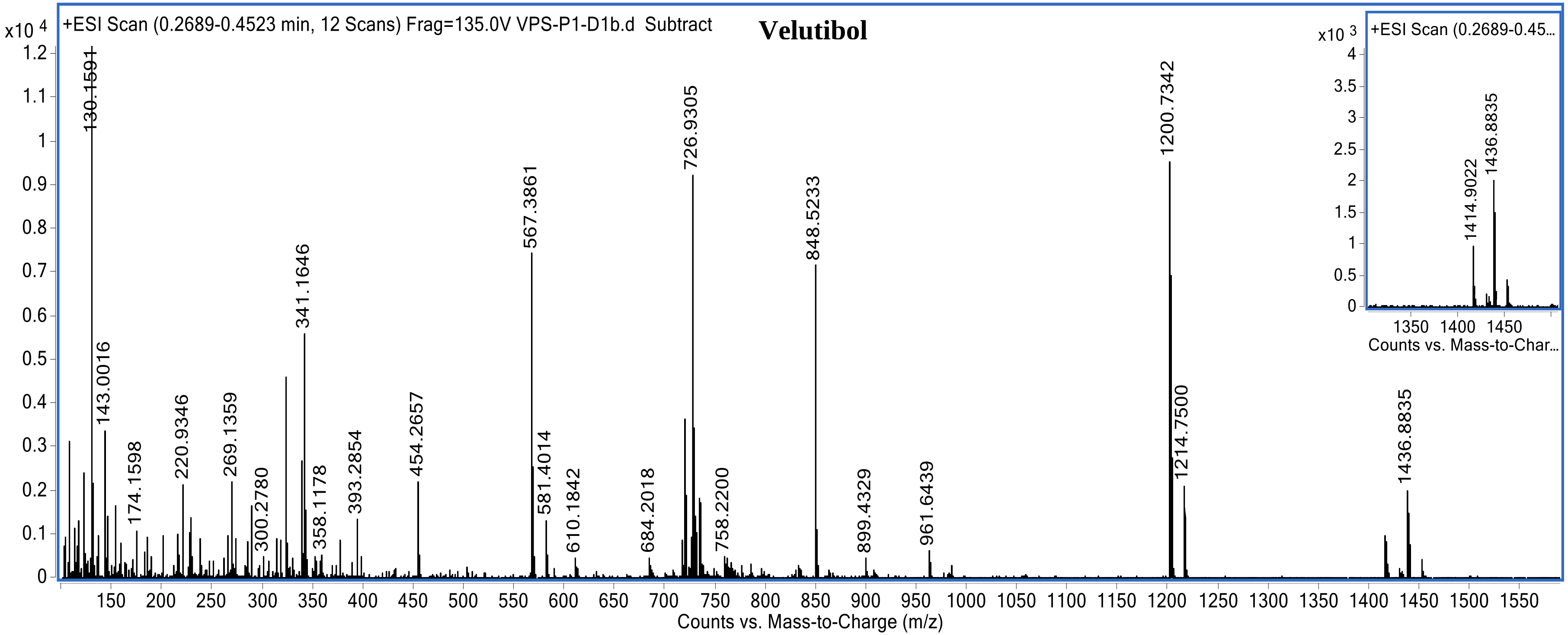

Fig. S22: HRMS of **3.**

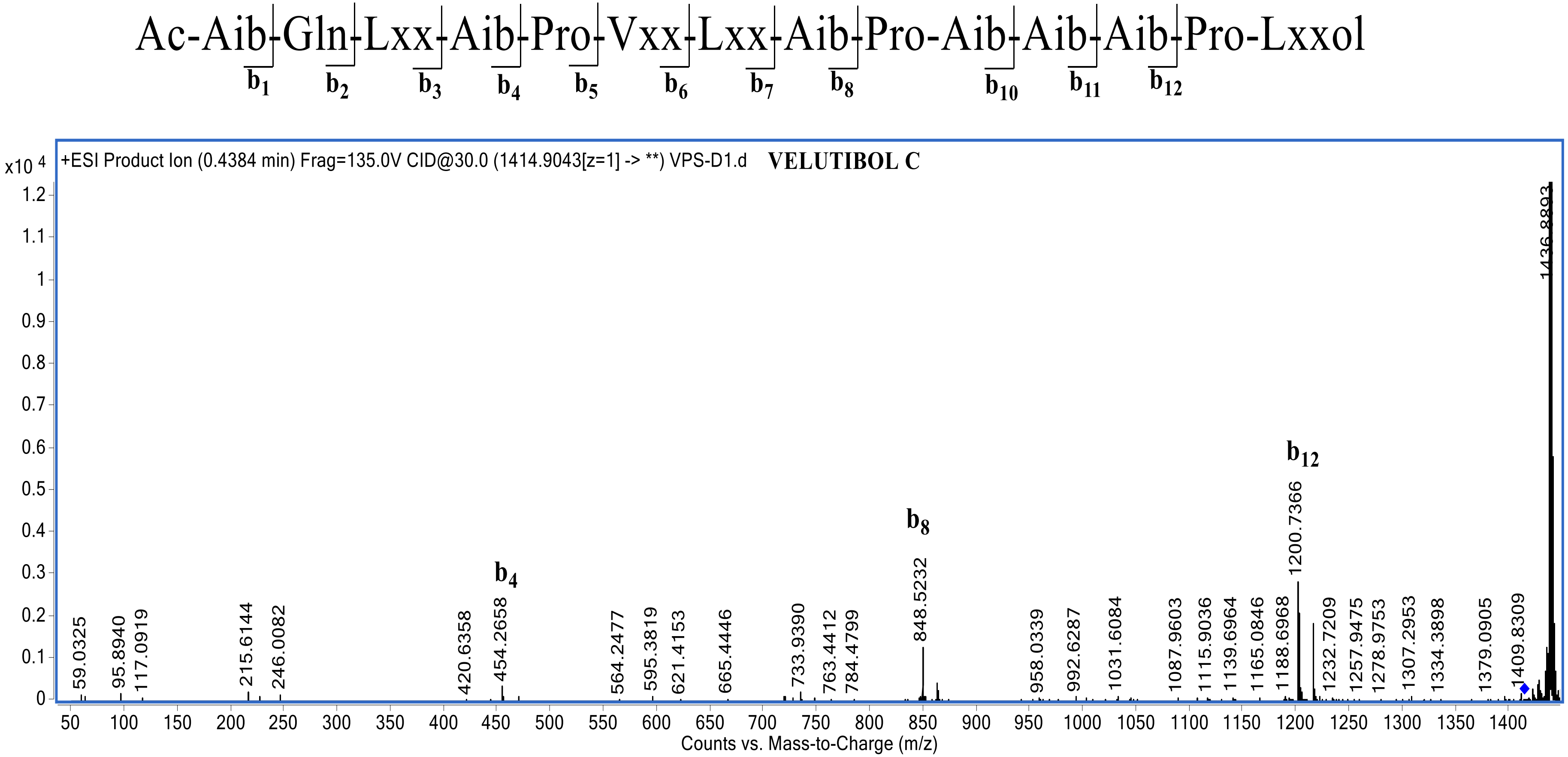

Fig. S23a: MS/MS of compound **3** for *m/z* 1414.9043 $[M + H]^+$.

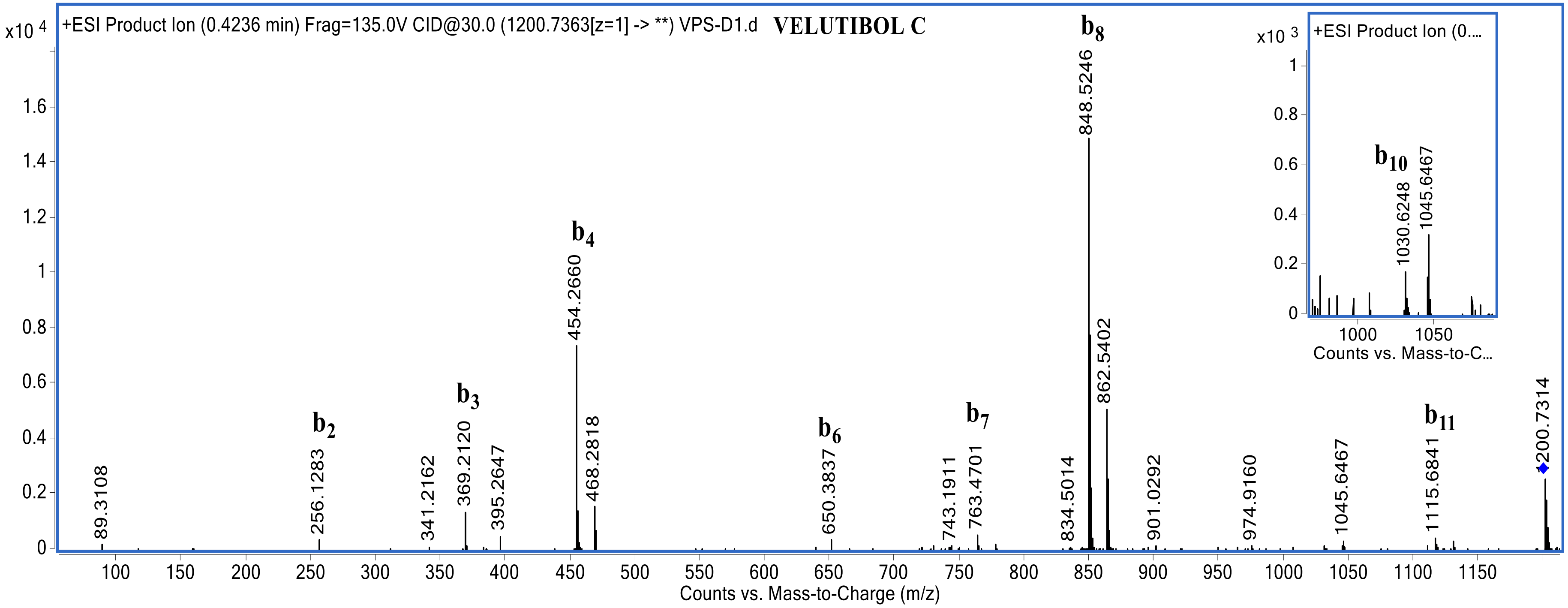

Fig. S23b: MS/MS of *m/z* 1200.7363 daughter ion b_{12} for compound **3.**

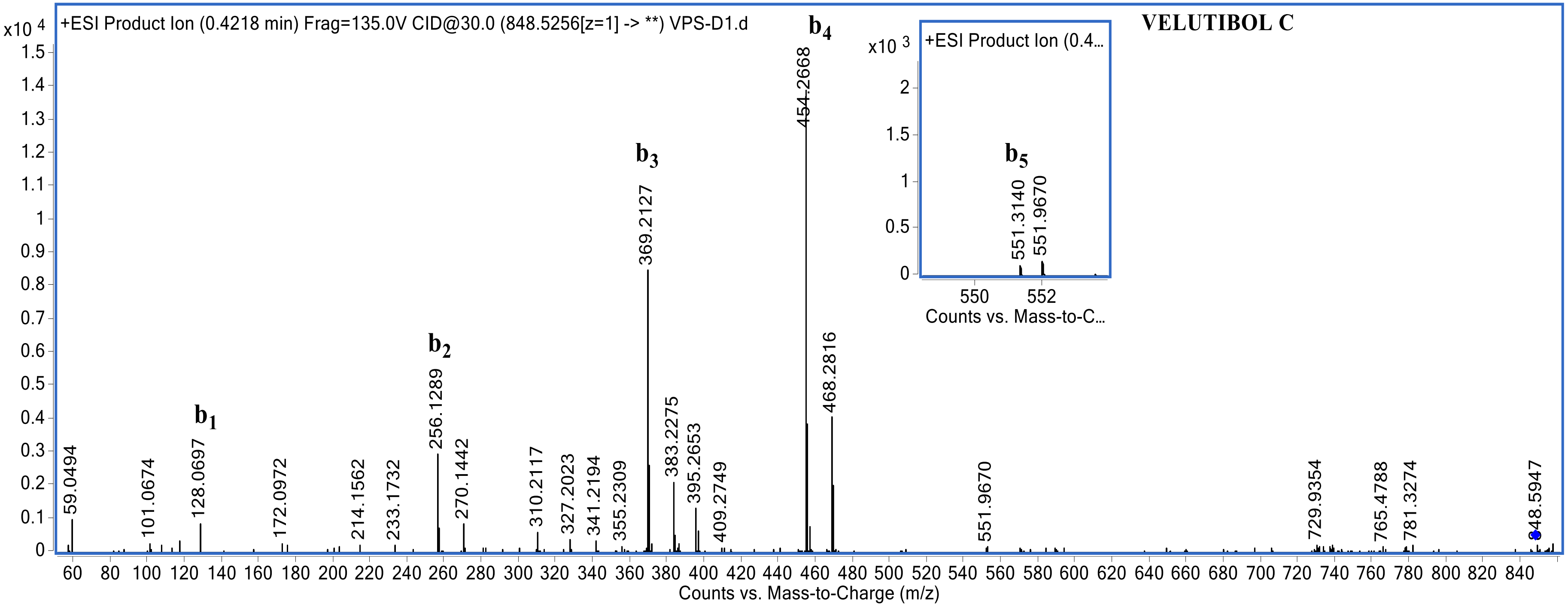

Fig. S23c: MS/MS of *m/z* 848.5256 daughter ion b_8 for compound **3.**

SHIMADZU LabSolutions **Analysis Report**

<Sample Information>

Sample Name	: D2 **VELUTIBOL D**		
Sample ID	: D2		
Data Filename	: D2.lcd		
Method Filename	: 10-60 ACN63.lcm		
Batch Filename	: BATCH SAMPLE, 07-01-2019.lcb		
Vial #	: 1-51	Sample Type	: Unknown
Injection Volume	: 50 uL		
Date Acquired	: 08-01-2019 03:50:55	Acquired by	: System Administrator
Date Processed	: 08-01-2019 04:54:00	Processed by	: System Administrator

<Chromatogram>

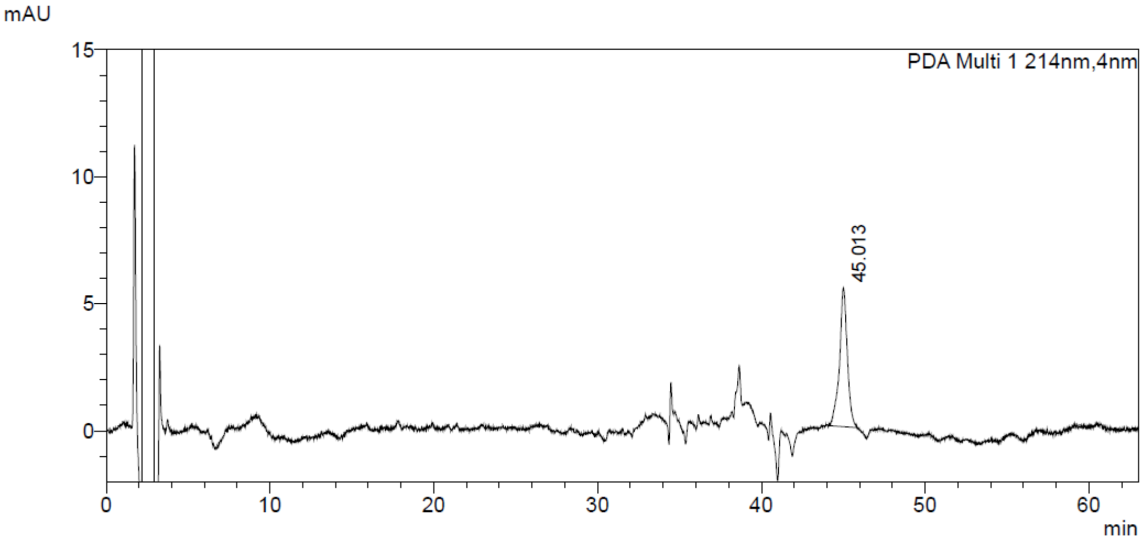

<Peak Table>

PDA Ch1 214nm

Peak#	Ret. Time	Area	Height	Area%
1	45.013	183701	5470	100.000
Total		183701	5470	100.000

Fig. S24: HPLC chromatogram of compound **4.**

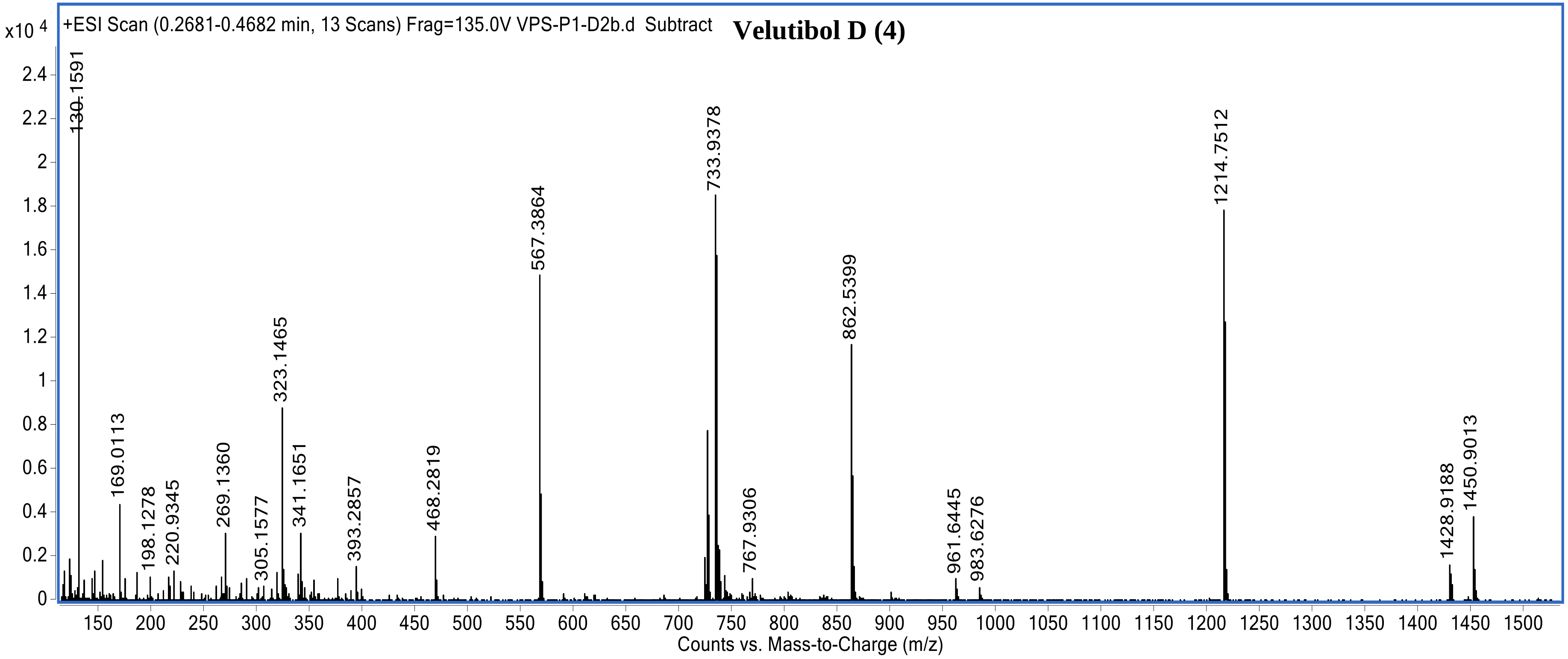

Fig. S25: HRMS of **4.**

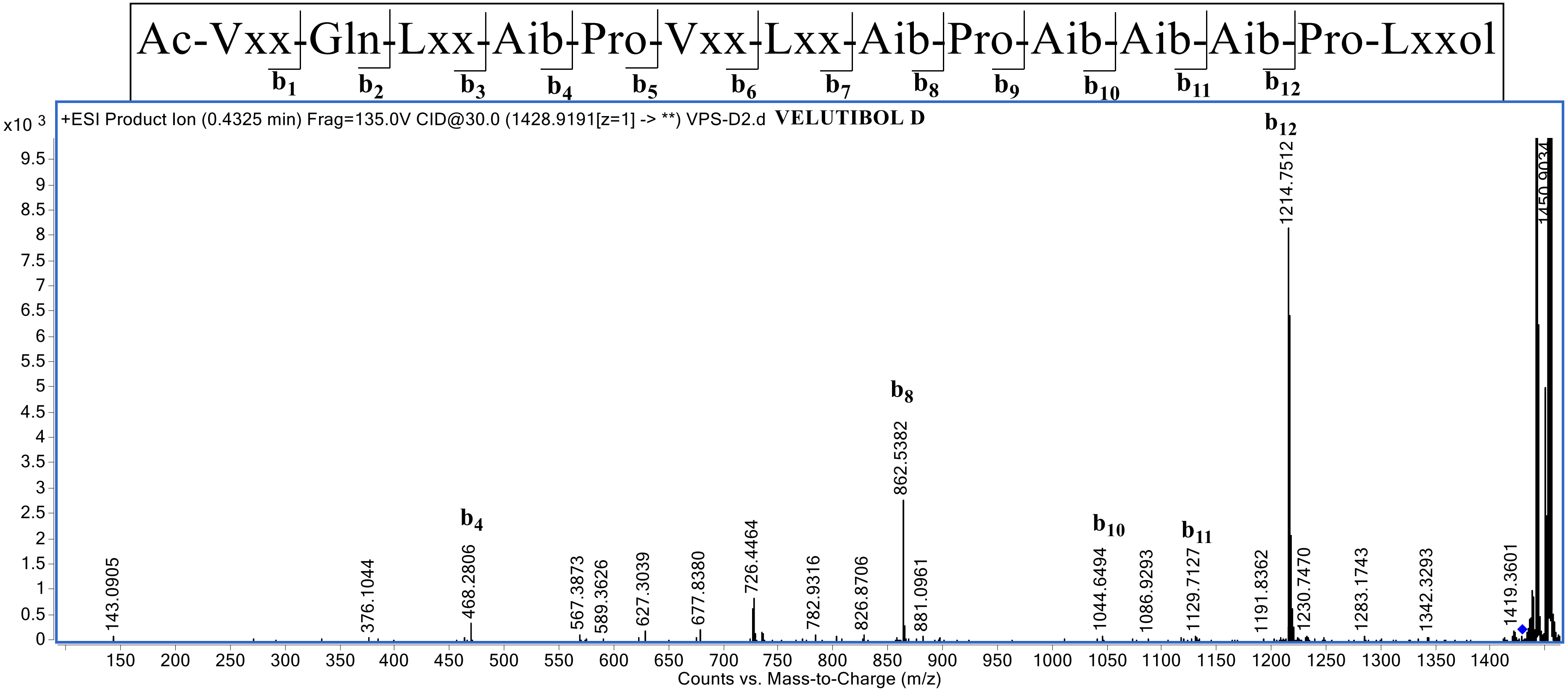

Fig. S26a: MS/MS of *m/z* 1428.9191 parent ion [M+H]$^+$ for compound **4**.

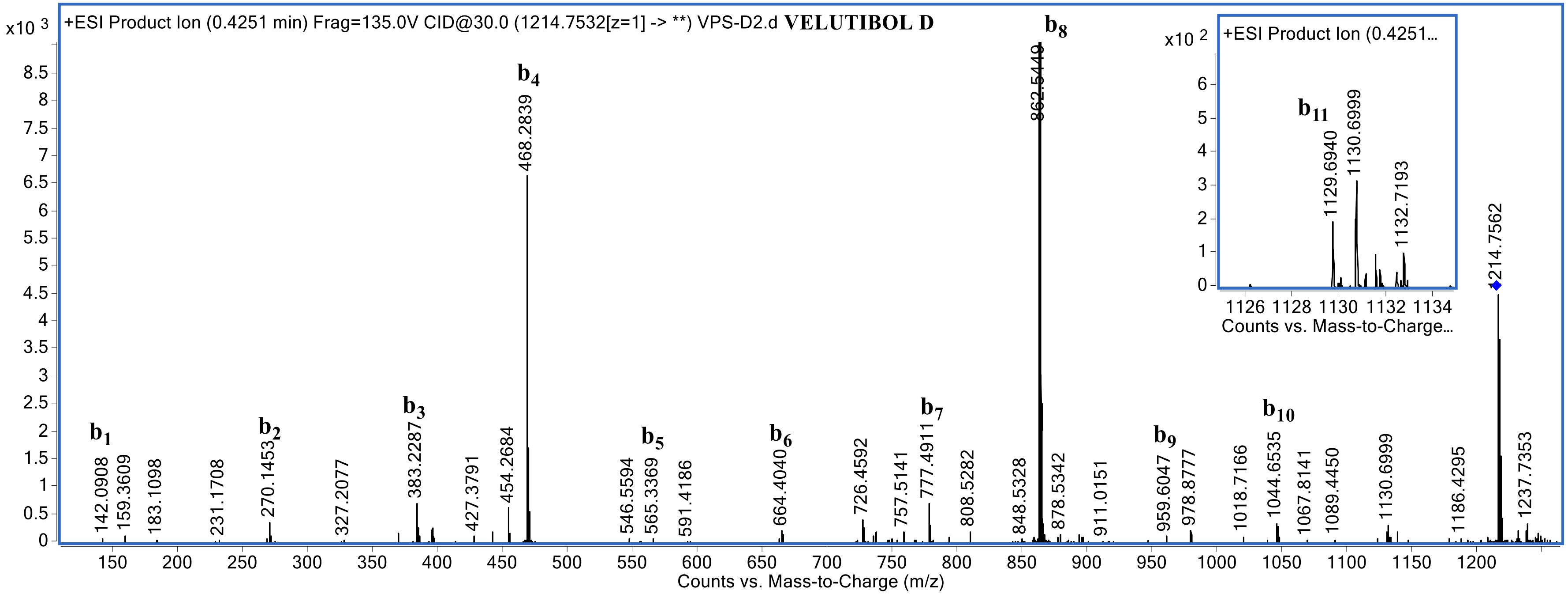

Fig. S26b: MS/MS of *m/z* 1214.7532 daughter ion b_{12} for compound **4.**

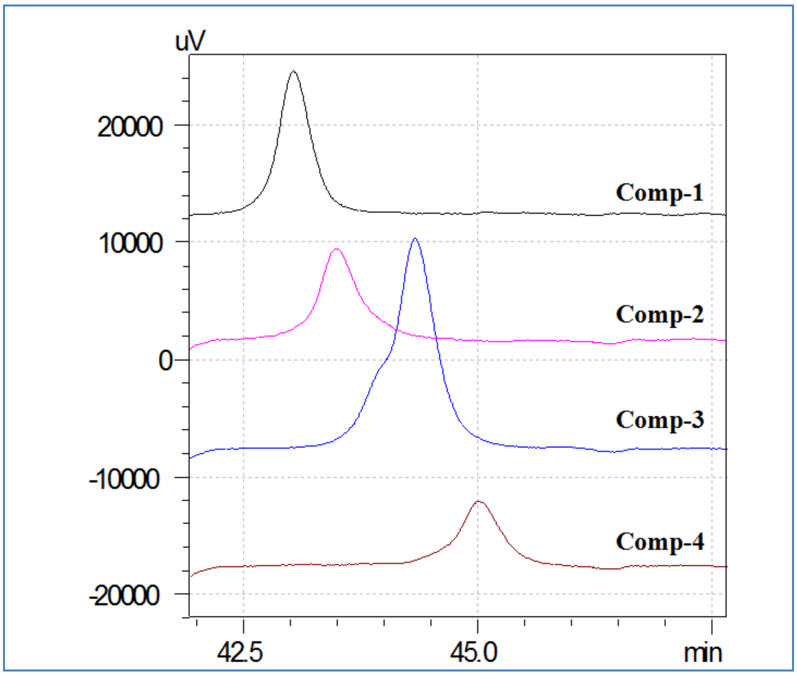

Fig. S27: HPLC overlay chromatogram of compounds **1**, **2**, **3** and **4**.

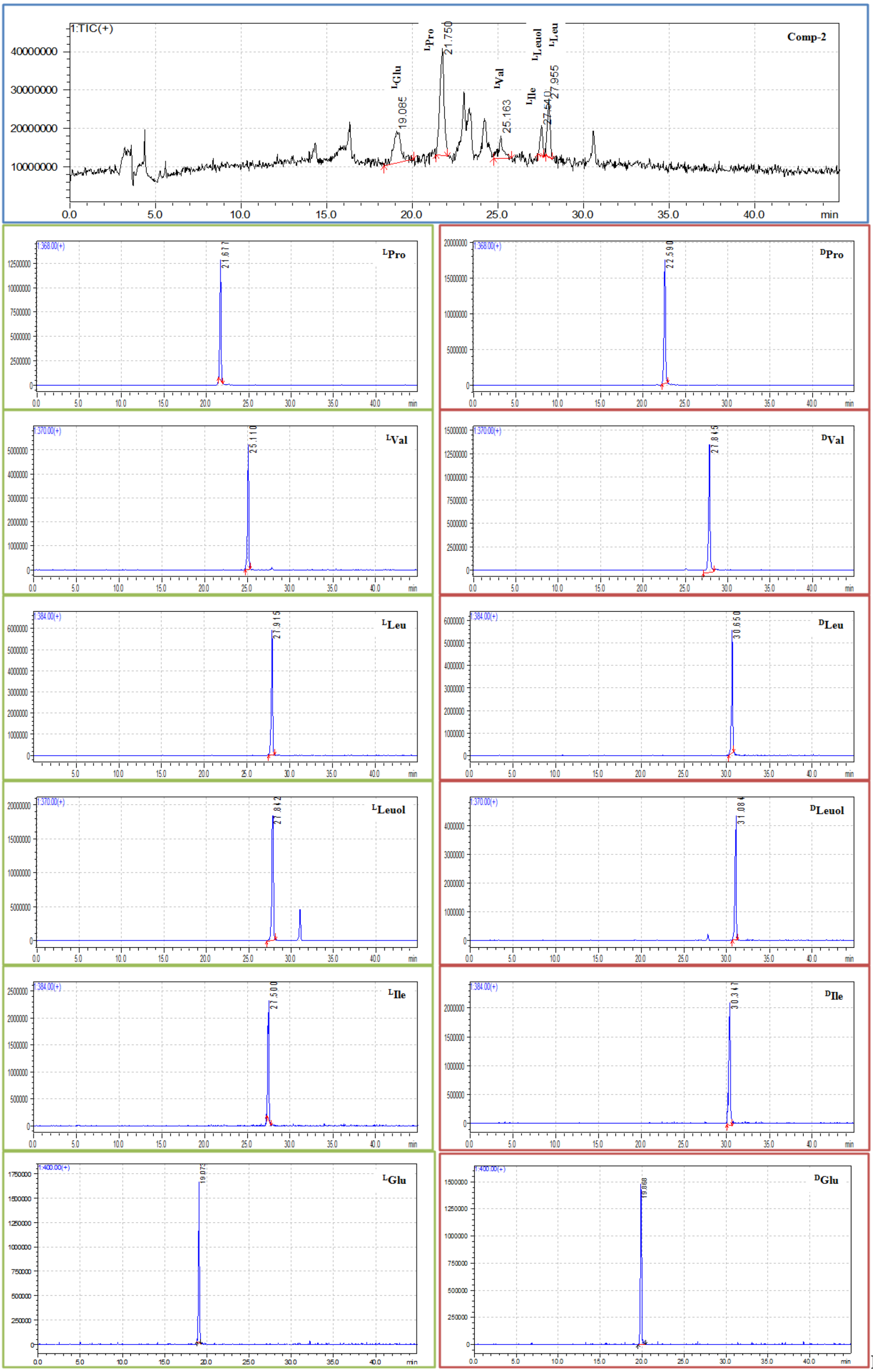

Comp-2
1:TIC(+)
LGlu
LPro
LVal
LIle
LLeuol
LLeu
LPro
DPro
LVal
DVal
LLeu
DLeu
LLeuol
DLeuol
LIle
DIle
LGlu
DGlu

Fig

. **S28**: Marfey's analysis of compound **2**

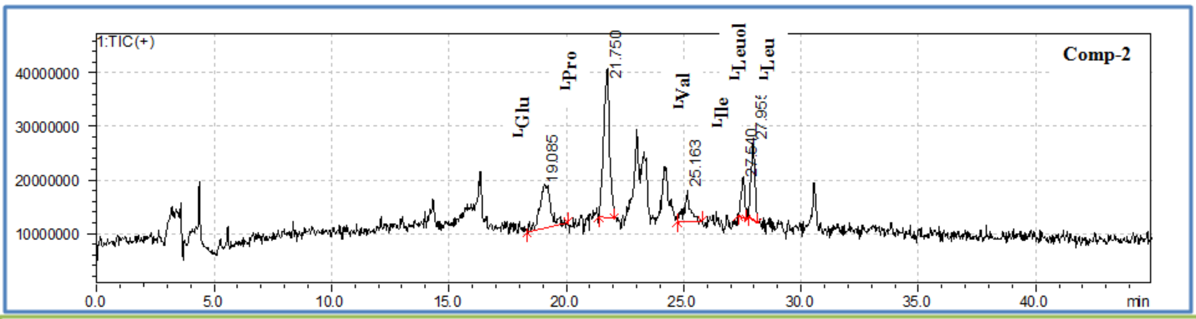

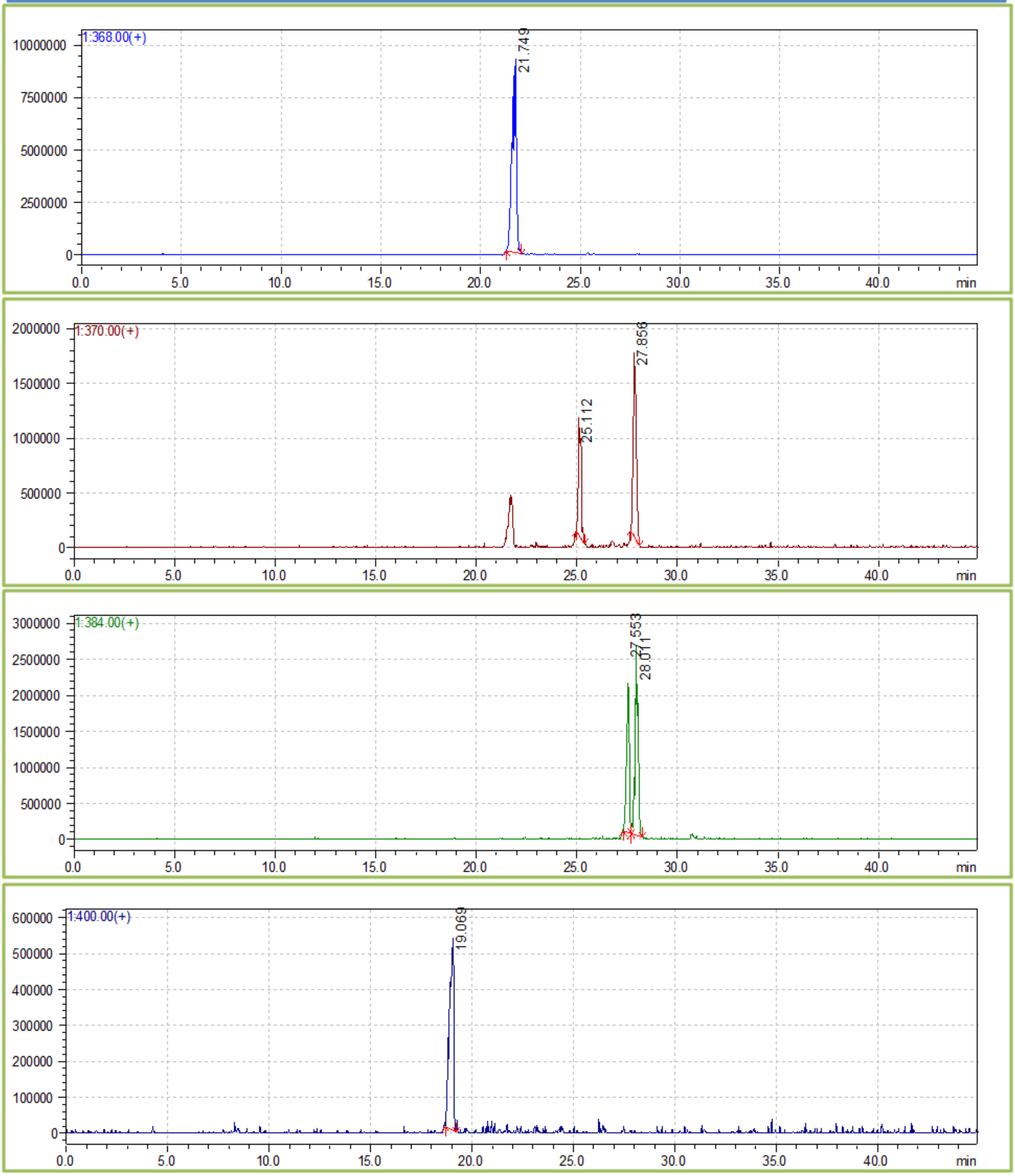

Fig. S29: Extracted ion chromatograms of *m/z* 368, 370, 384 and 400 for compound **2**

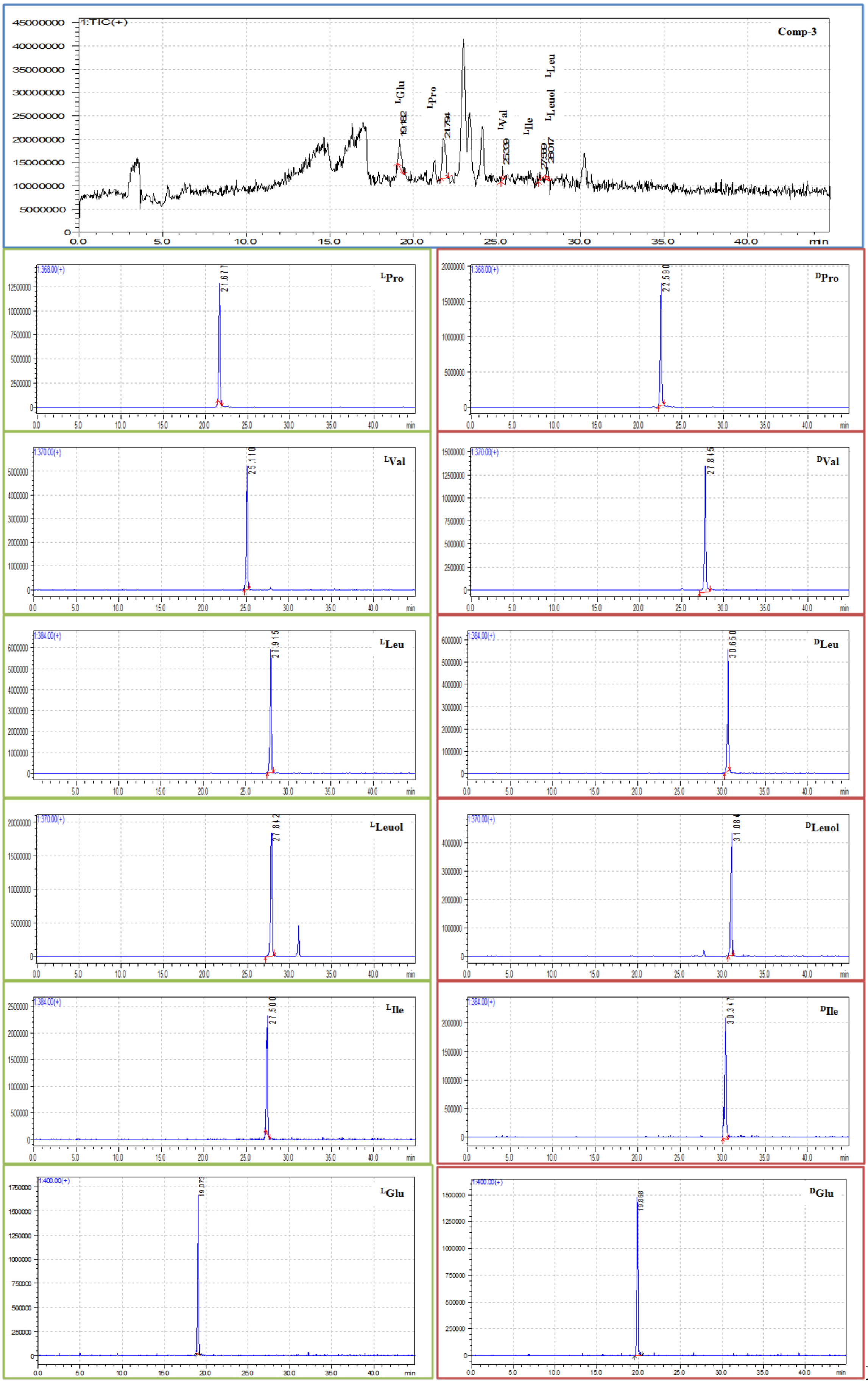

Fig. S30: Marfey's analysis of compound **3**

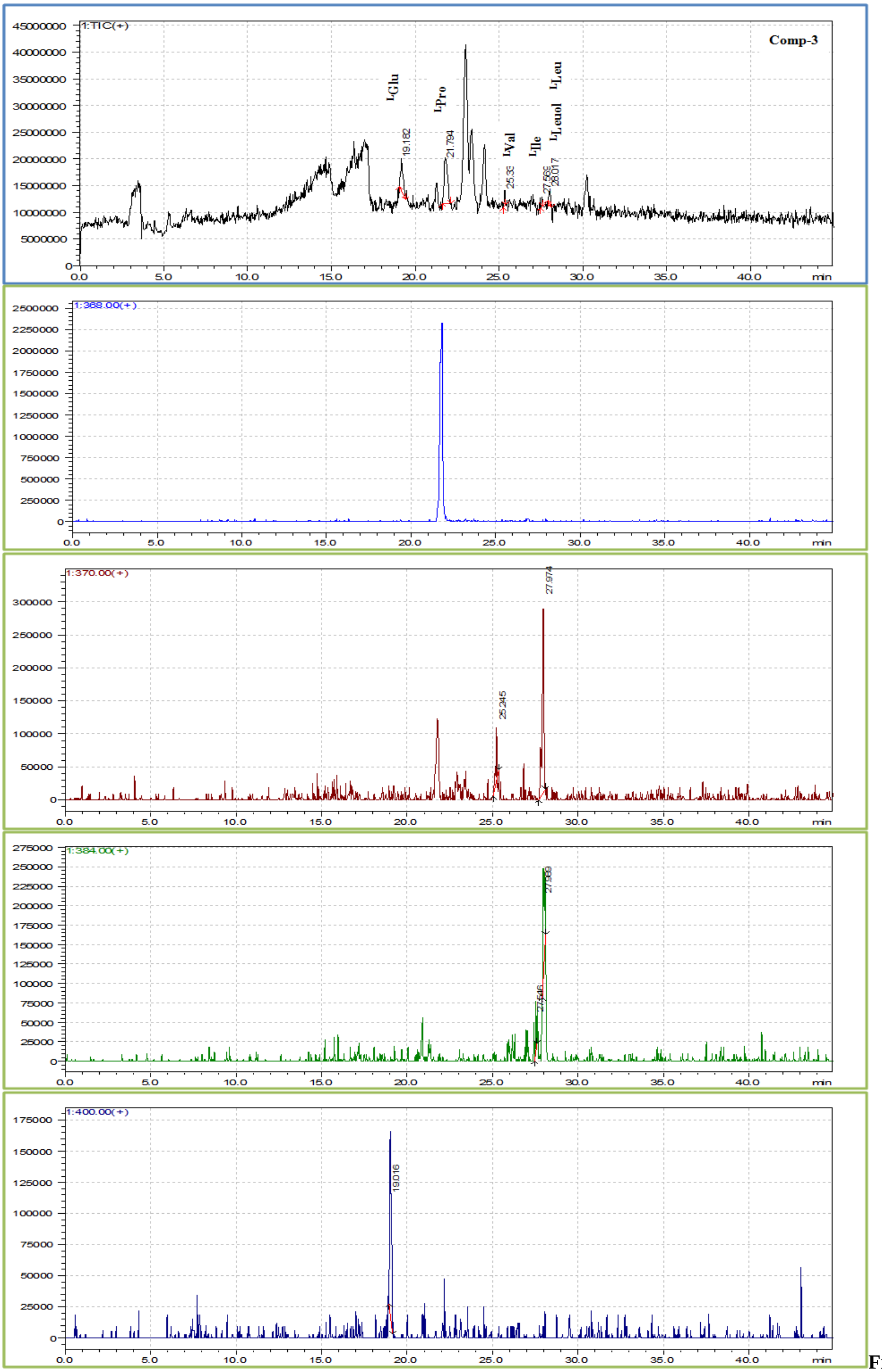

Fig. S31: Extracted ion chromatograms of *m/z* 368, 370, 384 and 400 for compound **3.**

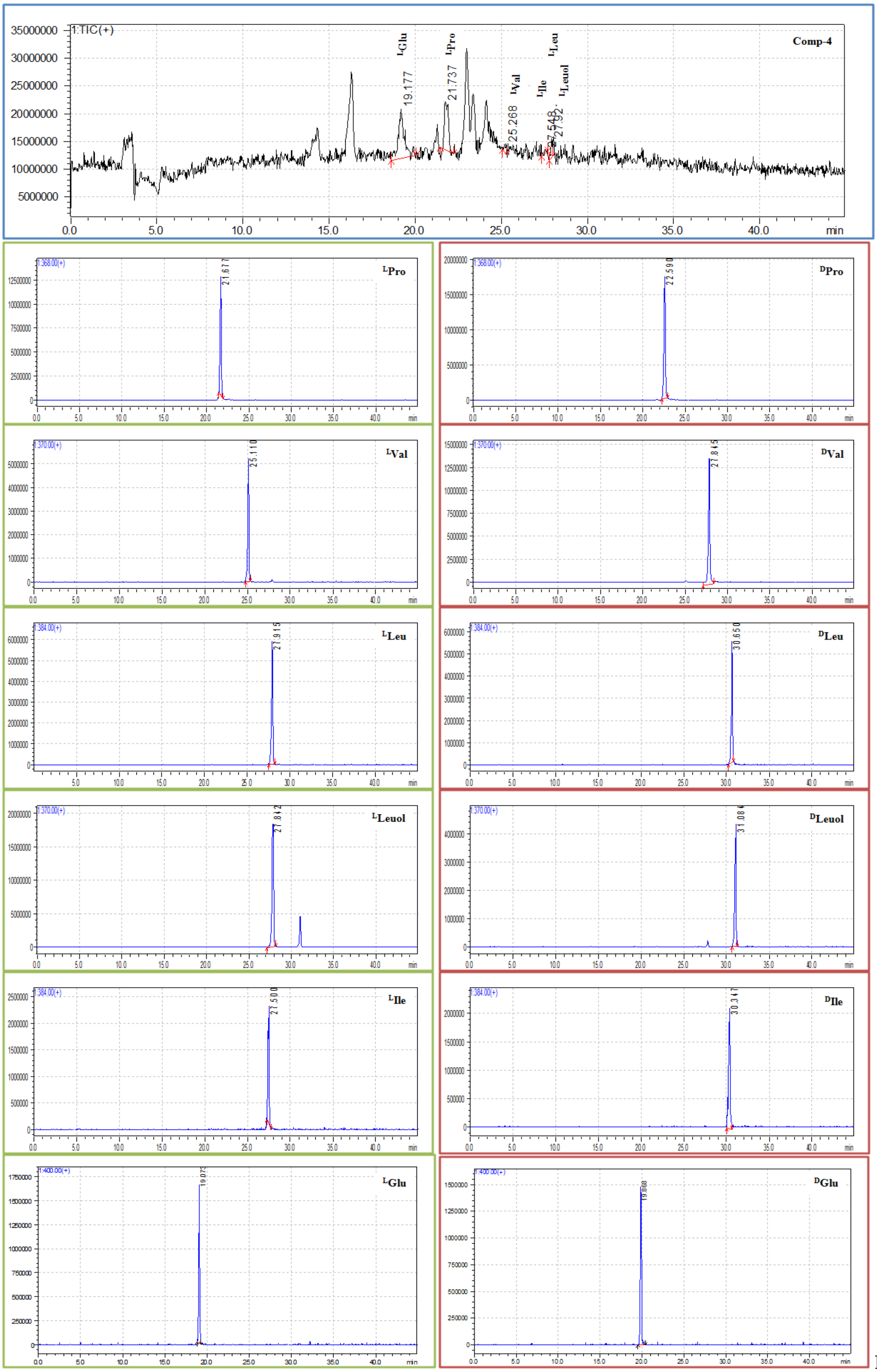

Fig. S32: Marfey's analysis of compound **4**

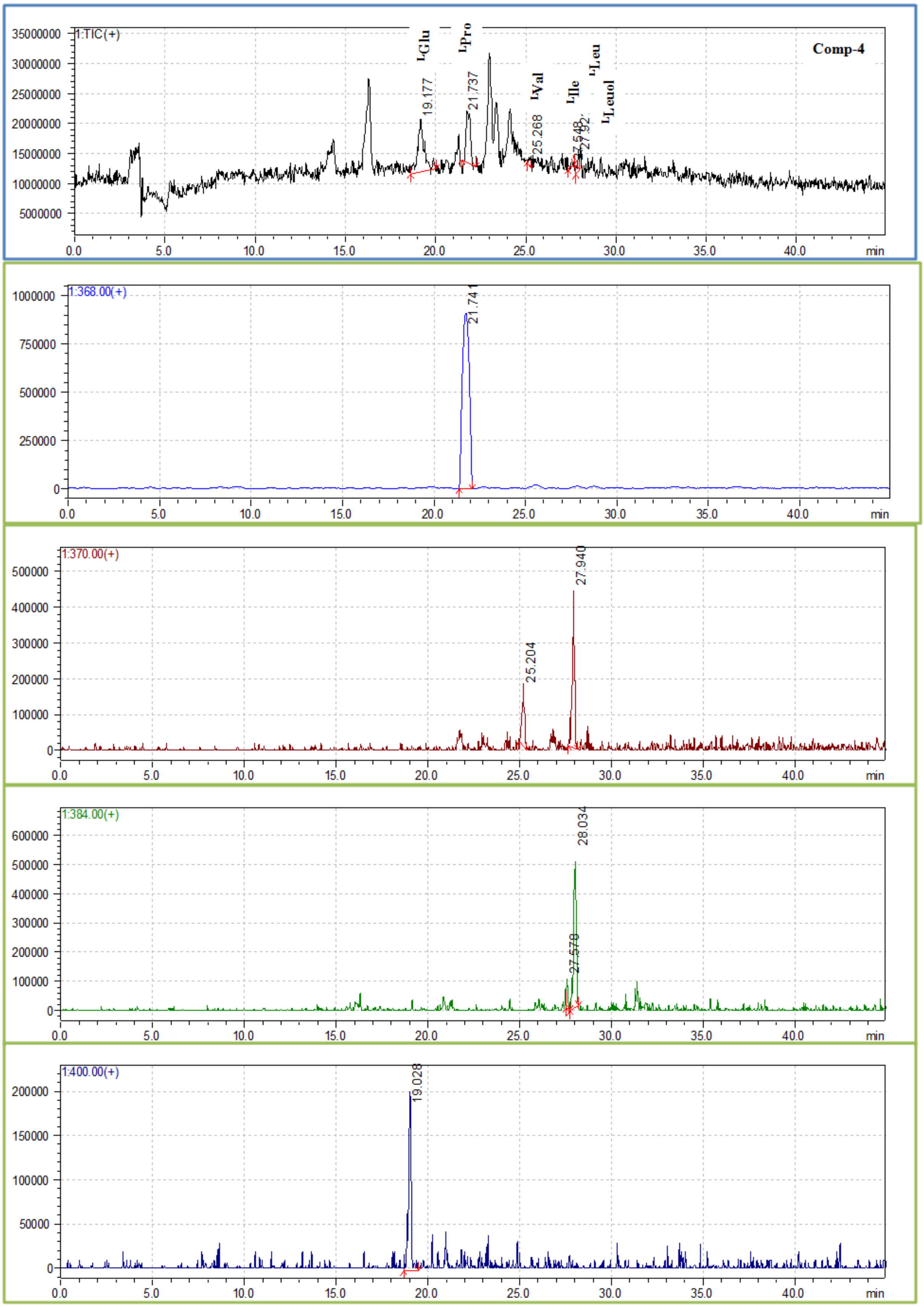

Fig. S33: Extracted ion chromatograms of *m/z* 368, 370, 384 and 400 for compound **4**

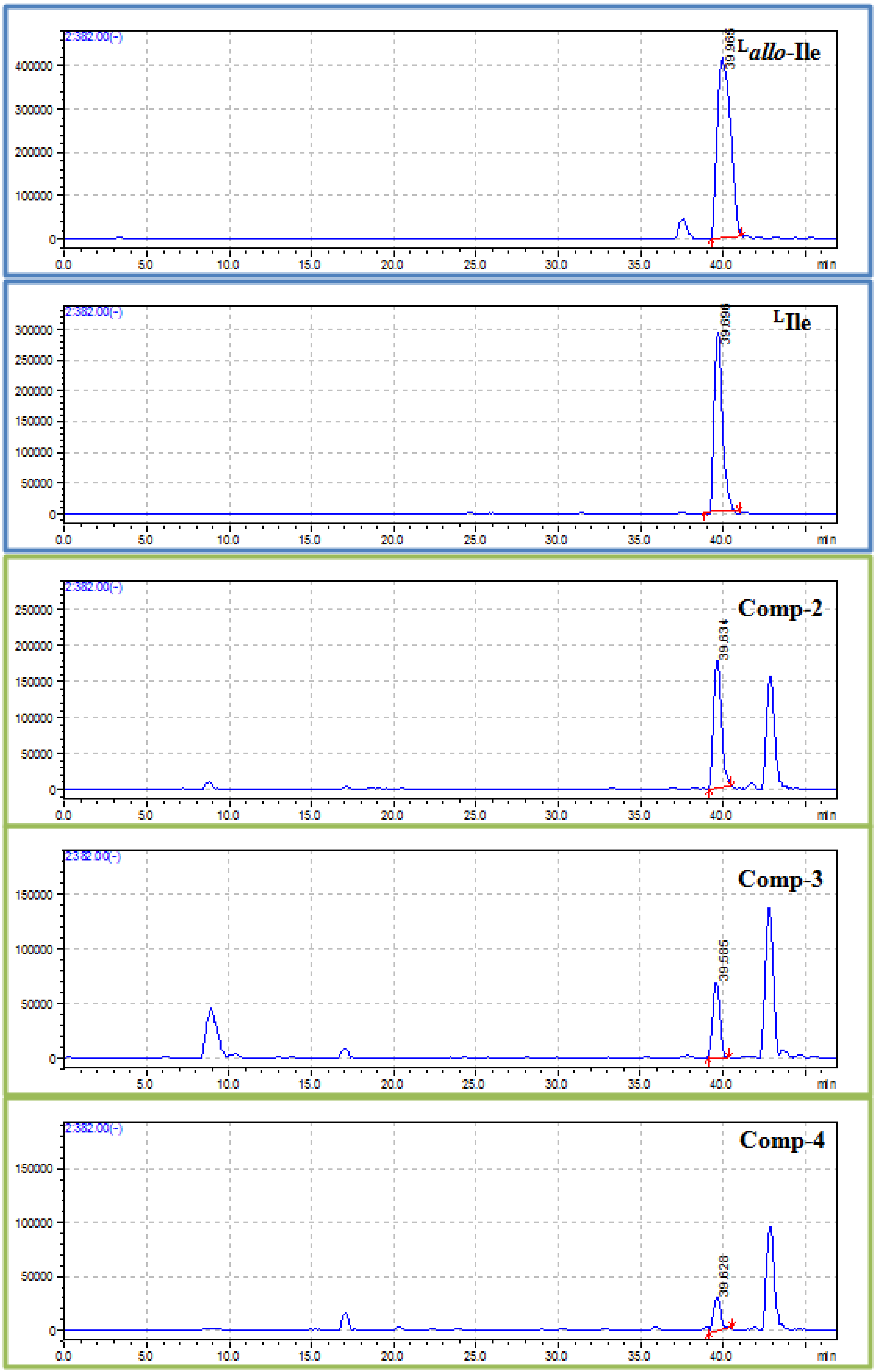

Fig. S34: Extracted ion chromatograms of *m/z* 382 (-ESI) for compound ^{L}*allo*-Ile, ^{L}Ile **2**, **3** and **4** using chiral LCMS

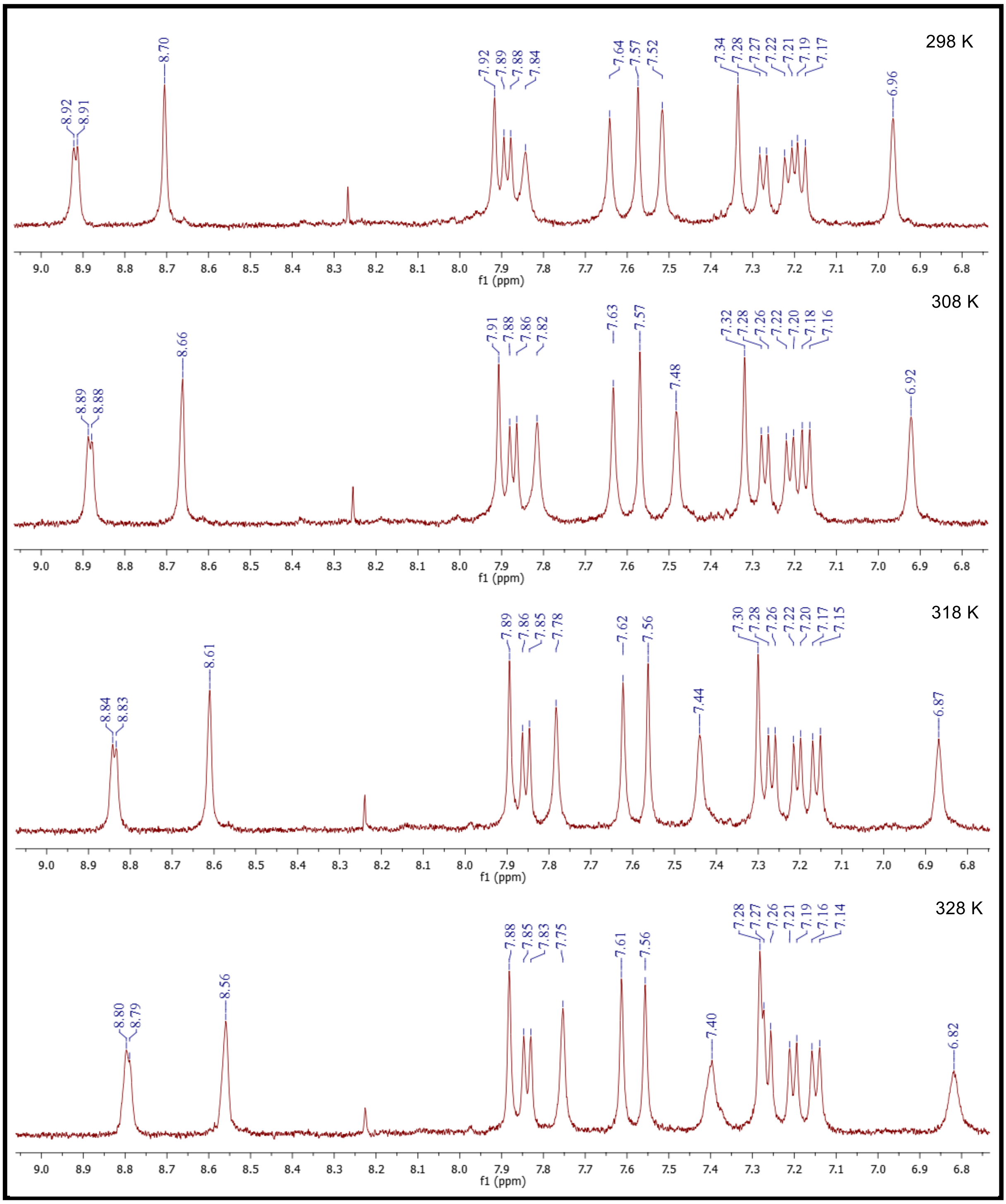

Fig. S35: NMR-VT experiment performed at 298K, 308K, 318K, and 328K for compound **1** in DMSO-d_6 at 400 MHz (Region δ 6.7 – 9.1).

Minimum Inhibitory Concentration of compound against *Mycobacterium tuberculosis*

Data entry *In vitro Mycobacterium tuberculosis* Screening

In house 3, Page No **129,** Dated; 21-05-2015

Organism : *Mycobacterium tuberculosis* $H_{37}Rv$

Media : Middlebrook 7H9 broth supplemented with 10% ADC

Method : Microdilution assay/REMA method

Stock Concentration : 10 mg/ml

Starting concentration : 64 μg/ml

S. No.	Compound Code	MIC in μg/ml
1.	VPS-P1-B (**Velutibol-A**)	32
2.	Rifampicin	0.06

Fig. S36: Anti-tubercular screening report of compound **1**
